# Genomic Characterisation of Carbapenem-Resistant *Klebsiella pneumoniae* Clinical Isolates from a Tertiary-Care Hospital in Türkiye

**DOI:** 10.3390/microorganisms14102402

**Published:** 2026-10-20

**Authors:** Merih Şimşek, Cengiz Demir

**Affiliations:** Department of Medical Microbiology, Faculty of Medicine, Afyonkarahisar Health Sciences University, Afyonkarahisar 03030, Türkiye

**Keywords:** *Klebsiella pneumoniae*, carbapenem resistance, whole-genome sequencing, carbapenemase, plasmid

## Abstract

Carbapenem-resistant *Klebsiella pneumoniae* (CRKP) is a global health threat driven by limited treatment options and high-risk clones. This study aimed to characterise the clonal structure, resistance and virulence determinants and carbapenemase-carrying genetic elements of CRKP isolates from a tertiary-care hospital in Türkiye. Twenty-five isolates, one per patient, were collected between October 2023 and October 2024, tested against EUCAST criteria and sequenced with Oxford Nanopore long reads. Analyses comprised MLST, 2335-locus cgMLST, resistome, virulome, plasmid (MOB-suite), pan-genome (Panaroo) and defence-system profiling. All isolates were carbapenem-resistant, 19 ceftazidime-avibactam-resistant and seven colistin-resistant. Six sequence types and five genetic clusters were identified, with ST147 predominant (n = 13). The commonest carbapenemase profile was *blaNDM-5* plus *blaOXA-48* (n = 13), followed by *blaKPC-3* alone (n = 5) and *blaNDM-7* alone (n = 2). *blaNDM-5* and *blaOXA-48* were mainly carried by conjugative plasmids pAA405 and pAA002, whereas four isolates carried carbapenemases on chromosome-associated or IncX3 elements. *blaNDM-5* co-occurred with the aerobactin operon in 15 isolates. The pan-genome comprised 7377 gene clusters, and isolates within a genetic cluster differed phenotypically and genotypically. Resistance and virulence determinants converged within ST147 and on shared plasmids, so genomic surveillance should consider genetic cluster, element architecture and accessory genome together.

## 1. Introduction

*Klebsiella pneumoniae* is a leading cause of hospital-acquired opportunistic infection, and the spread of carbapenemase-producing, multidrug-resistant strains has made it a global clinical and public health threat. Carbapenems long served as last-line therapy for resistant Gram-negative infections, but the rising incidence of carbapenem-resistant *K. pneumoniae* (CRKP) has sharply curtailed their usefulness. CRKP ranked first among the 24 antibiotic-resistant bacterial pathogens in the 2024 World Health Organization Bacterial Priority Pathogens List [1]. In Türkiye the CRKP rate rose from 29.5% in 2016 to 48.2% in 2020, with the 2021 weighted mean in national surveillance reaching 63.57% [2], making local tracking at genomic resolution essential. The clonal landscape has reshaped rapidly, and high-risk clones keep acquiring resistance under antimicrobial pressure. In a multicentre study of CR-hvKp isolates from 19 Turkish tertiary hospitals (2018–2023), the CR-hvKp proportion rose from 20.7% to 33.1%, and ST2096, dominant in 2018–2019 and largely carrying *blaOXA-232*, was supplanted after 2022 by the KL64 ST147 clone co-carrying *blaNDM-5* and *blaOXA-48* on a 364 kb hybrid plasmid (IncHI1B_pNDM/IncFIB_pNDM) that harboured the previously unreported *rmtF1* and *arr-2* genes [3]. Multiple-carbapenemase production, especially co-occurrence of *blaNDM* with *blaOXA-48*-like determinants, further narrows treatment options. A synthesis of 44 studies (2017–2026) identified ST147, ST101 and ST11 as the principal high-risk clones and described persistent IncL-type 96 kb fusion plasmids and IS10-mediated truncation of *blaNDM-1* as adaptive mechanisms that lower the fitness burden of plasmid carriage while retaining resistance. That review also documented convergence, with dual-carbapenemase producers acquiring colistin determinants (*mcr-1* or *mgrB* alterations) and virulence markers such as *iuc1*, detected also in community, environmental and food reservoirs, so hospital-based surveillance alone is insufficient [4].

Next-generation sequencing resolves what phenotypic testing and conventional typing cannot, because a single dataset yields sequence type, resistance and virulence content, and the replicon and mobility features of plasmids. Long-read platforms such as Oxford Nanopore add the capacity to span repeats and insertion sequences, which can help to place carbapenemase genes on a defined plasmid or chromosomal element. Element architecture governs spread. A study at a Chinese tertiary hospital, where ST11 was dominant (65%), identified in an ST638 isolate a 173,720 bp conjugative plasmid (pKP1226-1) carrying *blaNDM-1*, *blaOXA-232* and T4SS genes, with *blaNDM-1* transfer confirmed by conjugation [5], a type of structure that long-read data can resolve. Genomic data also reconstruct transmission. In an Italian neurorehabilitation outbreak of KPC-Kp among 19 colonised patients, ST307 and ST716 predominated, and genome-based reconstruction redefined an apparent single outbreak as seven independent introductions [6], showing that sequence-type typing cannot replace allelic proximity. These advantages fit the aims of the present study, which required both core-genome resolution of relatedness and element-level localisation of carbapenemase genes.

Genotype–phenotype relationships are not unidirectional, as resistance may also stem from porin loss and efflux changes. In 507 Pakistani isolates, 42% were carbapenem-resistant and *blaNDM-5* was the commonest gene (45.8%), with resistance shaped by OmpK36/OmpK37 mutations (P170M, I128M, N230G, A217S) and efflux linked to *acrR*/*ramR* mutations (P161R, G164A, P157*) [7]. EUCAST notes that the evidence base for predicting susceptibility from WGS remains weak, that ECOFF should be the primary comparator (clinical breakpoints secondary), and that only quality-passing datasets should be used [8]. Carbapenemase type steers therapy directly. Ceftazidime-avibactam inhibits class A (KPC) and class D (OXA-48-like) serine carbapenemases but not class B metallo-β-lactamases (NDM, VIM, IMP) [9]. Combination with aztreonam is therefore advised for metallo-β-lactamase producers, with a modest preference for aztreonam-avibactam over ceftazidime-avibactam plus aztreonam in NDM producers [10]. In a Turkish study, NDM and OXA-48 co-occurred in 37.3% of ceftazidime-avibactam-resistant isolates, the commonest genotypic pattern among carbapenemase carriers [11].

Three gaps persist. First, most Turkish CRKP studies rely on PCR screening or classical MLST, and integrative work resolving clonal structure at the core-genome level, defining element localisation, replicon and mobility by long-read data, and matching these to isolate-level phenotypes is scarce. Second, although ST147 stands out in Türkiye through the *blaNDM-5*/*blaOXA-48* pairing and hybrid plasmids [3], the physical link between these elements and virulence determinants, and phenotypic divergence within a genetic cluster, remain poorly clarified nationally. Third, how genotype–phenotype discordance, for example differing ceftazidime-avibactam or aminoglycoside responses among isolates with the same carbapenemase, should inform clinical reporting is unresolved.

The objective of this study was to characterise the clonal structure, resistance and virulence determinants, and carbapenemase-carrying genetic elements of CRKP isolates from a tertiary-care hospital in Türkiye. Twenty-five isolates, one from each of 25 patients, were sequenced with Oxford Nanopore long reads, which supplies the element-level resolution Turkish data have lacked by revealing the replicon content, size and predicted mobility of carbapenemase-carrying structures, and were analysed at three levels. Core-genome and sequence-type analysis defined clonal relatedness. Evaluating resistance and virulence separately at isolate and element level showed which isolates and elements co-carry the aerobactin operon with *blaNDM-5*. Comparing phenotypic susceptibility with genotypic content at the isolate level made intra-cluster phenotypic divergence visible, gave concrete form to genotype–phenotype discordance in clinical reporting and linked genomic data to treatment decisions where carbapenemase typing guides therapy.

## 2. Materials and Methods

### 2.1. Bacterial Isolates and Identification

Twenty-five carbapenem-resistant *Klebsiella pneumoniae* isolates, recovered from clinical specimens between October 2023 and October 2024 at the Medical Microbiology Laboratory of Afyonkarahisar Health Sciences University Health Practice and Research Hospital, were included in the study. Isolates were recovered from specimens submitted from inpatient wards (n = 15), intensive care units (n = 8) and outpatient clinics (n = 2). The specimens were blood culture (n = 7), urine (n = 7), tracheal aspirate (n = 5), sputum (n = 2), and one each of bronchoalveolar lavage, tissue, wound and peritoneal fluid (Supplementary Table S1). Only one isolate was taken per patient, and repeat isolates from the same patient were excluded. The bacterial isolate was taken as the unit of genomic analysis. Clinical specimens were plated onto blood agar, chocolate agar and EMB agar (RTA Laboratory, Kocaeli, Türkiye) and incubated at 35 °C for 24 h. Suspect colonies were assessed by Gram staining, and species-level identification was performed with the VITEK 2 automated system (bioMérieux, Lyon, France). Isolates were kept in tryptic soy broth containing 15% glycerol at −80 °C until molecular analyses were carried out. The study was conducted in accordance with the Declaration of Helsinki and approved by the Non-Interventional Clinical Research Ethics Committee of Afyonkarahisar Health Sciences University (decision no. 2026/662, dated 4 September 2026. The study title was subsequently amended by decision no. 683, meeting no. 2026/14, dated 11 September 2026). The study was retrospective in design and used anonymized bacterial isolates obtained during routine clinical diagnostics. Therefore, the requirement for informed consent was waived.

### 2.2. Antimicrobial Susceptibility Testing

Antibiotic susceptibilities were determined using the VITEK 2 automated system (bioMérieux, Lyon, France), gradient testing and broth microdilution. For the automated system, bacterial suspensions were adjusted to a 0.5 McFarland turbidity and processed with VITEK 2 antimicrobial susceptibility cards according to the manufacturer’s procedure. Minimum inhibitory concentration (MIC) values for meropenem, amikacin, ciprofloxacin and ceftazidime-avibactam were confirmed by gradient test (Liofilchem, Roseto degli Abruzzi, Italy). For gradient testing, suspensions prepared from fresh cultures and standardised to 0.5 McFarland were spread evenly over the surface of Mueller–Hinton agar (Thermo Fisher Scientific, Basingstoke, UK). Once the surface had dried, the gradient strip was applied. Plates were incubated at 35 ± 1 °C for 16–20 h, after which the point where the inhibition ellipse intersected the strip scale was read as the MIC. Colistin MICs were determined by broth microdilution (Sensititre^TM^ and MIC COL) following the manufacturer’s instructions. *K. pneumoniae* ATCC 700603 served as the reference strain for phenotypic testing. MICs were recorded in mg/L and interpreted according to version 16.1 of the clinical breakpoint tables of the European Committee on Antimicrobial Susceptibility Testing (EUCAST) [12]. Isolates were categorised as susceptible (S), increased exposure (I) or resistant (R), and only isolates in the R category were considered resistant. The breakpoints applied were S ≤ 2 mg/L and R > 8 mg/L for meropenem, R > 8 mg/L for ceftazidime-avibactam and R > 2 mg/L for colistin. Trimethoprim-sulfamethoxazole was interpreted using trimethoprim breakpoints (S ≤ 0.5 mg/L, R > 0.5 mg/L), and the MIC values and categories of all agents are given in Supplementary Table S2. Phenotypic carbapenem resistance was defined as resistance to at least one of ertapenem, imipenem and meropenem.

### 2.3. DNA Extraction and Whole-Genome Sequencing

Genomic DNA was extracted from each isolate using the QIAamp DNA Mini Kit (QIAGEN, Hilden, Germany) according to the manufacturer’s instructions. Genomic DNA concentration was measured with the Qubit dsDNA HS Assay Kit on a Qubit 4 Fluorometer (Thermo Fisher Scientific, Wilmington, DE, USA). Libraries were prepared following the Ligation Sequencing gDNA—Native Barcoding Kit 96 V14 (SQK-NBD114.96) protocol. For each isolate, 400 ng of DNA was treated with NEBNext FFPE DNA Repair and Ultra II End-prep reagents at 20 °C for 5 min and 65 °C for 5 min. Native barcodes (NB01–96) were ligated to the end-repaired DNA with Blunt/TA Ligase Master Mix at room temperature for 20 min, and the reaction was stopped with EDTA. Barcoded samples were pooled, purified with 0.4× AMPure XP beads (Beckman Coulter Life Sciences, Indianapolis, IN, USA), washed twice with Short Fragment Buffer and eluted in 37 µL of nuclease-free water. Adapter ligation was performed with Native Adapter, 2× Rapid Buffer and T4 DNA ligase at 30 °C for 10 min, and after a second AMPure XP purification the library was eluted in 25 µL of Elution Buffer and quantified fluorometrically. An R10.4.1 flow cell was primed with Flow Cell Flush and Flow Cell Tether, and 100 fmol of the adapter-ligated library was loaded with Sequencing Buffer and Loading Beads. Sequencing was carried out on a GridION instrument (Oxford Nanopore Technologies, Oxford, UK) under MinKNOW control, with base calling and demultiplexing performed in Dorado using the super-accuracy model. Raw reads were quality-controlled and adapter-trimmed with Fastplong v0.7.0. Reads with a mean quality below Q10 or shorter than 400 bp were removed, and homopolymer tails exceeding 15 bp were clipped. The Q10 cut-off was the default setting of the workflow and applies to individual reads, whereas the accuracy of the assemblies derives from multi-read consensus (Autocycler consensus and Medaka polishing). Quality and length distributions before and after trimming were summarised with Sequali v1.0.2. Culture composition was profiled against GTDB lineages with sylph v0.9.0 and sylph-tax v1.9.1, and the presence of two or more species each at ≥3.0% at the species level was defined as a mixed culture. No minimum coverage threshold was predefined, and the mean coverage of all assemblies was ≥87.0× (median 120.2×). Raw sequencing reads and genome assemblies were deposited in the NCBI database under BioProject accession PRJNA1538236.

### 2.4. Genome Assembly, Quality Assessment and Annotation

De novo assembly was performed with the Autocycler v0.7.0 consensus framework [13]. Filtered reads were split into two independent subsets, each assembled with Flye v2.9.6 [14], Hifiasm v0.25.0, Myloasm v0.6.0 and Raven v1.8.3 [15]. Assemblies were clustered, trimmed and combined into a single consensus, and contigs shorter than 200 bp were removed with SeqKit v2.13.0. The consensus was polished with Medaka v2.2.2 [16] using the r1041_e82_400bps_bacterial_methylation model. Coverage was computed by alignment with minimap2 v2.29 and summarisation with mosdepth v0.3.14. Assembly quality was evaluated for completeness and contamination with CheckM2 v1.1.0 [17], and gene-content completeness with BUSCO v6.1.0 [18] against the bacteria_odb12.2 and enterobacteriaceae_odb12.2 marker sets. Chromosomal contigs were reoriented to the *dnaA* gene with DNAAPLER v1.4.0, and chimerism and cross-clade contamination were assessed independently with GUNC v1.1.1 [19]. A genome was considered to show potential chimerism when the GUNC clade separation score (CSS) exceeded 0.45 (the default GUNC cutoff), and the CSS value of each genome was recorded. Taxonomic classification was performed with GTDB-Tk v2.7.2 using the GTDB R232 database. Plasmid sequences were separated from chromosomal contigs with MOB-suite v3.1.9 [20] on the basis of relaxase typing, mate-pair formation typing and replication initiator protein analysis. Structural and functional annotation of all chromosomal and plasmid contigs was performed with Bakta v1.12.1 [21] and the full reference database v6.0. Ribosomal RNA genes were assessed with Bakta v1.12.1 and barrnap v1.10.5, and copy numbers were interpreted as a lower bound because they were derived from the assembly. Contig topology (circular or linear) was taken from the Autocycler consensus assembly, and a contig was regarded as completely resolved when it was circular. Plasmids were accordingly classified as complete (circular) or incomplete (linear or unresolved).

### 2.5. Molecular Typing and Phylogenetic Analysis

Classical multilocus sequence typing (MLST) was performed with mlst v2.35.0 using the seven housekeeping genes (*gapA*, *infB*, *mdh*, *pgi*, *phoE*, *rpoB*, *tonB*) of the PubMLST *Klebsiella* scheme. Whole-genome MLST (wgMLST) and core-genome MLST (cgMLST) allele profiles were generated with MiST v1.2.0 using the *Klebsiella* scheme [22]. The *Klebsiella pneumoniae* cgMLST scheme comprises 2358 core genes and 2556 accessory genes. In contrast, the wgMLST scheme includes both the core genes and the accessory genes of the bacterial genome. The wgMLST and cgMLST modes yielded identical total (2335/99%) and called loci, nearest neighbours and pairwise distances, indicating that the scheme implemented in MiST contained no accessory loci in addition to the core loci. Twenty-three loci that could not be called in 3 to 14 of the 25 isolates were excluded, and the shared profile comprised the remaining 2335 loci, each missing in at most two isolates. Population structure was therefore analysed on the basis of the shared 2335-locus allele profile. Pairwise allelic differences and the minimum spanning network were derived from these allele profiles, which were not used as input for phylogenetic inference. Species identification within the *K. pneumoniae* species complex and typing of the capsular (KL) and O-antigen loci were carried out with Kleborate v3.2.4 [23] and confirmed by GTDB-Tk v2.7.2, with all isolates identified as *K. pneumoniae* sensu stricto. A maximum-likelihood phylogeny was inferred from the entropy-filtered core-gene alignment (Section 2.7) with IQ-TREE v2.4.0 [24] under the GTR+F+I model selected by ModelFinder according to the Bayesian information criterion. Branch support was assessed with 1000 replicates of ultrafast bootstrap approximation (UFBoot) supplemented with SH-aLRT values, and the analysis was run with a fixed random seed (42). No additional trimming or gap-filtering was applied to the alignment, and gaps and ambiguous characters were handled by the default treatment of IQ-TREE, in which they are regarded as missing data. Genetic clusters (CL-1 to CL-5) were defined on the basis of cgMLST allele profiles at a threshold of ≤20 pairwise allelic differences, as no universally accepted threshold exists for *K. pneumoniae* [25,26], and the threshold was fixed before the allelic distance matrix was examined. Clusters were defined by single linkage, and these cgMLST-defined clusters denote genetic relatedness only and were not interpreted as evidence of possible or confirmed transmission. Cluster stability was examined at thresholds of ≤15, ≤10 and ≤7 allelic differences. At all three thresholds, CL-1, CL-4 and CL-5 remained intact, CL-2 separated into two groups (three and two isolates), and CL-3 was no longer formed (the two isolates differed by 17 alleles).

### 2.6. Detection of Antimicrobial Resistance and Virulence Determinants

Antimicrobial resistance determinants were sought with AMRFinderPlus v4.2.7 [27] and version 2026-03-24.1 of the NCBI AMR reference database, and acquired resistance genes were additionally screened with ResFinder v4.7.2 [28] using thresholds of at least 80% nucleotide identity and 60% coverage. Hits covering less than the full reference length were retained and flagged as truncated (†) in Supplementary Table S3, and the identity, coverage, functional status and genomic location of each element are given in Supplementary Table S10. All ResFinder calls listed in Supplementary Table S10 had at least 95.0% identity, so a 90% identity threshold would not have changed any call. Species-specific chromosomal resistance mutations were assessed with PointFinder, biocide and disinfectant resistance genes with DisinFinder, and drug-level phenotype predictions were obtained from the ResFinder phenotype table. Results from the two tools were merged by standardising gene names and resolving overlapping detections and duplicate point mutations, and genomic locations were annotated according to the MOB-suite contig classification. Virulence genes were sought with ABRicate v1.4.0 against VFDB using thresholds of at least 80% nucleotide identity and 80% coverage, and detected genes were assigned to adhesion, toxin, secretion system, iron uptake, immune evasion, invasion and regulation classes by keyword matching to VFDB product annotations. Genes reported in the virulence category by AMRFinderPlus were also included, and duplicate records were resolved. Virulence scoring (yersiniabactin, colibactin, aerobactin and salmochelin loci) and profiling of resistance determinants were additionally performed with Kleborate v3.2.4 [23]. For the *mgrB* locus, the region was extracted from the polished assemblies of isolates with colistin-associated calls or incomplete *mgrB* coverage (S06, S08, S11, S14, S16, S18, S22 and S28) and aligned to the full-length *mgrB* sequence of isolate S09 to characterise insertions, deletions and frameshifts at the nucleotide level. These assembly-level findings were not validated with read-level data.

### 2.7. Pan-Genome Analysis

Pan-genome analysis was performed with Panaroo v1.8.0 [29] in strict clean mode using the GFF3 files obtained from Bakta annotation. Genes were clustered at 98% sequence identity and 98% length difference thresholds, and gene families were merged at a 70% sequence identity threshold. Paralog merging was not enabled (default setting), so that potential paralogs were retained as separate gene clusters. Gene clusters were classified by the proportion of genomes in which they were present as core (≥99%), soft-core (95% to <99%), shell (15% to <95%) and cloud (<15%, excluding strain-specific clusters). Clusters found in a single genome were reported as strain-specific. Gene clusters present in ≥95% of genomes (core and soft-core) were aligned with MAFFT [30], and the resulting core-gene alignment was filtered by entropy and used for phylogenetic inference as described in Section 2.5. The final alignment comprised 25 sequences and 3,797,252 nucleotide sites, of which 98.8% were constant and 30,441 were parsimony-informative. Pan-genome and core-genome curves were generated from the Panaroo gene presence–absence matrix with 1000 random permutations of the isolate order. The number of new gene clusters added per genome was modelled with Heaps’ law (n = κN − α) by log-log regression, and the 95% confidence interval of α was obtained by resampling the permutations.

### 2.8. Mobile Genetic Elements, Defence Systems (Including CRISPR-Cas) and Functional Profiling

Insertion sequences were identified with ISEScan v1.7.3 and integrons with IntegronFinder v2.0.6. Defence systems were investigated with DefenseFinder v3.0.0 and model database v3.0.0, and CRISPR-Cas systems with CRISPRCasTyper v1.8.0. Only arrays classified as trusted by CRISPRCasTyper were counted, and repeat and spacer sequences were taken from the tool output, in which the number of spacers is one fewer than the number of repeats. Spacer sequences were compared among arrays by exact sequence identity, and spacer targets were not characterised. Protein secretion systems and surface appendages were assessed with TXSScan via MacSyFinder v2.1.6, and system completeness was calculated from the proportion of detected components of the corresponding model. In the TXSScan models, the type IV secretion system of the MPF_T (mating pair formation T) type involved in protein secretion rather than conjugation is designated as pT4SSt. Prophage regions were identified with geNomad v1.12.0 and database v1.9 and quality-assessed with CheckV v1.1.1 and database v1.5. Regions passing the quality filters were annotated against PHROG with Pharokka v1.10.1 and database v1.8.0, and resistance and virulence genes within phage regions were screened against CARD and VFDB, respectively. Phage lifestyle and putative host predictions were performed with PhaBOX2 v2.1.13 and database v2.1. Biosynthetic gene clusters were sought with antiSMASH v8.0.4 and database v8.0.4. For functional profiling, KEGG orthologues and module completeness were assessed with KofamScan v1.3.0 and database 2025-01, carbohydrate-active enzymes with dbCAN v5.2.9 and database db_v5-2, and peptidases with MEROPS v2.2.5 and database 12.5. Functional assignments to COG, GO and EC categories were performed with eggNOG-mapper v2.1.15 and database 5.0.2. Plasmid maps were generated with custom scripts written by the authors, using the MOB-suite plasmid assignments and the resistance and virulence gene calls described above.

### 2.9. Statistical Analysis

Descriptive statistics were used to summarise the data. Continuous variables, including patient age, MIC values, coverage, genome size and gene counts, were expressed as median, interquartile range (IQR) and range (minimum–maximum), as appropriate. Categorical variables, including sex, specimen type, susceptibility category, sequence type, cgMLST cluster membership and the presence of resistance and virulence determinants, were expressed as frequencies and percentages. Given the descriptive design and the sample size (n = 25), no inferential statistical testing was performed. All statistical analyses were performed in Python 3.12 (Python Software Foundation, Wilmington, DE, USA) using the pandas, NumPy, matplotlib and seaborn libraries. Genotype–phenotype concordance was evaluated descriptively by comparing, for each isolate, the presence of resistance determinants with the corresponding EUCAST susceptibility category, and the findings were interpreted as genotypic associations and not as experimentally demonstrated mechanisms.

## 3. Results

### 3.1. Isolate Collection and Clinical Characteristics

A total of 25 carbapenem-resistant *Klebsiella pneumoniae* isolates, one per patient, were obtained from 25 different patients between October 2023 and October 2024. The patients had a median age of 64 years, an interquartile range of 42–76 years and an age range of 1–88 years. Twelve patients (48%) were 65 years or older, and 20 (80%) were male. The only paediatric patient in the study was S16, aged 1 year. Twenty-three isolates (92%) came from inpatients, while two (S09 and S14) were recovered from urine samples taken at outpatient presentation. Eight patients (32%) were being followed in the intensive care unit at the time of sampling. The most frequent specimen types were blood culture and urine (n = 7, 28% each), followed by tracheal aspirate (n = 5), sputum (n = 2), and one each of bronchoalveolar lavage, tissue, wound and peritoneal fluid. Clinical and demographic data are presented in Supplementary Table S1.

### 3.2. Phenotypic Antimicrobial Susceptibility

**Figure 1:**
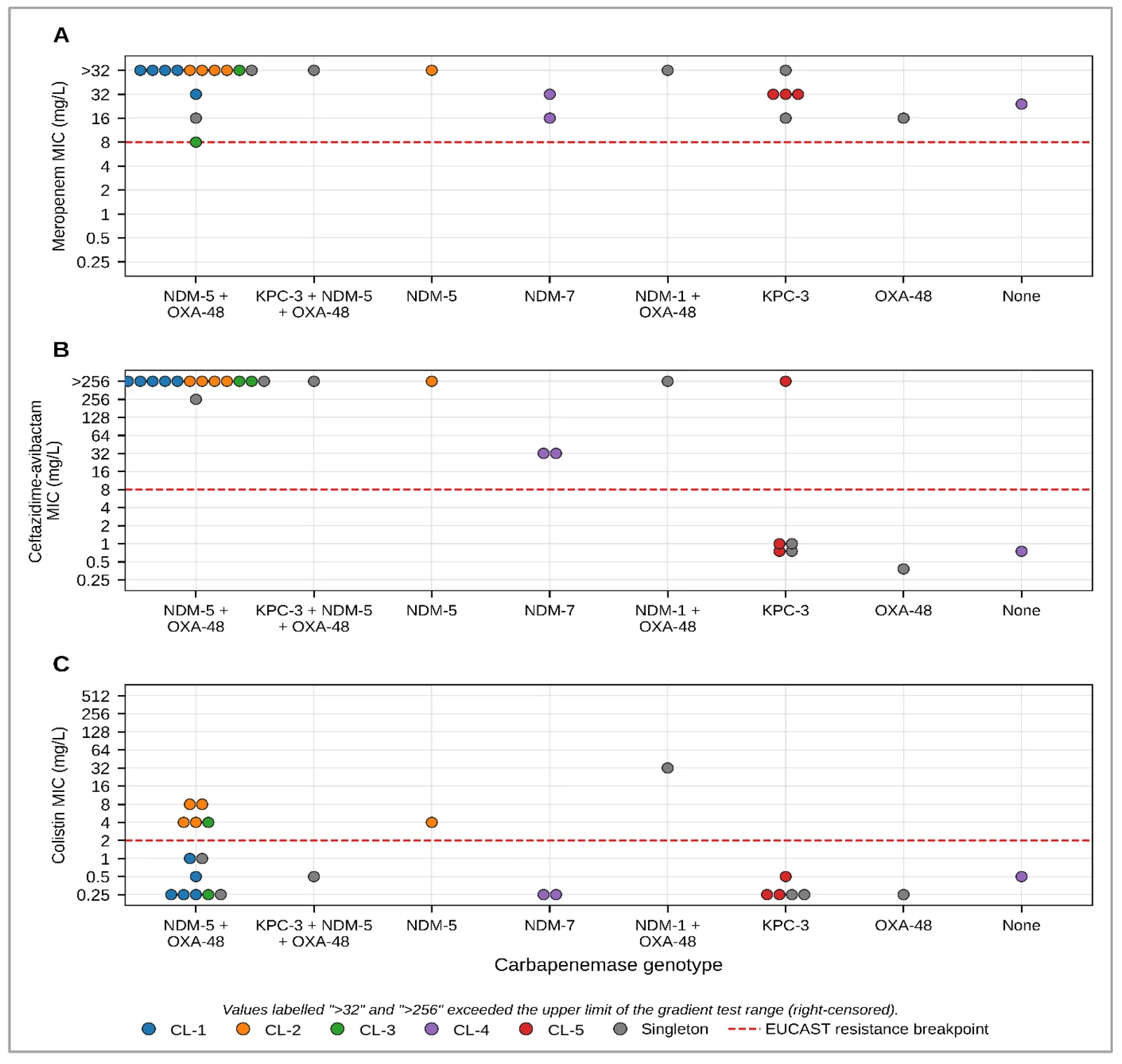
Distribution of antimicrobial minimum inhibitory concentration (MIC) values by carbapenemase genotype. (**A**) meropenem. (**B**) ceftazidime-avibactam. (**C**) colistin. Each point represents one isolate. Colours indicate genetic cluster membership (CL-1–CL-5), and grey indicates isolates not assigned to any cluster (Singleton). CL, genetic cluster. Red dashed lines indicate the EUCAST resistance breakpoints (R > 8 mg/L for meropenem and ceftazidime-avibactam and R > 2 mg/L for colistin), and points above the lines represent resistant isolates. The meropenem MIC of S06 (8 mg/L) lies on the breakpoint line and corresponds to the susceptible, increased exposure (I) category. Meropenem and ceftazidime-avibactam results exceeding the upper limit of measurement are given as >32 and >256 mg/L, respectively, and placed at a separate upper tier for display purposes. Isolate-level MIC values and their categorical interpretations according to EUCAST are presented in Supplementary Table S2.

All isolates were resistant to ertapenem (MIC > 4 mg/L), imipenem (MIC > 8 mg/L), amoxicillin-clavulanate, piperacillin-tazobactam, cefuroxime, cefoxitin, ceftazidime, ceftriaxone, cefepime and ciprofloxacin (MIC > 32 mg/L). Meropenem resistance was found in 24 isolates (96%), with MICs of 8 to >32 mg/L, and the MIC exceeded the upper limit of the test in 14 isolates (56%) (Figure 1A). S06 (meropenem MIC 8 mg/L, susceptible, increased exposure category) was classified as carbapenem-resistant on the basis of its ertapenem and imipenem resistance. Colistin resistance was found in seven isolates (28%), six of them ST147 (S06, S14, S18, S19, S20, S22) and one ST101 (S16), with MICs of 4 to 32 mg/L in resistant and 0.25 to 1 mg/L in susceptible isolates (Figure 1C). Amikacin resistance was detected in 14 isolates (56%), with MICs of >256 mg/L in 13 and 16 mg/L in S13, whereas the 11 susceptible isolates had MICs of 0.38 to 8 mg/L. Ceftazidime-avibactam resistance was detected in 19 isolates (76%), with MICs of 32 mg/L in the *blaNDM-7*-carrying S05 and S21 (ST17, CL-4), 256 mg/L in S07 (ST307, singleton) and >256 mg/L in the other 16 resistant isolates (Figure 1B). All 18 NDM-carrying isolates were resistant. Among the seven isolates without an NDM gene, only S15 (*blaKPC-3* alone) was resistant, and the other six were susceptible (S04 without a carbapenemase gene, S12 with *blaOXA-48* alone and the *blaKPC-3*-only isolates S13, S17, S27 and S28), with MICs of 0.38 to 1 mg/L. S15 and S17 carried *blaKPC-3* on plasmids of the same size (69,799 bp), and S15, S17 and S27 (all ST307, CL-5) had identical *ompK35* and *ompK36* alterations. Trimethoprim-sulfamethoxazole resistance was detected in 21 isolates (84%). In S13, S17, S27 and S28, the MIC was reported as ≤20 mg/L, the lower limit of the automated system, and these isolates could not be categorised according to the EUCAST breakpoint. Isolate-level MICs and their EUCAST categories are presented in Supplementary Table S2.

### 3.3. Genome Assembly and General Genome Features

In the assemblies generated from Oxford Nanopore long reads, the chromosome was circular in 24 isolates and linear in S06. Mean coverage ranged from 87.0× to 255.1×, with a median of 120.2×. Total genome size ranged from 5,409,674 to 6,124,210 bp (median 5,929,149 bp), and chromosome size from 5,256,006 to 5,629,521 bp. The smallest chromosome was found in S04 and the largest in S28. GC content was 56–57%, and the number of contigs per assembly was 2–7. CheckM2 completeness was 100.0% in all isolates, with contamination estimates of 0.03–1.23%. BUSCO completeness ranged from 98.7% to 99.1% with the enterobacteriaceae_odb12.2 marker set and from 97.4% to 98.3% with the bacteria_odb12.2 marker set, and GTDB-Tk Average Nucleotide Identity (ANI) ranged from 98.92% to 99.13%. GUNC flagged 10 of the 25 genomes (S06, S08, S14, S19, S20, S22, S23, S24, S25 and S26), all of which belonged to ST147, with clade separation scores (CSS) of 0.61 to 0.69, above the threshold of 0.45, whereas the CSS was 0 in the other 15 genomes. In every flagged genome the flag was raised at the family level only, and the genome passed at the kingdom, phylum, class, order, genus and species levels. The contamination portion was 0.02 in each flagged genome, and 92.3% to 93.1% of the mapped genes were assigned to Enterobacteriaceae. Genes assigned to other families were present on every contig of the flagged genomes, and the values for each genome are given in Supplementary Table S9. The flag did not follow genetic relatedness. Three ST147 genomes (S09, S10 and S18) were not flagged, although S09 differed from the flagged S24 and S25 by 5 and 6 alleles and S18 from the flagged S22 and S14 by 1 and 3 alleles. The CheckM2 contamination estimate was 0.90% to 1.23% in the flagged genomes and 0.03% to 1.06% in the other 15, and it therefore did not separate the two groups. Assembly graphs and read support across contig junctions were not examined, so the GUNC signal was neither confirmed nor excluded as a source of assembly error. Assembly, quality and pan-genome metrics are presented in Supplementary Table S5.

### 3.4. Population Structure: MLST, Capsule/O-Antigen Loci and Genetic Clusters

**Figure 2:**
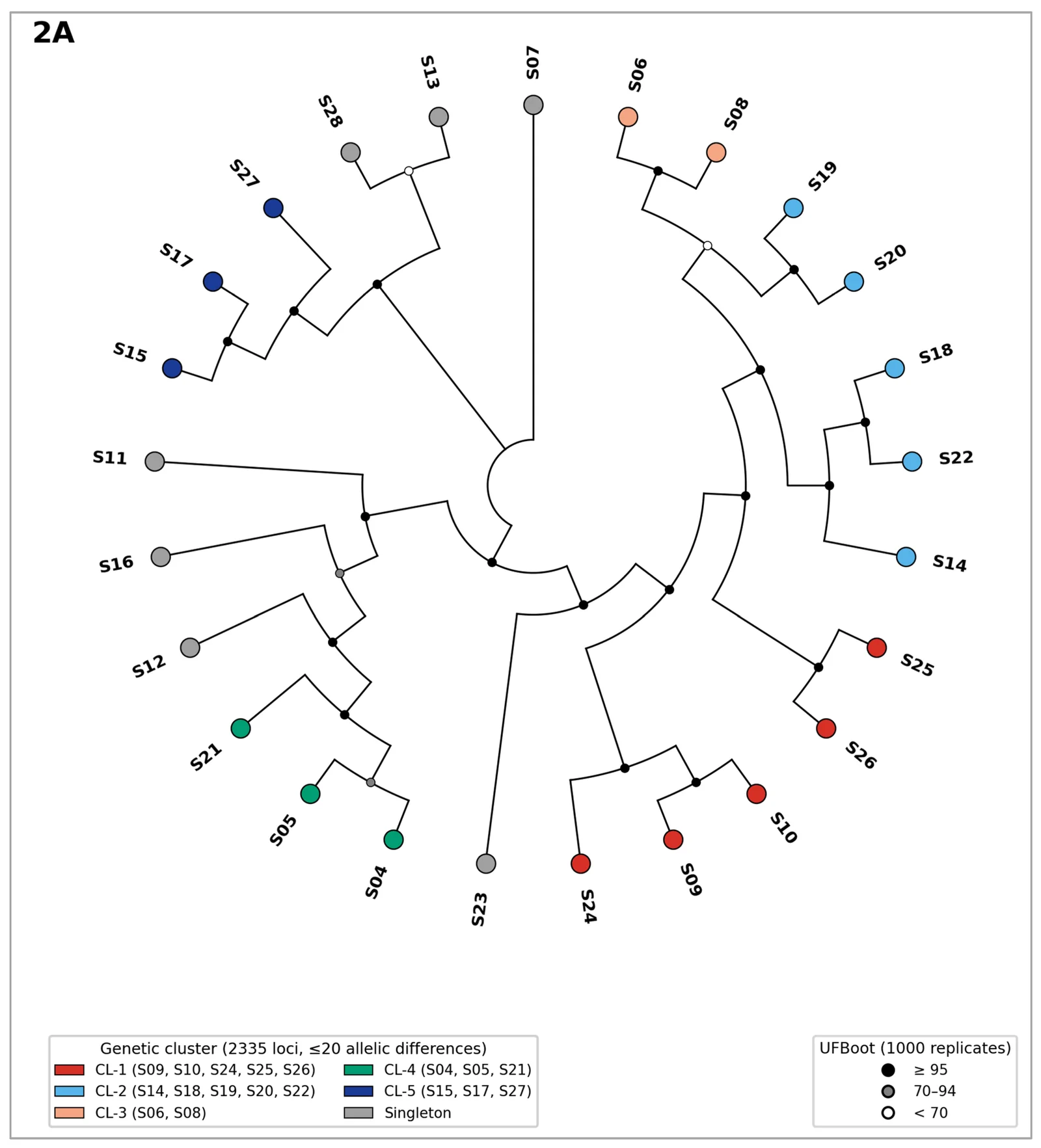

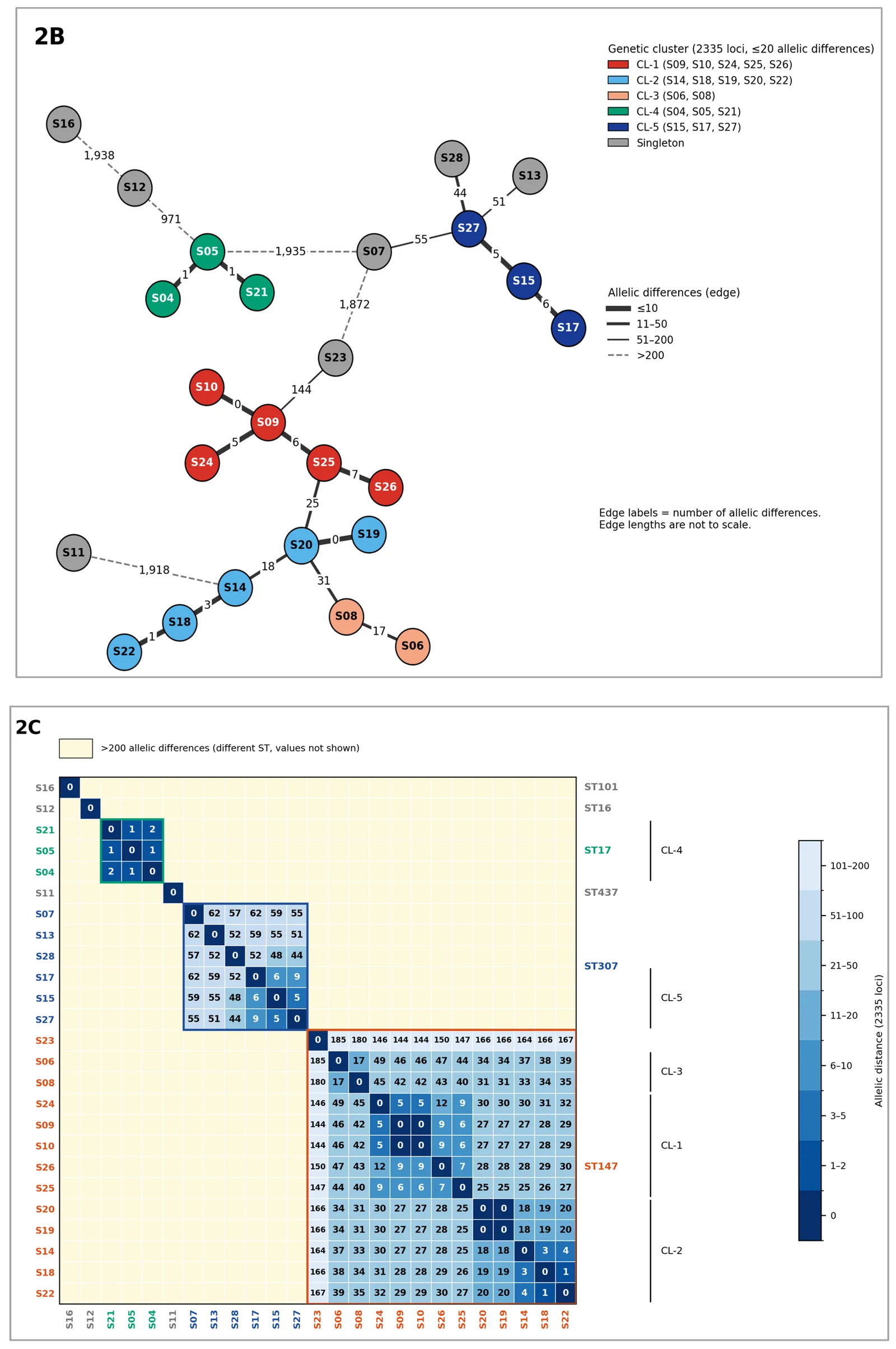
Genetic relationships and cluster structure of the isolates based on the core-gene alignment and 2335–locus allele profiles. (**A**) Maximum-likelihood phylogeny inferred with IQ–TREE v2.4.0 from the core-gene alignment (3.797.252 sites, 30.441 parsimony-informative sites) under the GTR+F+I model and shown as a cladogram, with branch lengths not to scale. Circles on internal nodes indicate ultrafast bootstrap support from 1000 replicates. (**B**) Minimum spanning network of the allele profiles. (**C**) Heatmap of pairwise allelic differences between isolates. Genetic clusters were defined using a threshold of ≤20 allelic differences. In panels (**A**,**B**), colours indicate genetic cluster membership, and grey indicates isolates not assigned to any cluster. In panel (**B**), the numbers on the connecting lines indicate allelic differences. Line lengths are not scaled to allelic differences. In panel (**C**), isolate label colours indicate ST groups, and the brackets on the right indicate genetic clusters. Cells show allelic differences. Cells with >200 allelic differences are shaded light yellow and their values are not shown. CL, genetic cluster. ST, sequence type.

The isolates were distributed across six sequence types (STs). ST147 accounted for 13 isolates (52%), ST307 for six (24%) and ST17 for three (12%), while ST437, ST16 and ST101 were each represented by a single isolate. KL64/O2α was identified in ST147 isolates, KL102/O2β in ST307 isolates and KL147/O2β in ST17 isolates. S11 carried KL47/O13, S12 KL51/O3γ and S16 KL17/O2α. The *wzi* alleles were *wzi64* for ST147, *wzi173* for ST307 and *wzi349* for ST17 (Table 1, Supplementary Table S7). Genetic clusters (CLs) were defined by single-linkage clustering of pairwise allelic differences at a threshold of ≤20 alleles, which was selected before the allelic distance matrix was examined. Twenty-three loci that could not be called in 3 to 14 of the 25 isolates were excluded, and the remaining 2335 loci, each missing in at most two isolates, were used for the allele matrix and the distances. Five CLs were identified. The number of called loci ranged from 2239 to 2333. Eighteen isolates (72%) fell within clusters, and seven remained singletons. CL-1 comprised S09, S10, S24, S25 and S26, CL-2 comprised S14, S18, S19, S20 and S22, CL-3 comprised S06 and S08, CL-4 comprised S04, S05 and S21, and CL-5 comprised S15, S17 and S27. S07, S11, S12, S13, S16, S23 and S28 were classified as singletons. No allelic differences were found within the S09/S10 and S19/S20 pairs, whereas S06 and S08 differed at 17 alleles. The nearest neighbour of S23 was S09, with 144 allelic differences between them The largest within-cluster distance was 12 alleles in CL-1, 20 in CL-2, 17 in CL-3, 2 in CL-4 and 9 in CL-5. No pairwise distance fell between 21 and 24 alleles, and the smallest distance between members of different clusters was 25 alleles (CL-1 and CL-2). The clusters describe genetic relatedness and do not establish transmission. Cluster assignment at thresholds from ≤6 to ≤50 alleles is given in Supplementary Table S8, and the number of clusters ranged from 15 at ≤6 to 9 at ≤50. CL-4 and CL-5 were unchanged from ≤6 to ≤20 and CL-1 from ≤10 to ≤20, whereas S26 separated from CL-1 at ≤6. At ≤15 and below, CL-2 split into S14, S18 and S22 and into S19 and S20 (18 alleles between the two groups), and CL-3 was not formed because S06 and S08 differ at 17 alleles. At ≤25, CL-1 and CL-2 merged into a single cluster of ten isolates. (Figure 2A–C, Supplementary Figures S1 and S2).

**Figure 3:**
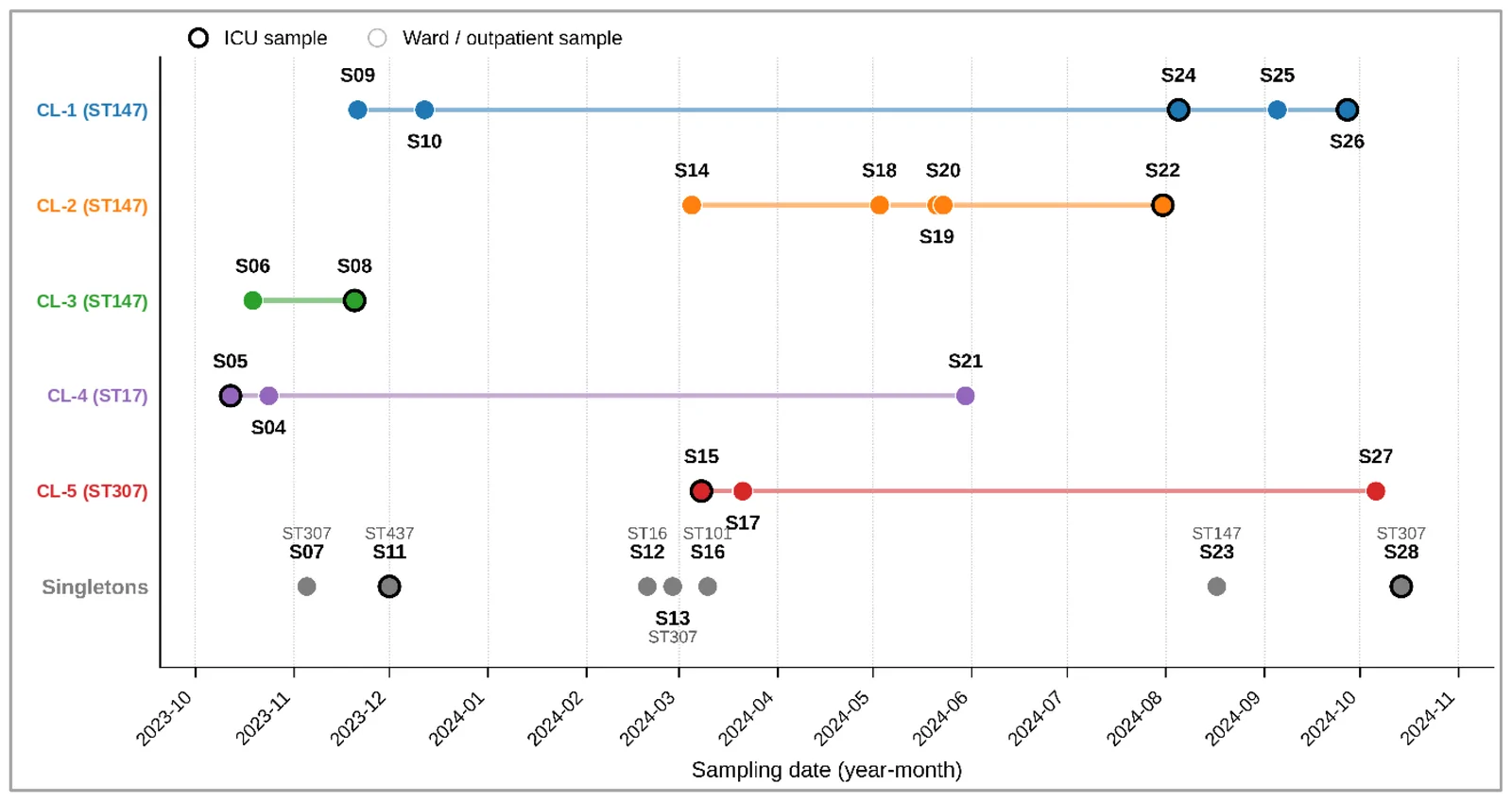
Temporal distribution of 25 carbapenem-resistant *Klebsiella pneumoniae* isolates by genetic cluster, October 2023 to October 2024. Each circle represents one isolate positioned according to its sampling date. Rows correspond to genetic clusters (CL-1 to CL-5) defined using 2335-locus allelic profiles with a threshold of ≤20 allele differences. The sequence type (ST) of each cluster is given in parentheses. Horizontal lines indicate the interval between the first and last sampling dates within each cluster and do not imply continuous transmission or direct patient-to-patient spread. Grey circles in the bottom row indicate singleton isolates not assigned to any cluster, with their individual STs shown above the labels. Circles with a black outline indicate isolates recovered from intensive care unit patients. Circles with a light outline indicate isolates from ward or outpatient samples.

CL-1 isolates were obtained between November 2023 and September 2024, CL-2 isolates between March and July 2024, CL-3 isolates between October and November 2023, CL-4 isolates between October 2023 and May 2024, and CL-5 isolates between March and October 2024. Of the 18 clustered isolates, six were obtained from intensive care units and 12 from wards or outpatient clinics (Figure 3).

### 3.5. Resistome: Carbapenemases, β-Lactamases and Other Resistance Determinants

At least one carbapenemase gene was detected in 24 isolates (96%), and 15 (60%) carried more than one. The most frequent profile was *bla_NDM-5_* plus *bla_OXA-48_* (n = 13). Other profiles were *bla_KPC-3_* alone in five ST307 isolates, *bla_NDM-7_* alone in two ST17 isolates, *bla_NDM-1_* plus *bla_OXA-48_* in S16 (ST101, singleton), *bla_OXA-48_* alone in S12 (ST16, singleton), *bla_NDM-5_* as the only carbapenemase gene in S18 (ST147, CL-2), and *bla_NDM-5_*, *bla_KPC-3_* and *bla_OXA-48_* together in S23 (ST147, singleton). In S18, the only carbapenemase gene refers to carbapenemases alone, because this isolate also carried the complete aerobactin operon on the same pAA405 plasmid as *bla_NDM-5_*, *ompK36* and *ompK35* alterations and an *mgrB* insertion, reported by Kleborate as c.T132del (Tables 3 and 4). NDM-type genes were found in 18 isolates (72%), OXA-48 in 16 (64%) and KPC-3 in six (24%). The NDM alleles were NDM-5 (n = 15), NDM-7 (n = 2) and NDM-1 (n = 1).

S04 (ST17, CL-4) was the only isolate without a carbapenemase gene. It had a meropenem MIC of 24 mg/L and carried *bla_CTX-M-15_* together with *ompK36*, *ompK37* and *acrR* alterations. S05 and S21, which shared its genetic cluster, carried *bla_NDM-7_*.

Among extended-spectrum β-lactamase genes, *bla_CTX-M-15_* was detected in 20 isolates (80%), being absent only from the five KPC-3-carrying ST307 isolates, and *bla_CTX-M-14_* in 14 (56%). Chromosomal SHV variants followed the clonal distribution. *bla_SHV-11_* was found in ST147, ST17 and ST437 isolates, *bla_SHV-28_* in ST307 isolates, and *bla_SHV-1_* in S12 (ST16, singleton) and S16 (ST101, singleton).

16S rRNA methyltransferase genes were detected in 13 isolates (52%), *rmtF1* in 12 ST147 isolates and *rmtC* in S16 (ST101, singleton). All 13 had amikacin MICs of >256 mg/L. Of the isolates lacking these genes, only S13 (ST307, singleton) was amikacin-resistant and the remaining 11 were susceptible.

The quinolone resistance-determining region (QRDR) alterations *gyrA* S83I and *parC* S80I co-occurred in all ST147, ST307 and ST437 isolates (n = 20). S12 (ST16, singleton) instead carried *gyrA* S83F/D87N and *parC* E84K, and S16 (ST101, singleton) carried *gyrA* S83Y/D87N and *parC* S80I. The ST17 isolates lacked these QRDR alterations but carried *qnrB1*. Among plasmid-mediated quinolone resistance genes, *qnrS1* was found in 15 isolates and *qnrB1* in 13.

**Figure 4:**
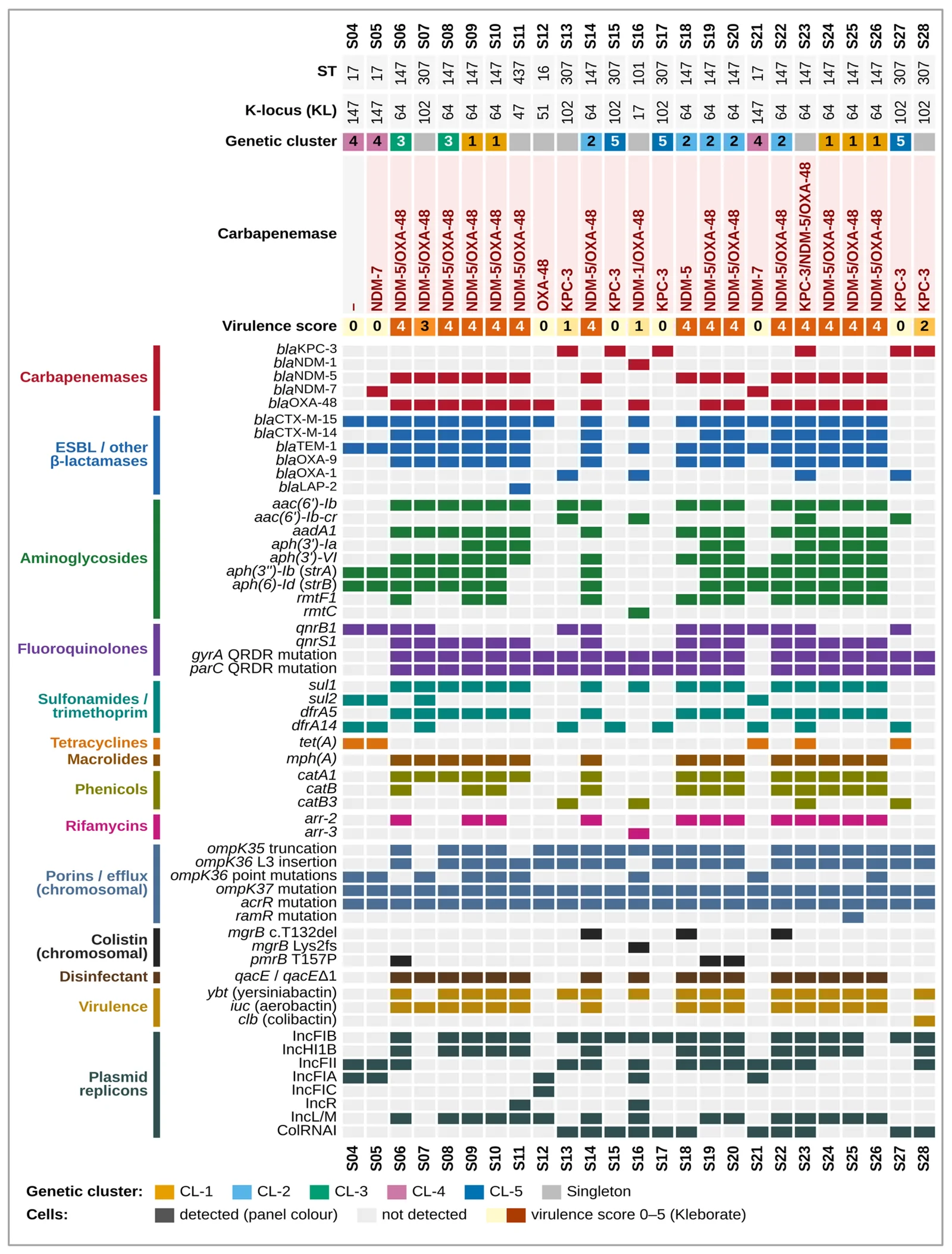
Distribution of selected resistance and virulence markers and plasmid replicons in carbapenem-resistant *Klebsiella pneumoniae* isolates. Columns represent isolates and rows represent markers grouped by antimicrobial class or function. The upper section shows the sequence type (ST), capsular locus (K-locus), genetic cluster, carbapenemase genes and Kleborate virulence score (0–5) of each isolate. In the genetic cluster row, numbers 1–5 denote genetic clusters CL-1 to CL-5, and grey cells denote isolates not assigned to any genetic cluster (singletons). Genetic clusters were defined using a threshold of ≤20 allelic differences based on 2335-locus allele profiles. In the marker section, coloured cells indicate that the corresponding marker was detected, and light grey cells indicate that it was not detected. *ompK35* truncation comprised alterations leading to a frameshift or a premature stop codon. *mgrB* c.T132del denotes the call reported by Kleborate, which in the assemblies of S14, S18 and S22 corresponded to an 8162-bp insertion within *mgrB* at codon 45. *mgrB* Lys2fs denotes a single-adenine deletion within a poly-A tract. Both *mgrB* alterations were taken from polished assemblies without read-level validation. *rmpA* and *rmpA2* were not detected as complete genes in any isolate and are not shown. The Kleborate virulence score was not interpreted as a phenotypic hypervirulence classification.

Other selected determinants were *sul1* (n = 16), *sul2* (n = 4), *dfrA5* (n = 15), *dfrA14* (n = 9), *dfrA50* (n = 11), *tet*(A) (n = 5) and *mph*(*A*) (n = 15) (Supplementary Tables S3 and S10). The number of acquired resistance genes reported by Kleborate ranged from 1 to 26 (median 16), with the highest in S23 (ST147, singleton). The resistance score was 2 in 20 isolates, 3 in four and 1 in S04 (Table 2, Figure 4).

### 3.6. Carbapenemase-Carrying Genetic Elements

**Figure 5:**
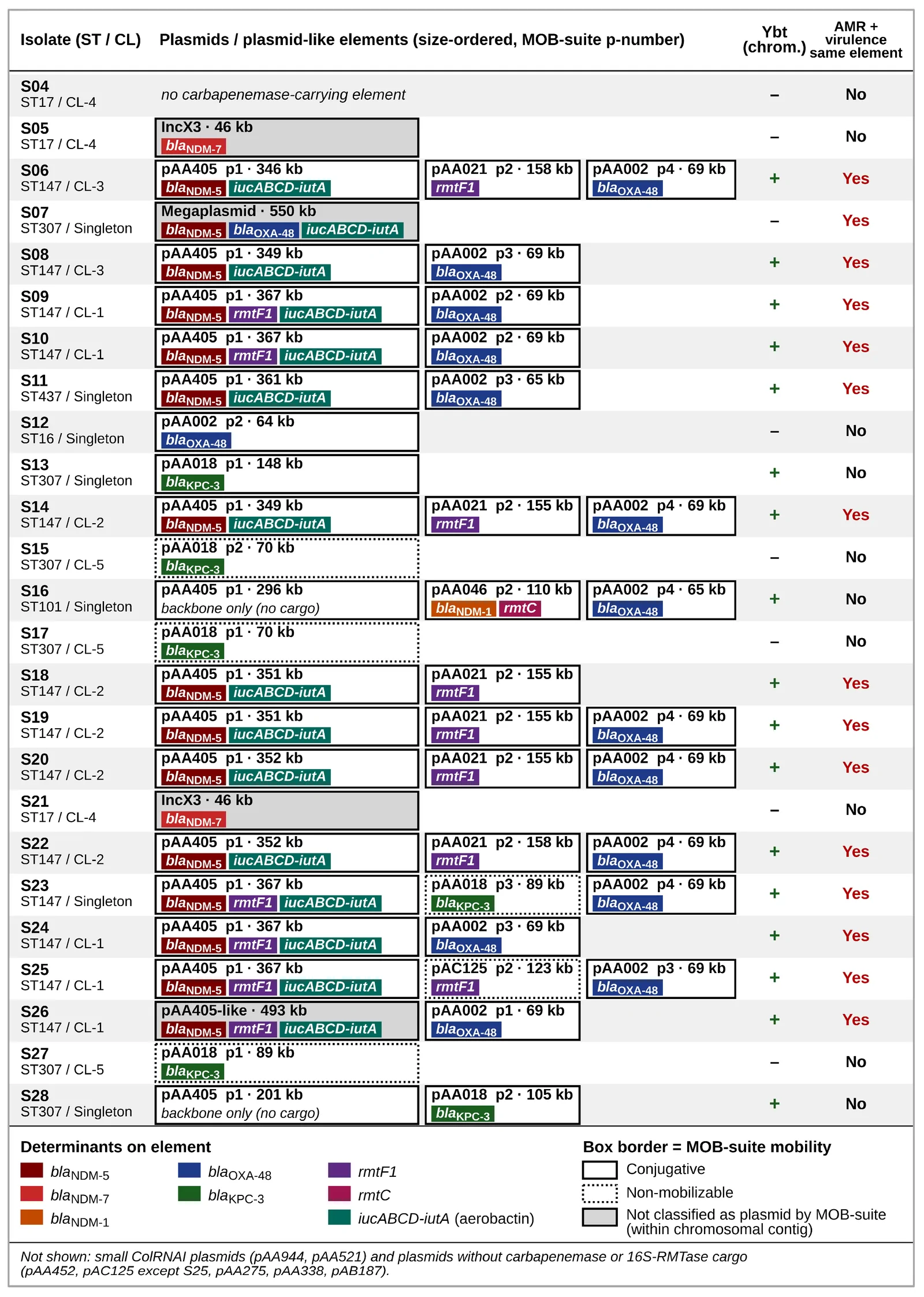
Selected carbapenemase- and 16S rRNA methyltransferase (16S-RMTase)-carrying genetic elements and associated resistance and virulence determinants in 25 carbapenem-resistant *Klebsiella pneumoniae* isolates. Each box represents one element with its MOB-suite cluster identifier (pAA or pAC), plasmid number and size. Box borders indicate the MOB-suite-predicted mobility class. Grey-filled boxes indicate elements that MOB-suite did not classify as plasmids (within chromosomal contigs). Coloured tags indicate selected determinants recorded on the element. The yersiniabactin (Ybt) column indicates the presence of the chromosomal yersiniabactin locus in the isolate. The last column indicates whether *blaNDM-5* and the complete aerobactin operon (*iucABCD-iutA*) were recorded on the same element. The figure presents selected gene–element matches and is not a complete plasmid inventory.

The isolate-level distribution, replicon content and MOB-suite mobility classes of the carbapenemase-carrying genetic elements are presented in Table 3. All carbapenemase-carrying elements, including the chromosome-associated elements of S05, S07, S21 and S26, were located on circular contigs in the final assemblies. The only open replicon in the dataset was the chromosome of S06, which did not carry a carbapenemase gene. *bla_NDM-5_* was found in 13 isolates (S06, S08, S09, S10, S11, S14, S18, S19, S20, S22, S23, S24 and S25, all ST147 except S11, which is ST437) on pAA405-family plasmids with an IncFIB+IncHI1B replicon. These plasmids ranged in size from 346,454 to 367,493 bp and were all classified as conjugative (Figure 5). *bla_OXA-48_* was detected in 15 isolates on pAA002 plasmids of 63,589–68,942 bp with an IncL/M replicon, all of which were classified as conjugative. In addition to the ST147 isolates, pAA002 was also found in the ST437 (S11), ST16 (S12) and ST101 (S16) isolates. *bla_KPC-3_* matched pAA018-family plasmids with an IncFIB replicon in six isolates. The pAA018 plasmids of 69,799 bp in S15 and S17 (ST307, CL-5) and of 89,256 bp in S23 (ST147, singleton) and S27 (ST307, CL-5) were classified as non-mobilizable, whereas the IncFIB+IncFII pAA018 plasmids of 147,568 bp in S13 (ST307, singleton) and 105,372 bp in S28 (ST307, singleton) were classified as conjugative. In S23 (ST147, singleton), *bla_NDM-5_*, *bla_KPC-3_* and *bla_OXA-48_* were located on three separate plasmids (pAA405, pAA018 and pAA002). In S05 and S21 (ST17, CL-4), *bla_NDM-7_* was found on a 46,161-bp IncX3 element. This element was located on contig 3 of the chromosomal assembly and was not classified as a plasmid by MOB-suite. In S16 (ST101, singleton), *bla_NDM-1_* was recorded together with *rmtC* on the 109,897-bp pAA046 plasmid with an IncFIB+IncFII replicon. In S07 (ST307, singleton), *bla_NDM-5_* and *bla_OXA-48_* were located on a single 550,114-bp megaplasmid with four replicons (IncFIB, IncFIB, IncFII and IncHI1B), assembled as joined to a chromosomal contig. In S26 (ST147, CL-1), *bla_NDM-5_* was found on a 492,660-bp chromosomally integrated pAA405-like element, and no separate NDM plasmid was detected in this isolate. *bla_OXA-48_* in S26 was recorded on the 68,942-bp pAA002 plasmid. Of the 13 pAA405 plasmids, nine were assembled in GUNC-flagged genomes (S06, S08, S14, S19, S20, S22, S23, S24 and S25) and four in unflagged genomes (S09, S10, S11 and S18), and their sizes were in a similar range (346,454 to 367,493 bp and 351,373 to 367,492 bp, respectively). The chromosomally integrated pAA405-like element of S26 was assembled in a GUNC-flagged genome, whereas the chromosome-joined megaplasmid of S07 and the IncX3 element of S05 and S21 were assembled in unflagged genomes. These chromosome-associated structures are assembly-based observations and were not confirmed by an independent method. Thus, carbapenemases were found exclusively on plasmids in 20 isolates, on chromosome-associated elements in S07 (ST307, singleton) and S26 (ST147, CL-1), and on an IncX3 element not classified as a plasmid in S05 and S21 (ST17, CL-4).

### 3.7. Co-Occurrence of Resistance and Virulence Determinants and Chromosomal Alterations

*bla_NDM-5_* and the complete aerobactin operon (*iucABCD-iutA*) co-occurred in 15 isolates. These were the 13 ST147 isolates together with S07 (ST307, singleton) and S11 (ST437, singleton). On the 13 pAA405 plasmids listed in Table 3, *bla_NDM-5_* and the aerobactin operon were recorded on the same element. In S07 (ST307, singleton), both determinants were located on the chromosome-joined megaplasmid, and in S26 (ST147, CL-1) on the chromosomally integrated pAA405-like element. The S26 element was assembled in a GUNC-flagged genome, and the co-localisation on the 13 pAA405 plasmids was recorded in nine flagged and four unflagged genomes. The isolate-level distribution of the virulence loci is presented in Table 4 (Figure 4 and Figure 5).

The porin, QRDR and colistin-associated alterations together with the corresponding colistin and ciprofloxacin phenotypes are presented in Table 5. An integrated isolate-level summary of clonal background, resistance phenotype by antimicrobial class, resistance genes, chromosomal alterations, carbapenemase-carrying elements and virulence traits and CRISPR-Cas subtype is given in Supplementary Table S11. A frameshift or premature stop codon consistent with *ompK35* inactivation was recorded in 20 isolates, and the *ompK36* L3 loop insertion (D135DGD) in 20 isolates. *ompK35* R60LfsTer31 was detected in the ST147 isolates, Y197Ter in the ST307 isolates other than S07, and W79Ter in S12 (ST16, singleton). In S16 (ST101, singleton), *ompK35* G62AfsTer1 was recorded. In addition, *ompK36* Q313Ter was found in S22 (ST147, CL-2), *ramR* K104RfsTer6 in S25 (ST147, CL-1) and *nfsB* H128VfsTer6 in S28 (ST307, singleton). These variants were taken from the polished consensus assemblies and were not validated by allele fractions or read counts from alignment files, by short-read sequencing or by Sanger sequencing. Insertions and deletions, including the *ompK35* and *mgrB* frameshifts, the *ompK36* insertion, *ramR* K104RfsTer6 and *nfsB* H128VfsTer6, may be affected by Nanopore-specific errors in homopolymeric regions. All 25 isolates were ciprofloxacin-resistant (MIC >32 mg/L), and QRDR alterations were recorded in 22 of them, whereas none was detected in S04, S05 and S21 (ST17, CL-4). Colistin-associated alterations were recorded in all seven colistin-resistant isolates (MIC 4–32 mg/L) and in none of the 18 susceptible isolates (MIC 0.25–1 mg/L). *pmrB* T157P was recorded in three isolates (S06 (ST147, CL-3), S19 and S20 (ST147, CL-2)) by AMRFinderPlus only. In S14, S18 and S22 (all ST147, CL-2), Kleborate reported *mgrB* c.T132del, and inspection of the assemblies showed that this call corresponded to an 8162-bp insertion within *mgrB* at codon 45, shared by the three isolates. In S16, Kleborate reported an *mgrB* frameshift at Lys2, whereas AMRFinderPlus listed K2E as an unclassified substitution. Inspection of the assembly showed deletion of a single adenine within a poly-A tract at codons 2 and 3, producing a frameshift and a premature stop at codon 9, and this finding is consistent with both calls. These alterations are consistent with the colistin phenotype and were not tested functionally. One of these colistin-associated alterations was detected in all five members of CL-2 and in S06 within CL-3. The same alterations were not detected in the CL-1 isolates (Figure 4, Supplementary Table S7). In the colistin-susceptible isolates S11 (ST437, singleton) and S28 (ST307, singleton), assembly inspection also showed alterations in *mgrB*, namely a 1066-bp insertion sequence after codon 38 in S11 and a single-nucleotide deletion at codon 43 (p.Lys43fs) in S28. Neither isolate was resistant (MIC 1 and 0.25 mg/L, respectively), and these alterations were not classified as colistin-associated. The *mgrB* alignment metrics of the isolates examined at the assembly level are given in Supplementary Table S10. Colistin resistance was therefore concordant with the presence of a colistin-associated alteration in all 25 isolates.

### 3.8. Distribution of Virulence Determinants and Kleborate Virulence Scores

The yersiniabactin locus was detected in 17 isolates (68%). All ST147 isolates together with S11 (ST437), S13 (ST307), S16 (ST101) and S28 (ST307) were positive. In the gene screening, all 11 genes of the locus were recorded together in 15 of these 17 isolates, whereas *irp2* was not recorded in S08 (ST147, CL-3) and *irp1* was not recorded in S11 (ST437, singleton). Kleborate reported partial matches for both genes, with *irp2* covering 28% of the reference sequence in S08 and *irp1* covering 19% in S11, and labelled the yersiniabactin structure of both isolates as truncated ICE*Kp*. These partial matches were not counted as presence of the genes. The complete aerobactin operon (*iucABCD-iutA*) was detected in 15 isolates (60%). This operon was found in the 13 ST147 isolates and in S07 (ST307, singleton) and S11 (ST437, singleton). Neither Kleborate nor the separate whole-genome sequencing (WGS) gene screening detected a complete *rmpA*, *rmpA2*, *rmpC* or *rmpD* in any of the 25 isolates, and the Kleborate RmST was 0 in all isolates. Kleborate reported partial *rmpA*/*rmpA2* matches in the 15 aerobactin-carrying isolates, covering 47% of the reference sequence in eight isolates (S06, S09, S10, S14, S20, S22, S23 and S25), 55% in six (S07, S08, S11, S18, S19 and S24) and 60% in one (S26). These partial matches were not counted as presence, and the virulence description therefore rests on the aerobactin and yersiniabactin loci (Supplementary Table S4). The salmochelin *iroBCDE* operon was not detected in any isolate. The colibactin cluster was found only in S28 (ST307, singleton). The Kleborate virulence score was 4 in 14 isolates, 3 in S07, 2 in S28, 1 in S13 and S16, and 0 in seven isolates. The number of virulence genes based on the Virulence Factor Database (VFDB) ranged from 63 to 100. All ST147 isolates carried 87 genes, with the highest value found in S28 and the lowest in S12. The *fimH*, *mrkABCD* and *entB* genes were recorded in all isolates. The virulence results are presented in Supplementary Table S4 (Figure 4).

### 3.9. Pan-Genome Structure

**Figure 6:**
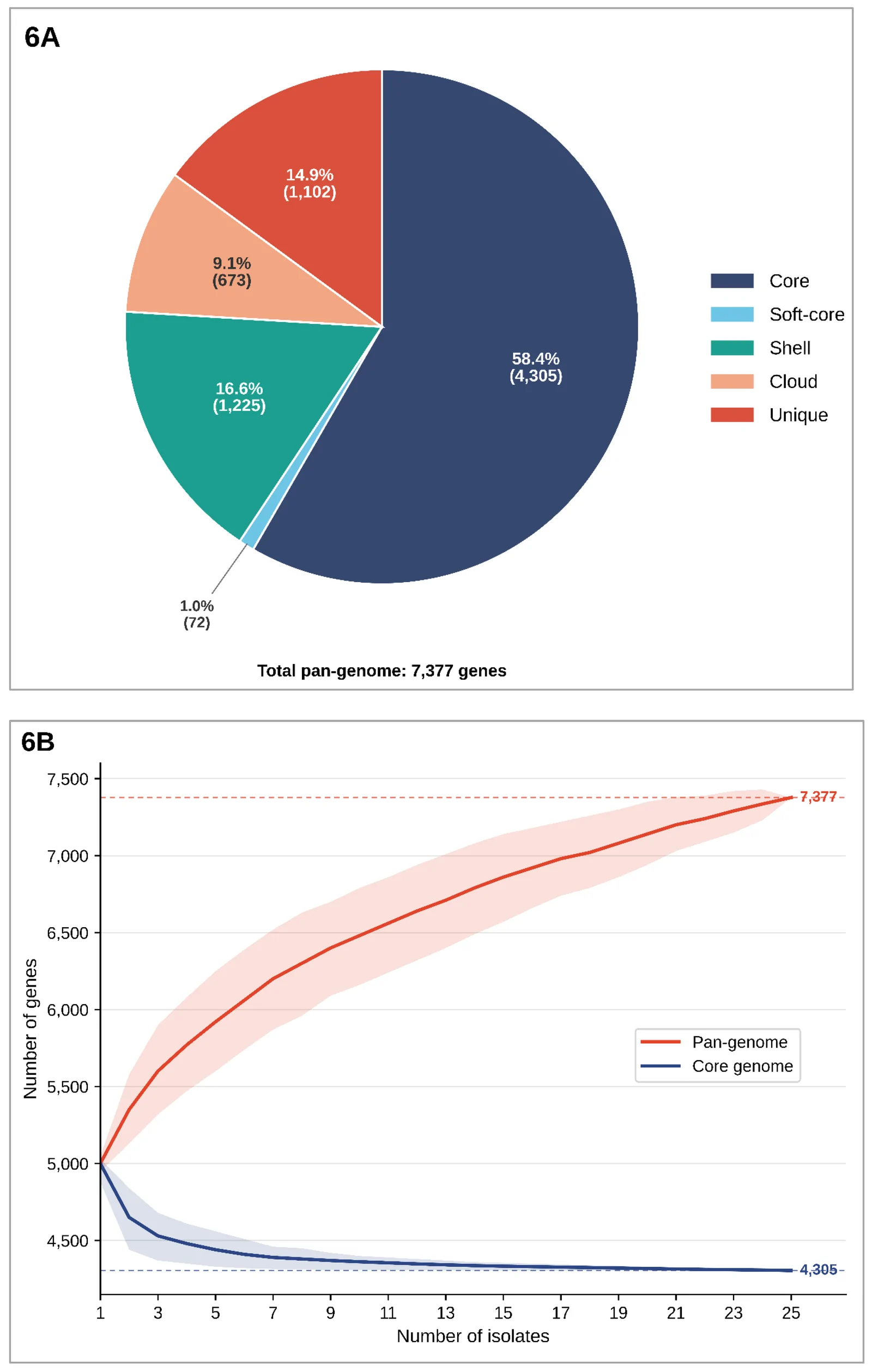

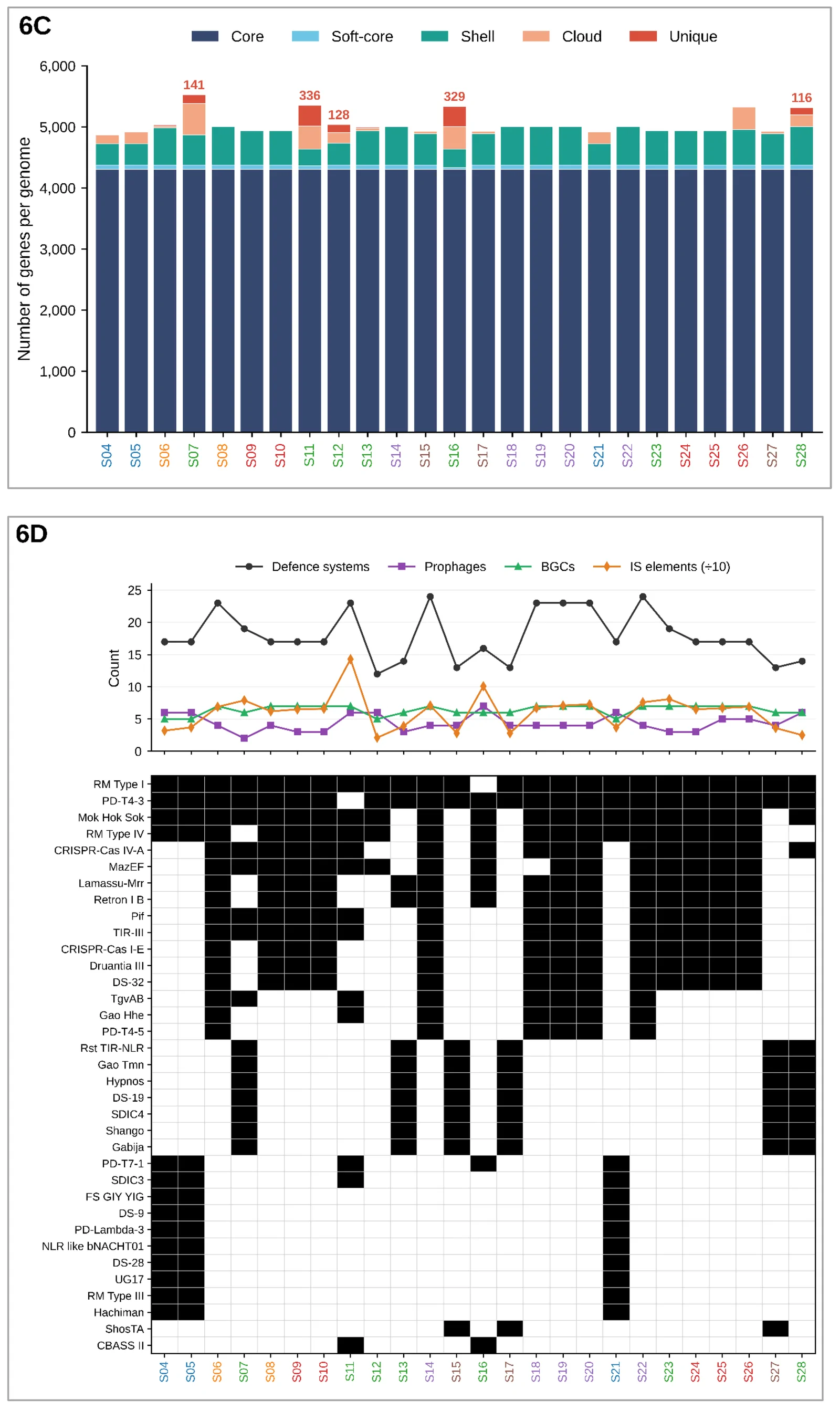
Pan-genome structure and distribution of accessory genome components across isolates. (**A**) Distribution of pan-genome gene clusters into core, soft-core, shell, cloud and strain-specific (unique) categories. (**B**) Pan-genome and core genome curves according to the number of isolates. Shaded areas indicate the variability across permutations. (**C**) Stacked bars showing the core, soft-core, shell, cloud and strain-specific gene categories at the isolate level. Numbers above the bars indicate strain-specific gene counts for isolates carrying more than 100 strain-specific genes. (**D**) Numbers of defence systems, prophages, biosynthetic gene clusters and insertion sequences, and distribution of selected defence systems. Insertion sequence counts are shown divided by 10. Black cells indicate the presence and white cells the absence of the corresponding system. In panels C and D, isolate labels are coloured by genetic cluster (CL-1 red, CL-2 purple, CL-3 orange, CL-4 blue, CL-5 brown) and singletons are shown in green.

The pan-genome analysis identified 7377 gene clusters. Of these, 4305 were classified as core, 72 as soft-core, 1225 as shell, 673 as cloud and 1102 as strain-specific gene clusters. The total number of genes per isolate, including strain-specific genes, ranged from 4869 to 5519. The highest values were recorded in S07 (5519), S11 (5351) and S16 (5335) (Figure 6A–C). The number of strain-specific genes was 336 in S11, 329 in S16, 141 in S07, 128 in S12 and 116 in S28. Eighteen strain-specific genes were recorded in S06 and S13, and 13 in S26. In the remaining 17 isolates, this number ranged from 0 to 1. The number of cloud genes was 515 in S07, 370 in S11, 365 in S16 and 362 in S26. Gene categories at the isolate level are presented in Supplementary Table S5 (Figure 6C). The pan-genome did not reach a plateau. Across 1000 random permutations of the isolate order, the 25th genome added about 44 new gene clusters on average, and the number of new clusters per added genome followed Heaps’ law, n = κN − α, with κ = 585 and α = 0.81 (95% confidence interval 0.78 to 0.83, obtained by resampling the permutations), fitted by log-log regression. An α below 1 is consistent with an open pan-genome. The estimate is based on 25 isolates, 13 of which belong to ST147, and the interval reflects the order of isolate addition only, so the result is descriptive.

### 3.10. Accessory Genome: CRISPR-Cas, Defence and Secretion Systems, Biosynthetic Gene Clusters and Prophages

A clustered regularly interspaced short palindromic repeats (CRISPR)-CRISPR-associated (Cas) system was detected in 17 isolates (68%). Types I-E and IV-A3 co-occurred in all ST147 isolates. Only type IV-A3 was recorded in S07, S11, S16 and S28. No CRISPR-Cas system was detected in the remaining eight isolates. CRISPRCasTyper identified two CRISPR arrays in each ST147 isolate and one array in each of S07, S11, S16 and S28, giving 30 arrays in total. In S28 a second array (21 repeats, 28 bp) was classified as untrusted by CRISPRCasTyper and was not counted. The I-E operon and arrays were located on the main chromosomal contig of all 13 ST147 isolates. The IV-A3 operon and array were located on the contig carrying *blaNDM-5* in 15 isolates (the pAA405-family plasmid in 13 isolates, the chromosomally integrated pAA405-like element of S26 and the megaplasmid of S07) and on contigs of 296,447 bp in S16 and 200,551 bp in S28, which carried no carbapenemase gene. The consensus repeat of the I-E arrays was identical in all ST147 isolates (GTGTTCCCCGCGCCAGCGGGGATAAACCG, 29 bp, 41 to 44 repeats). The IV-A3 consensus repeat (GTATTCCCCCCGCATGCGGGGGTTATCG, 28 bp, 14 repeats) was identical in the ST147 isolates and S07, whereas S11 (6 repeats), S16 (17 repeats) and S28 (19 repeats) carried variants of 30, 23 and 29 bp, respectively (AGTATTCCCCCCGCATGCGGGGGTTATCGG, TATTCCCCCCGTGTGCGGGGGTT and GTATTCCCCCCGCATGCGGGGGTTATCGG). The I-E arrays carried 40 to 43 spacers, with 43 distinct spacer sequences across the 13 arrays and the shorter arrays being subsets of the longest. The IV-A3 arrays of the 13 ST147 isolates and S07 carried the same 13 spacers, whereas S11, S16 and S28 carried 5, 16 and 18 spacers, respectively, none of which was shared with the ST147 group. The CRISPR-Cas subtype, repeat number and spacer number of each isolate are given in Supplementary Table S11, the array-level characteristics in Supplementary Table S12 and the spacer sequences in Supplementary Data S1. Spacer targets were not characterised. DefenseFinder identified 12–24 defence systems per isolate. The lowest value was found in S12 and the highest in S14 and S22. Restriction-modification (RM) type I, Druantia III, Lamassu-Mrr, Retron I-B, TIR-III, DS-32 and Pif systems were recorded in the ST147 isolates. Gabija, Hypnos, Shango, Gao Tmn, SDIC4 and DS-19 were found in the ST307 isolates, and Hachiman, NLR-like bNACHT01, UG17, DS-9 and DS-28 in the ST17 isolates. Cyclic oligonucleotide-based antiphage signalling system (CBASS) II was detected in S11 and S16. Anti-defence records for *apyc1* and for an *ardb/klca/acric11* entry were present in all 25 isolates. AcrIE9 was recorded in 17 isolates and AcrIE8 in six (S08 and the five CL-2 isolates S14, S18, S19, S20 and S22), and neither was recorded in the five CL-1 isolates. The total number of insertion sequence (IS) elements ranged from 20 to 144, with the lowest value recorded in S12 and the highest in S11 (Figure 6D). The type II secretion system (T2SS), type IVa pilus (T4aP), type Vb secretion system (T5bSS) and type VI secretion system subtype i (T6SSi) were detected in all isolates, and the type I secretion system (T1SS) in 24 isolates, all except S11. The protein-secretion type IV secretion system of the MPF_T type (pT4SSt) was found in 19 isolates (76%) and was not detected in S04, S07, S12, S15, S17 and S27. antiSMASH recorded 5–7 biosynthetic gene clusters per isolate. Enterobactin, pyrroloquinoline quinone (PQQ), terpene-precursor and ribosomally synthesised and post-translationally modified peptide (RiPP) clusters were found in all isolates. The colibactin cluster was detected only in S28. geNomad identified a total of 110 prophage regions. The number of prophages per isolate ranged from 2 to 7, and 91 regions passed CheckV quality control. No prophage was assigned to the complete category. *Caudoviricetes* and *Peduoviridae* were reported in the taxonomic assignments. Temperate and virulent categories were recorded in the life cycle predictions. No Comprehensive Antibiotic Resistance Database (CARD) or VFDB gene matches were detected in the prophage regions. The accessory genome components are presented in Supplementary Table S6 (Figure 6D).

## 4. Discussion

### 4.1. Principal Findings and Clinical Assessment

In our study, 25 carbapenem-resistant *K. pneumoniae* isolates obtained from a tertiary hospital between October 2023 and October 2024 were examined jointly with respect to clinical context, phenotypic susceptibility profile, clonal relationships, resistance and virulence determinants, and carbapenemase-carrying genetic elements. Six sequence types, five genetic clusters, the diversity among carbapenemase genotypes, and the co-occurrence of *blaNDM-5* with the aerobactin operon constituted the principal axes of our study.

### 4.2. Clinical and Demographic Findings

In our study the median patient age was 64 years, 80% of the patients were male, 92% of the isolates were obtained from inpatients, and 32% of the patients were in the intensive care unit at the time of sampling, with blood culture and urine (28% each) as the most frequent specimen types. The age, sex, and specimen profile of our isolates was broadly consistent with studies conducted in Türkiye. In a study of 1588 carbapenem-resistant isolates by Mermer and Çağlayan, 71% of cases were older than 60 years and a male predominance was reported [31]. In the same study, 62.5% of isolates were obtained from intensive care units and the most frequent specimen type was tracheal secretion at 43.5%. In our study the proportion of intensive care–derived isolates (32%) remained below this value, and blood culture and urine samples came to the fore. In the study by Candevir Ulu et al., 59.2% of patients were admitted to the intensive care unit and the median age was reported as 55 years [32]. In our study, the recovery of two isolates from urine samples collected during outpatient visits suggested that carbapenem resistance is not confined to a hospital-acquired setting, although the available data were insufficient to interpret these isolates as community-acquired. Mermer and Çağlayan likewise reported carbapenem resistance at 26.8% among outpatients and noted that studies based on hospital databases cannot distinguish patients recently hospitalised or with healthcare-associated exposure [31].

In our study all isolates were resistant to ertapenem and imipenem, meropenem MIC values ranged from 8 to >32 mg/L, and the upper measurement limit of the assay was exceeded in 14 isolates (56%) (Figure 1A, Supplementary Table S2). All isolates were resistant to amoxicillin-clavulanate, piperacillin-tazobactam, cefuroxime, cefoxitin, ceftazidime, ceftriaxone, cefepime, and ciprofloxacin. In the study by Mermer and Çağlayan, ciprofloxacin resistance was 91%, ceftriaxone resistance 96.7%, and piperacillin-tazobactam resistance 87.9% [31], whereas in our study resistance to these agents was 100%.

Ceftazidime-avibactam resistance was detected in 19 isolates (76%) in our study (Figure 1B). This proportion was markedly higher than the 22.5% reported by Öztaş et al. in carbapenem-resistant *K. pneumoniae* isolates [33]. Güneysu Yarar et al. also reported ceftazidime-avibactam resistance at 15% in their carbapenem-resistant isolates and detected metallo-β-lactamase production in 14% of isolates [34].

Amikacin resistance was found in 14 isolates (56%). Öztaş et al. reported amikacin resistance at 53.12%, and the result of our study was close to this value [33]. Samastı et al. reported amikacin resistance at 42.2% in carbapenem-resistant isolates [35].

Trimethoprim-sulfamethoxazole resistance was detected in 21 isolates (84%) in our study. The 72.23% reported by Öztaş et al. remained below the result of our study [33]. Samastı et al. likewise reported a high proportion and gave trimethoprim-sulfamethoxazole resistance as 82.2% [35].

Colistin resistance was detected in seven isolates (28%) (Figure 1C). Öztaş et al. reported colistin resistance at 16.74% [33], whereas in the study by Samastı et al. this proportion remained at 9% [35]. The higher proportion in our study, when considered together with the chromosomal alterations identified in our isolates, is consistent with an association between resistance and acquired mechanisms, although the mechanism was not demonstrated experimentally.

With respect to the distribution of carbapenemase genotypes, our study diverged from the general trends reported in Türkiye. Candevir Ulu et al. found an OXA-48 proportion of 74.5% and an NDM proportion of 20.4% in the 2013–2014 period [32]. Jameel et al. in Kayseri reported OXA-48 alone in 60.7% of isolates and together with NDM in 14.3%, and did not detect KPC at all [2]. In our study, by contrast, NDM-type genes came to the fore, the most frequent profile was the co-occurrence of *blaNDM-5* with *blaOXA-48*, and OXA-48 and KPC-3 were recorded at lower proportions.

This co-occurrence has also been described in international data. Ambretti et al. in northern Italy examined 45 clinical isolates co-producing NDM and OXA-48-like carbapenemases, identified 44 of them as ST147, reported that only aztreonam-avibactam susceptibility was preserved, and gave cefiderocol resistance as 42.2% [36].

### 4.3. Genomic Population Structure, Clonal Relationships and Temporal Distribution

The isolates included in our study were distributed across six sequence types (Table 1). The capsular locus was fully concordant with the sequence type within ST147 (KL64/O2α, *wzi64*), ST307 (KL102/O2β, *wzi173*), and ST17 (KL147/O2β, *wzi349*), and divergence was seen only in the three singleton isolates. ST147 and ST307 are regarded as high-risk clones that probably emerged in Europe in the early to mid-1990s and subsequently spread to all continents except Antarctica [37].

ST307 has emerged as a dominant clone in different geographies. In South Korea, 79.7% of 119 carbapenem-resistant isolates collected from nine regions were identified as ST307, and all isolates carried the KL102 and O1/O2v2 loci [38]. A study in South Africa also found ST307 isolates to be clonally related and reported that they carried KL102 (*wzi173*) [39]. In our study all ST307 isolates carried KL102/O2β and *wzi173*, of which S15, S17, and S27 grouped in cluster CL-5 while S07, S13, and S28 were classified as singletons. Within the same sequence type, the five KPC-3-carrying isolates and S07, which carried the co-occurrence of NDM-5 and OXA-48, were found together, showing that the same ST and K/O type does not imply identical accessory gene content.

The population structure was presented on the basis of a shared 2335-locus allele profile. Five genetic clusters were defined at a threshold of ≤20 allelic differences, 18 isolates (72%) fell within clusters, and seven isolates remained singletons (Table 1, Figure 2A–C, Supplementary Figures S1 and S2).

The largest within-cluster distances were 20 alleles in CL-2 and 17 alleles in CL-3 (S06 and S08), both close to the threshold, and no pairwise distance fell between 21 and 24 alleles. The nearest neighbour of S23 was S09, with 144 allelic differences between them, and S23 was therefore classified as a singleton.

The allelic difference threshold used to define clusters has varied among studies. In a study of four hospitals in Berlin, 57 NDM-1-carrying isolates were examined by cgMLST and three outbreak clusters were defined using a threshold of 15 allelic differences [40]. In the South Korean study, a threshold of 12 allelic differences was used [38]. In our study the threshold of ≤20 allelic differences was fixed before the allelic distance matrix was examined and was not changed afterwards. CL-4 and CL-5 were unchanged from ≤6 to ≤20 allelic differences, whereas the membership of CL-1, CL-2, and CL-3 depended on the threshold (Supplementary Table S8).

When the temporal distribution of the clusters was examined, Of the 18 clustered isolates, six came from intensive care units and 12 from wards or outpatient clinics. S09 and S10 were obtained in different months from outpatient urine and a ward blood culture, and S19 and S20 were obtained within the same month from oncology and rheumatology wards from different specimen types, yet showed zero allelic differences. CL-2 encompassed outpatient, ward, and intensive care sources together, and CL-3 covered an oncology ward and an anaesthesia intensive care unit (Supplementary Table S1).

This distribution showed that genetic proximity alone does not directly indicate patient-to-patient transmission. In a study of three hospitals in Italy, 19 ST147 isolates producing NDM-14 showed 8–91 core SNP differences and the most recent common ancestor was dated to June 2024. The main chain, encompassing 11 of 18 cases, spread among wards and facilities, while five cases remained epidemiologically unrelated [41]. In our study, the time intervals in Figure 3 were not regarded as evidence of uninterrupted circulation or direct transmission.

The clonal distribution reported at the national level was also taken into account. Across Türkiye, a polyclonal population and inter-hospital spread were reported in isolates from intensive care patients in six geographical regions, with ST2096 and CC14 found to be dominant [42]. In contrast, the national study covering 19 tertiary hospitals reported that ST2096 was the dominant clone in 2018–2019 whereas ST147 became the dominant lineage after 2022, characterised by the co-occurrence of *blaNDM-5* with *blaOXA-48* and the KL64 capsule type [3]. In our study ST2096 was not detected at all, ST147 was the dominant sequence type, and all such isolates carried KL64/O2α and *wzi64*. In Italy, 44 of 45 clinical isolates co-producing NDM and OXA-48-like carbapenemases being ST147 also supported the regional spread capacity of this clone [36].

### 4.4. Antimicrobial Resistance and Genotype–Phenotype Concordance

At least one carbapenemase gene was found in 24 isolates (96%) and more than one in 15 isolates (60%). NDM-type genes were recorded in 18 isolates, and NDM-5/OXA-48 was the most frequent profile (13 isolates). ST147 isolates carrying *blaNDM-5* as the sole carbapenemase gene [43] or *blaNDM-1* together with *blaOXA-48* [44] have also been reported from other countries. *blaCTX-M-15* was reported in 20 isolates and *blaCTX-M-14* in 14, and *blaCTX-M-15* was absent from the five KPC-3-carrying ST307 isolates (Table 2, Supplementary Table S3).

Porin alterations were evaluated together with the carbapenemase content of the isolates. In our study an alteration consistent with *ompK35* inactivation was found in 20 isolates and an *ompK36* L3 loop insertion in 20 isolates, and the variants differed among sequence types (Table 5, Figure 4). OmpK35/36 deletions do not by themselves lead to carbapenem resistance, but in the presence of *blaKPC-2* they markedly raise resistance to certain β-lactams including ceftazidime-avibactam [45], and the Gly115-Asp116 insertion in the L3 loop reduces the pore diameter, raising the meropenem MIC in the presence of KPC-2 and carrying a fitness cost in a murine pneumonia model [46]. S04 (ST17, CL-4), the only isolate in which no carbapenemase gene was detected, had a meropenem MIC of 24 mg/L and carried *blaCTX-M-15* together with *ompK36*, *ompK37*, and *acrR* alterations. It belongs to the same cluster as the NDM-7-carrying S05 and S21, and its resistance was not attributed to a single alteration. Because porin expression and function were not tested, the porin alterations are regarded as consistent with, but not proof of, a contribution to the carbapenem-resistant phenotype.

The ceftazidime-avibactam MIC values of the resistant isolates ranged from 32 to >256 mg/L (Figure 1B, Supplementary Table S2). All 18 NDM-carrying isolates were resistant (Table 2), which is consistent with the lack of avibactam inhibition of metallo-β-lactamases. The MIC value itself may additionally be influenced by other mechanisms that were not examined. The only resistant isolate without an NDM gene was S15 (ST307, CL-5, KPC-3 as the only carbapenemase, >256 mg/L), whereas S17 and S27 of the same cluster (CL-5) were susceptible (0.75 and 1 mg/L). S15 and S17 carried a pAA018 element of the same size (69,799 bp, Table 3), which did not account for this divergence. *blaKPC-3* copy number, promoter variants, and OmpK36 variant differences were not tested in our study. The difference is therefore an important limitation and is reported as an unexplained observation, not as evidence of a broader genotype–phenotype principle. Vaiana et al. detected a truncated OmpK35, alone or together with a heavily mutated OmpK36, in all 16 KPC-producing blood isolates resistant to ceftazidime-avibactam, and reported the most frequent alteration to be an L3 loop insertion [47]. In our study, the *ompK36* L3 loop insertion being present in 20 isolates is consistent with this distribution, although porin expression was not measured and no mechanistic link is drawn for individual isolates.

The amikacin MIC was >256 mg/L in 13 of the 14 resistant isolates and 16 mg/L in S13 (ST307), whereas the susceptible isolates ranged from 0.38 to 8 mg/L (Supplementary Table S2). *rmtF1* was found in 12 ST147 isolates and *rmtC* in S16, and in all 13 of these carriers the amikacin MIC was >256 mg/L (Table 2). S08 (ST147, CL-3) remained susceptible (8 mg/L) without a methyltransferase, whereas S13 was resistant without a detected methyltransferase, and the basis of this resistance was not determined (Supplementary Table S3). In Spain, a methyltransferase was detected in 24 (5.1%) of 468 carbapenemase-producing isolates collected from 32 hospitals, of which 21 were RmtF, two ArmA, and one RmtC. All showed high-level resistance to all aminoglycosides including plazomicin, and the isolates were associated with the ST147, ST101, and ST395 clones [48]. In our study, *rmtF1* being detected in 12 ST147 isolates and the amikacin MIC being >256 mg/L in all of these isolates is consistent with this distribution.

The colistin MIC values of the seven resistant isolates were 4 to 8 mg/L in six isolates and 32 mg/L in S16 (ST101), whereas the susceptible isolates ranged from 0.25 to 1 mg/L (Figure 1C, Table 5). *pmrB* T157P was found in three ST147 isolates (S06 of CL-3, S19 and S20 of CL-2), an 8162-bp *mgrB* insertion (reported by Kleborate as c.T132del) in S14, S18, and S22 (ST147, CL-2), and *mgrB* Lys2fs in S16, and these alterations were not detected in the susceptible isolates (Table 5, Figure 4). Genotype-based prediction of antimicrobial susceptibility in *K. pneumoniae* has shown an overall agreement of 95.6% with phenotypic testing in an earlier cgMLST workflow study [25], and the *mgrB* and *pmrB* findings were therefore read together with the colistin MIC values. Insertional inactivation of *mgrB* has been described as a common mechanism of colistin resistance in KPC-producing *K. pneumoniae* [49], and in another collection complementation with wild-type *mgrB* restored colistin susceptibility in isolates with IS insertions and nonsense mutations, whereas the *pmrB* substitutions detected in that collection were not shown to confer resistance [50]. The *pmrB* T157P substitution has also been reported among colistin-resistant carbapenem-resistant *K. pneumoniae* [51].

Colistin resistance affected all CL-2 members, in which different alterations were found (*pmrB* T157P in S19/S20 and the *mgrB* insertion in S14/S18/S22), was absent in CL-1, and divided CL-3 (S06 resistant and S08 susceptible, Table 5). In a study of 165 carbapenem-resistant isolates in Thailand, colistin resistance was 28.5%, *mgrB* alterations were detected in 85.1% of resistant isolates, *mgrB* inactivation was shown to occur predominantly through insertion sequences, and the *pmrB* T157P alteration was found together with increased expression of the *pmrCAB* operon [51]. In our study the *pmrB* T157P alteration, detected in three isolates, is consistent with a recognised colistin resistance mechanism but was not regarded as causal evidence on its own. The *mgrB* insertion in S11 and the frameshift in S28 were not accompanied by resistance, so the presence of an *mgrB* sequence alteration was not sufficient for the resistant phenotype in our isolates, although their functional effects were not tested. These findings are therefore reported as genotypic associations.

The ciprofloxacin MIC value was >32 mg/L in all isolates, so the phenotype did not discriminate among genotypes. The *gyrA* S83I/*parC* S80I combination was recorded in all ST147, ST307, and ST437 isolates, a total of 20 isolates, *gyrA* S83F+D87N/*parC* E84K in S12 (ST16), and *gyrA* S83Y+D87N/*parC* S80I in S16 (ST101, Table 5). The same *gyrA* and *parC* substitutions have been reported to be shared by ST147 and ST307 genomes in earlier genomic studies [37]. The specified QRDR alterations were not found in ST17 isolates, and *qnrB1* was recorded, so the high MIC value in these isolates was not explained by *qnrB1* alone. The trimethoprim-sulfamethoxazole MIC value was >160 mg/L in 19 isolates and 40 mg/L in S12 (ST16) and S15 (ST307), and these 21 isolates were categorised as resistant. The ≤20 mg/L value reported in S13, S17, S27, and S28 corresponded to ≤1 mg/L for the trimethoprim component and could not be interpreted because of the EUCAST breakpoint of 0.5 mg/L (Supplementary Table S2). In S25 (ST147, CL-1) a K104RfsTer6 alteration in *ramR* and in S28 (ST307) an H128VfsTer6 alteration in *nfsB* were detected. Because the antibiotics associated with these alterations were not included in the susceptibility panel, their phenotypic counterparts were not assessed (Supplementary Table S3).

The relationship between resistance phenotype and genotype was established at the isolate level, and the differences among the resistance profiles of isolates within the same genetic cluster were preserved. The associations were strongest where a recognised mechanism is available, as for NDM and ceftazidime-avibactam or 16S rRNA methyltransferases and amikacin, and weaker for carbapenem and colistin MIC variation, and none was tested functionally. The determinants therefore had to be assessed together with the carrier structures and the virulence determinants.

### 4.5. Carbapenemase-Carrying Genetic Elements and Resistance–Virulence Co-Occurrence

In our study the isolate-level distribution, replicon content, and MOB-suite mobility classes of the carbapenemase-carrying genetic elements are presented in Table 3, and *blaNDM-5* was matched to the pAA405 family in S06, S08, S09, S10, S11, S14, S18, S19, S20, S22, S23, S24, and S25 (12 ST147 isolates and S11 of ST437). The presence of hybrid plasmids harbouring both resistance and virulence determinants has been shown in high-risk clones carrying *blaNDM-5* [52], and it has been reported that IncFIB(pNDM-Mar)/IncHI1B(pNDM-MAR) hybrid resistance–virulence elements are found in a high proportion of ST147 isolates and that *blaNDM-5-carrying* examples show high similarity to elements described in ST383 [53]. In our study the co-occurrence of *blaNDM-5* with the complete aerobactin operon was detected in 15 isolates (13 ST147 plus S07 and S11), and in this group S18 (ST147, CL-2) carried *blaNDM-5* as its only carbapenemase gene. A study of 205 isolates comprising 62 sequence types in Huzhou likewise reported that the co-occurrence of resistance and virulence determinants was common [54], which is consistent with the presence of this combination in S07 (ST307) and S11 (ST437) in addition to ST147. In the 13 pAA405 matches listed in Table 3, *blaNDM-5* and the aerobactin operon were recorded on the same element. In S11 (ST437) *blaNDM-5* was located on a conjugative pAA405 plasmid, and in S07 (ST307) on a megaplasmid co-assembled with a chromosomal contig together with the aerobactin operon. S23 (ST147, singleton), the only isolate in which *blaNDM-5*, *blaKPC-3*, and *blaOXA-48* co-occurred, was represented by matches to three separate plasmids (pAA405, pAA018, and pAA002) (Table 3, Figure 5).

*blaOXA-48* was matched in 15 isolates to IncL/M-replicon pAA002 plasmids of 63,589–68,942 bp, all classified as conjugative, and pAA002 was detected in ST437 (S11), ST16 (S12), and ST101 (S16) isolates in addition to ST147 (Table 3). *blaKPC-3* was matched in six carrier isolates (five ST307 isolates and S23 of ST147) to the IncFIB-replicon pAA018 family, with plasmids of 69,799 bp in S15 and S17, 89,256 bp in S23 and S27, 147,568 bp in S13, and 105,372 bp in S28. The mobility class of the first two size groups was reported as non-mobilizable and that of the latter two IncFIB+IncFII-replicon groups as conjugative. The diversity of KPC-carrying plasmids in size, replicon, and mobility showed that different plasmid backbones can be maintained within the same species [55]. In our study, S15/S17 and S23/S27 sharing the same size groups and the differences in S13/S28 are consistent with this diversity.

In S05 and S21 (ST17, CL-4), *blaNDM-7* was matched to a 46,161 bp IncX3 element. This element was located on contig 3 of the chromosomal assembly and, because it was not classified as a plasmid by MOB-suite, no mobility class was given in Table 3. It has been reported that *blaNDM-7* is carried on IncX3-type conjugative plasmids and can be transferred by conjugation [56], and that the same element family has also been described in a strain isolated from a paediatric patient within a conserved genetic context [57]. In our study, S05 and S21 being in the same genetic cluster (CL-4) and carrying an IncX3 element of the same size were assessed together with this spread capacity. In S16 (ST101), *blaNDM-1* was matched to a 109,897 bp IncFIB+IncFII-replicon pAA046 plasmid, and *rmtC* was also recorded on the same plasmid. In a previously described *K. pneumoniae* strain carrying *blaNDM-1*, the aminoglycoside resistance genes *aadA2* and *rmtB* were located in the multidrug-resistance region of a separate plasmid [58].

Two isolates in which the carbapenemase genes were located on chromosome-associated elements were assessed separately. In S07 (ST307), *blaNDM-5* and *blaOXA-48* were located on a single 550,114 bp megaplasmid with four replicons (IncFIB, IncFIB, IncFII, IncHI1B) co-assembled with a chromosomal contig. In S26 (ST147, CL-1), *blaNDM-5* was detected on a 492,660 bp chromosomally integrated pAA405-like element not classified as a plasmid by MOB-suite, whereas *blaOXA-48* was recorded on a pAA002 plasmid of the same size (68,942 bp) as in the other ST147 isolates. S26 being within CL-1 and *blaNDM-5* being located on conjugative pAA405 plasmids in S09, S10, S24, and S25 in the same cluster showed that the same carbapenemase can be carried in different genomic contexts within the same genetic cluster. In these two isolates, chromosomal integration was presented at the level of the software output, taking into account the limits of long-read-only assembly. Chromosomal placement of carbapenemase genes has been documented in long-read assemblies from other collections, including *blaNDM-5* in the chromosome of an ST147 isolate [53] and *blaOXA-48*-like, *blaKPC* and *blaVIM* in a smaller number of hybrid assemblies of European *K. pneumoniae* isolates [59].

The matches of pAA002 across different STs and the different carbapenemase contents within the same ST were assessed at separate levels, and clonal spread and element sharing were not used interchangeably. The presence of genes on the same element was not interpreted as implying shared expression, co-transfer, or increased clinical virulence (Table 3, Supplementary Table S3). Distinct routes of carbapenemase gene spread have been described in European *K. pneumoniae* collections, with a single plasmid family carrying *blaOXA-48*-like across many lineages and diverse plasmids carrying *blaNDM* across lineages [59], which is consistent with assessing clonal spread and element sharing separately.

### 4.6. Diversity of the Virulome and Interpretation of Virulence Markers

In our study the yersiniabactin locus was detected in 17 isolates (68%), and all ST147 isolates together with S11, S13, S16, and S28 were positive. In a surveillance study of 282 carbapenem-resistant isolates in China, yersiniabactin was detected in more than 96% of isolates and was assessed among the core virulence genes [60]. The 68% obtained in our study remained below this 96% level, yet the locus was preserved in all 13 ST147 isolates, which indicates that the distribution of yersiniabactin followed the clonal background of our collection and not a uniform presence. Within the locus, *irp2* was not recorded in S08 and *irp1* in S11 (Supplementary Table S4). Kleborate reported partial matches for both genes and labelled the yersiniabactin structure of these two isolates as truncated ICE*Kp* (Supplementary Table S4). Whether these records reflect true sequence loss or incomplete assembly of the region was not determined, and they were not designated as experimentally demonstrated gene deletions.

The complete aerobactin operon (*iucABCD-iutA*) was detected in 15 isolates (60%) in our study, and these isolates consisted of 13 ST147 isolates plus S07 and S11. S07 (ST307) was assessed separately for its aerobactin-positive and yersiniabactin-negative profile (Supplementary Table S4, Table 4), and the relation of aerobactin to the carbapenemase-carrying elements is considered in Section 4.5. In a study of 112 isolates in Japan, aerobactin was a significant diagnostic predictor for distinguishing hypervirulent isolates from classical isolates (OR 4.59, 95% CI 1.22–17.2, positive likelihood ratio 3.49) and was highlighted as the siderophore contributing most strongly to hypervirulence among yersiniabactin, salmochelin, and enterobactin [61].

The aerobactin proportion in our collection (60%) was not interpreted as a direct application of the diagnostic criteria reported by Matono et al. and was not used as an indicator of hypervirulence prevalence [61]. Aerobactin was present in two isolates outside ST147 (S07 and S11), and its distribution therefore followed the dominant clone but was not confined to it.

The stability of the *rmpA* and *rmpA2* markers is debated in the literature. It has been shown that virulence plasmid–carrying isolates in high-risk clones can carry the *rmpA* and *rmpA2* genes in a non-functional form as a result of frameshift and premature stop codons, whereas aerobactin synthesis remains functional [62]. The same study reported that the IucA and IutA proteins are more stable than RmpA and that aerobactin stands out as a marker of hypervirulence [62]. In our study no complete *rmpA* or *rmpA2* was detected by either calling approach, and the partial matches reported by Kleborate in the aerobactin-carrying isolates were not counted as presence (Supplementary Table S4). These partial matches covered 47% to 60% of the reference sequence, which is below the 80% coverage threshold of the VFDB screening and is consistent with their being reported only by Kleborate. Whether these partial matches reflect non-functional copies of the kind described above or differences between the reference sequence and the assemblies was not determined, and the virulence description was therefore limited to the aerobactin and yersiniabactin loci.

The Kleborate virulence score is calculated between 0 and 5 according to the presence of the yersiniabactin, colibactin, and aerobactin loci [23]. The score of 4 in 14 isolates (the 13 ST147 isolates and S11) corresponded to the co-occurrence of aerobactin and yersiniabactin without colibactin (Table 2, Supplementary Table S4), so the score largely reflected the clonal background. The VFDB-based virulence gene count and the locus-based score are different measures, which explains why S28 (ST307) had the highest gene count (100) but not the highest score. *fimH*, *mrkABCD*, and *entB* genes were also recorded in isolates with a score of 0, so these genes did not discriminate among isolates. It has been shown that the presence of all five biomarkers (*iucA*, *iroB*, *peg-344*, *rmpA*, and *rmpA2*) distinguishes hypervirulent isolates with 94% sensitivity and 94% specificity, whereas gene presence alone is not sufficient for a diagnosis of hypervirulence [63]. Similarly, it has been reported that the K1 genotype, *rmpA*, and aerobactin are significant diagnostic predictors and that the capsule serotype and the string test alone are not sufficient [61]. In our study, virulence gene–carrying isolates were not assessed for phenotypic hypervirulence, and none of the capsular loci in Table 1 corresponds to K1. The virulence results are therefore reported as genotypic markers, and no inference was drawn for virulence potential or clinical outcome.

### 4.7. Genome Features and Pan-Genome Diversity

In our study, the chromosome was circular in 24 isolates and linear in S06, and the total genome size (5.41–6.12 Mb) exceeded the chromosome size (5.26–5.63 Mb) by the non-chromosomal contigs of each assembly (Supplementary Table S5). In a study of 30 carbapenem-resistant isolates in Gansu province, China, genome sizes ranged from 5.2 to 5.8 Mb and a mean of 5596 coding sequences per isolate was recorded [64]. In a pan-genome study of 198 clinical isolates in Tanzania, a mean assembly length of 5.57 Mbp and a mean GC content of 56.78% were reported [65]. The chromosome sizes in our study fell within the range reported in the Gansu study, whereas total genome sizes of up to 6.12 Mb exceeded it and the mean assembly length in the Tanzanian study, a difference that may partly reflect the plasmid content of the long-read assemblies. The GC content of 56–57% was close to the mean reported in the Tanzanian study.

Assembly quality was evaluated with CheckM2, BUSCO, GTDB-Tk, and GUNC. CheckM2 and BUSCO indicated complete genomes with low estimated contamination, and GTDB-Tk ANI values were similar in all isolates (Supplementary Table S5), whereas GUNC reported an above-threshold clade separation score (CSS) at the family level in 10 isolates, all of which were ST147 (Supplementary Tables S5 and S9). The approach of evaluating genome quality with multiple independent tools was applied in a pan-genome study of 350 genomes through the joint use of BUSCO and contig-level filters [66] and in a study of 656 human gut–derived genomes through the use, after a CheckM filter of >90% completeness and <5% contamination, of joint GUNC thresholds of a clade separation score of 0.45 and a contamination proportion of 0.05 [67]. In our study, the contamination portion of the flagged genomes (0.02) was below the proportion of 0.05 applied jointly with the CSS in the 656-genome study, and the CheckM2 contamination estimate did not separate flagged from unflagged genomes. The flag also did not follow cgMLST relatedness, since three ST147 isolates were not flagged (Section 3.3). The origin of the signal was not determined, and the CSS values were therefore neither accepted as evidence of contamination or chimeric assembly nor excluded as a source of assembly error. Gene-content and element-level observations in the flagged genomes are accordingly presented as assembly-based descriptions.

In the pan-genome analysis of our study, the core category comprised 58.4% of the 7377 gene clusters, the shell category 16.6%, the cloud category 9.1%, and strain-specific clusters 14.9% (Figure 6A). In an analysis of 310 clinical genomes, the proportion of mobile element–associated genes in the accessory genome was 14.6%, whereas it remained at 0.1% in the core genome [68]. This proportion was not calculated in our study, and the insertion sequence content is considered in Section 4.8.2. In the Tanzanian isolates, 30,992 gene families were distributed as persistent (13.6%), shell (11.2%), and cloud (75.2%), and a U-shaped gene frequency curve was assessed as an indicator of an open pan-genome structure [65]. In a study in China assessing 40 carbapenem-resistant and 42 carbapenem-susceptible isolates together with 2460 publicly available genomes, an open pan-genome feature was shown in both groups and cloud genes were reported as the largest category [69]. In our study, the cloud proportion was lower than in the Tanzanian isolates, but category proportions depend on the number and diversity of the genomes analysed. Because 13 of the 25 isolates belong to ST147, clusters shared within this clone were classified as shell, and the cloud proportion therefore reflects the single-centre and clonal composition of the collection. The Heaps’ law exponent below 1 (Section 3.9, Figure 6B) was in the same direction as the open pan-genome structures reported in the Tanzanian and Chinese collections. It has been shown that the choice of threshold can change the interpretation of the pan-genome curve, an open pan-genome being observed with all accessory genes in an analysis of 656 genomes while this trend was lost with only genes present in more than 1% of genomes [67]. The confidence interval of the estimate reflects only the order of isolate addition. The pan-genome was therefore described as consistent with an open structure, and no inference was drawn about the asymptotic size of the species pan-genome.

At the isolate level, the strain-specific gene clusters were concentrated in five isolates, S07 (ST307), S11 (ST437), S12 (ST16), S16 (ST101), and S28 (ST307), all of which were singletons in the cgMLST analysis, and the counts of all isolates summed to the 1102 strain-specific clusters of the pan-genome analysis (Supplementary Table S5, Figure 6C). The strain-specific category is relative to the collection, being defined by presence in a single genome, and a lineage represented by one isolate, as ST437, ST101, and ST16 were here, would be expected to contribute such clusters more readily than a lineage represented by several isolates. That allelic proximity and accessory gene content provide information at different scales was seen in the S19 and S20 pair (ST147, CL-2). Despite zero allelic differences, these two isolates had total gene counts of 5008 and 5011 (Supplementary Table S5). In CL-1, S26 (5332 genes) diverged from S24 and S25 (4938 and 4942), and because S26 is a GUNC-flagged genome this divergence is reported as an assembly-based observation. The extent of within-cluster divergence also differed among clusters, with S04 carrying 4869 genes against 4922 in S05 and S21 in CL-4, a difference of 53 genes that is compatible in size with the 46,161 bp IncX3 element present in S05 and S21 and absent from S04 (Table 3), whereas the CL-5 isolates S15, S17, and S27 differed by at most one gene (4924–4925). That S23 (ST147, singleton) had no strain-specific cluster despite carrying 26 acquired resistance genes indicated that resistance gene content and strain-specific gene content are different measures. That accessory genome content can diverge in relation to the capsule type was shown by ST11-KL25 and ST11-KL64 strains sharing a conserved core genome while overlapping only 6.28% with non-ST11 strains [64]. In the Tanzanian study, 90 different sequence types were detected and isolates sharing the same K-locus and O-locus types were reported to group in the same clades in the phylogenetic tree [65]. In our study too, ST147 isolates sharing the KL64/O2α type differed from one another in accessory gene content, as in CL-1 and CL-2 (Table 1).

### 4.8. Defence Systems and Other Accessory Genome Components

#### 4.8.1. CRISPR-Cas and Defence Systems

In our study a CRISPR-Cas system was detected in 17 of 25 isolates (68%). Types I-E and IV-A3 co-occurred in all 13 ST147 isolates, whereas only IV-A3 was recorded in S07 (ST307, singleton), S11 (ST437, singleton), S16 (ST101, singleton), and S28 (ST307, singleton), and no system was detected in the other eight isolates (Supplementary Table S6). The two systems differed in genomic location. The I-E operon and arrays were on the main chromosomal contig of every ST147 isolate, whereas the IV-A3 operon and array were on the contig carrying *blaNDM-5* in 15 isolates, namely the pAA405-family plasmid in 13 isolates (12 ST147 isolates and S11), the chromosomally integrated pAA405-like element in S26 (ST147, CL-1), and the megaplasmid in S07 (Table 3). In S16 and S28, IV-A3 was on contigs of 296,447 and 200,551 bp that did not carry a carbapenemase gene. This distribution is consistent with the chromosomal location of type I systems and the plasmid origin of type IV systems [70]. The joint preservation of the two systems in a high-risk clone such as ST147 is consistent with the finding, demonstrated by Jiang et al. in conjugation experiments, that the I-E and IV-A3 systems reduce the transfer frequency of the pKpQIL plasmid at a level of *p* < 0.001 [71]. Because IV-A3 was physically linked to the NDM-5-carrying element, its presence in our isolates is a genomic observation, and an effect on the transfer of this element cannot be inferred from the sequence data.

The relationship of CRISPR-Cas systems with resistance and virulence is debated in the literature. Kadkhoda et al. reported that, in 156 carbapenem-resistant isolates, strains carrying type I-E showed a higher tendency towards *blaNDM-1* and virulence genes such as aerobactin, *rmpA*, *iutA*, *iucB*, and *ybtS* [72]. In contrast, Saedi et al. found the type I-E system in 16% of carbapenem-resistant isolates and 36% of susceptible isolates [70]. Jurić et al. in turn, detected CRISPR-Cas presence at 15.8% in 400 clinical isolates and showed that *blaOXA-48* was more frequent in negative strains and *blaNDM-1* and *blaKPC-2* in positive strains [73]. Type I-E was recorded only in ST147, so its distribution corresponded to the clonal background. The eight isolates without a CRISPR-Cas system carried NDM-7, OXA-48 or KPC-3, or no carbapenemase gene. The ST147 isolates carried the complete aerobactin operon together with I-E, which agrees in direction with the higher tendency of type I-E strains to carry aerobactin reported by Kadkhoda et al. [72]. However, I-E, IV-A3, *blaNDM-5* and aerobactin were carried together by one clone and, for the last three, by one plasmid, so CRISPR-Cas status could not be separated from lineage and plasmid background in this collection.

CRISPR-Cas systems have also been shown to regulate endogenous genes. Li et al. demonstrated that, in a hypervirulent *K. pneumoniae* isolate, the type I-E* system targets the histidine utilisation (*hut*) operon, that upon *casABECD* deletion the *hut* operon was derepressed, biofilm formation decreased, and virulence declined in A549 and *Galleria mellonella* models [74]. Whether the I-E system of the ST147 isolates has a comparable regulatory role cannot be determined from the present data, as the spacer targets were not characterised.

The defence repertoire followed the genetic background (Supplementary Table S6). DefenseFinder profiles were identical among all members of CL-1, CL-4, and CL-5 (17, 17, and 13 systems, respectively) and differed within CL-2 and CL-3. In CL-2 (ST147), RM type I was present in five copies in S14, S18, and S22 and in four copies in S19 and S20, and MazEF was absent only in S18. S06 and S08 (ST147, CL-3), which differ by 17 alleles, differed by six systems or copies, S06 carrying Gao Hhe, PD-T4-5, TgvAB, and additional copies of RM type I and Mok Hok Sok. Because both genomes were GUNC-flagged, this difference is reported as an assembly-based observation. Both CRISPR-Cas systems and the defence components shared by the ST147 isolates were also present in the unflagged isolates S09, S10, and S18, so these findings were not restricted to GUNC-flagged genomes. Each of the three main lineages (ST147, ST307, and ST17) carried systems that were present in all of its isolates and absent from the other two lineages (three, seven, and ten systems, respectively, including Druantia III, Gabija, and Hachiman), which indicates that the defence repertoire of this collection was largely lineage-specific. A similar diversity was reported in environmental and clinical *K. pneumoniae* isolates from hospital intensive care units in Ghana. In the environmental isolate NS2, 12 defence systems were detected, and five were detected in the clinical isolate PS4, and it was emphasised that the PADLOC, DefenseFinder, and TADB 3.0 tools agreed in classification but differed in subtyping [75].

#### 4.8.2. Anti-Defence and Mobile Elements

Anti-defence records were present in every isolate, and their content varied among genetic clusters (Supplementary Table S6). Jiang et al. detected the chromosomal type I-E system in 99.7% of strains, the plasmid-borne type IV-A3 system in 41.8%, prophage-borne AcrIE8.1 in 40.1%, and plasmid-borne AcrIE9.2 in 54.2% in 1012 CG147 genomes [71]. Conjugation experiments showed that the I-E and IV-A3 systems significantly reduced the transfer frequency of the pKpQIL plasmid (*p* < 0.001), whereas AcrIE8.1 and AcrIE9.2 significantly increased it. In our study, AcrIE9 and AcrIE8 records were found in seven and six of the 13 ST147 isolates, and eight of the 13 I-E-positive isolates (S06, S08, S14, S18, S19, S20, S22, and S23) carried at least one of them. The five CL-1 isolates (S09, S10, S24, S25, and S26) carried an I-E system but neither record, whereas AcrIE9 was also recorded in seven of the eight isolates without any CRISPR-Cas system. Anti-CRISPR records and CRISPR-Cas systems therefore co-occurred in the same genomes but were not restricted to one another, and an anti-CRISPR annotation does not in itself demonstrate inhibition of the I-E system.

The total IS element count ranged from 20 to 144 (Supplementary Table S3), and the counts varied by no more than nine within any genetic cluster (CL-1 65 to 69, CL-2 67 to 76, CL-3 62 to 69, CL-4 32 to 37, and CL-5 27 to 35). The highest counts were in the singleton isolates S11 (ST437, 144) and S16 (ST101, 101), and S07 (ST307, singleton), which carries the 550,114 bp megaplasmid, had 79 elements against 25 to 39 in the other ST307 isolates. IS counts were therefore similar within clusters and differed between lineages. Senbadejo et al. reported that the environmental isolate NS2, compared with the clinical isolate PS4, had a larger genome, more plasmids, and more mobile elements [75]. As an IS count describes insertion sequence content and not transfer events, it was not taken as evidence of horizontal transfer in our isolates. Prophage regions were detected in every isolate (2 to 7 per genome), and none carried a CARD or VFDB match (Supplementary Table S6), so the resistance and virulence determinants described above were not located within the detected prophage regions.

##### Limitations

Certain limitations of our study should be taken into account. The sample consisted of 25 carbapenem-resistant *Klebsiella pneumoniae* isolates collected at a single centre over a defined period. This design allowed detailed resolution of the genetic clusters and the gene–element matches, but generalisation of the findings to broader geographical and temporal contexts should be supported by further multicentre studies. In addition, sequence alterations, including those in *mgrB*, were taken from polished assemblies without read-level validation, and changes within homopolymeric tracts (for example in S16 and S28) may include Nanopore-specific errors. The functional consequences of the detected alterations were not tested.

A single isolate was evaluated per patient, and within-patient diversity and microevolution were not examined. Because the clinical data were compiled retrospectively, the possible epidemiological links among isolates could not be verified at the level of a contact network, and the transmission interpretations were based on the joint assessment of genomic proximity and clinical context.

Clinical outcome data and treatment information were not available, and infection could not be distinguished from colonisation, so the clinical significance of the genomic findings for individual patients could not be assessed. No carbapenem-susceptible comparison group was included, and the genotypic features described here could therefore not be compared with those of susceptible isolates.

The virulence determinants were defined at the genotypic level, and phenotypic hypervirulence tests were kept outside the scope. Because carbapenemase enzyme activity, gene copy number, and promoter measurements were not performed, the phenotypic differences observed within the same element size group could not be fully explained on a genotypic basis.

Plasmid localisation, replicon content, and mobility were presented on the basis of the predictions of bioinformatic tools, chiefly MOB-suite. Because conjugation and transfer experiments were not performed, the predicted mobility potential was not functionally verified.

Because the genetic cluster definition was based on a predetermined allelic difference threshold, the clusters were interpreted not as definitive evidence of transmission but as priority groups for further evaluation. The pan-genome analysis reflects a single-centre collection, and the functional contribution of the strain-specific gene clusters was not demonstrated. Relatedness thresholds in cgMLST are species-specific and lack a clear biological basis, and the threshold taken from the cgMLST.org nomenclature server for *K. pneumoniae* in a recent pipeline comparison was 15 allelic differences [76], so the threshold of ≤20 applied here, which was fixed before the allelic distance matrix was examined, is more permissive, and CL-2 (20 alleles) and CL-3 (17 alleles) lie close to it.

In the assessment of assembly quality, GUNC reported chimerism in 10 isolates (S06, S08, S14, S19, S20, S22, S23, S24, S25, and S26) with clade separation scores (CSS) ranging from 0.61 to 0.69, and the CSS was 0 in the other 15 isolates. In these 10 isolates, CheckM2 completeness was 100%, the estimated contamination 0.90–1.23%, and BUSCO completeness 98.7–99.1%. GUNC evaluates the lineage homogeneity of individual contigs and is designed to detect types of contamination that other quality tools may not identify [19]. Accordingly, the CheckM2 and BUSCO values do not by themselves exclude or confirm the GUNC signal. Because the chimerism reported by GUNC was not verified by an additional method, the assembly integrity of these isolates and the gene-content results based on it should be assessed within this framework.

The frameshift alterations reported in resistance-associated genes (for example *ramR* and *nfsB*) were derived from polished consensus assemblies of Oxford Nanopore reads, and allele fractions and read-level support were not examined. Residual insertions and deletions in homopolymer regions are the characteristic errors of long-read-only assemblies and can generate spurious frameshifts [77,78]. These alterations should therefore be regarded as assembly-based calls.

The CRISPR-Cas findings were descriptive. Spacer sequences were compared among isolates, but their protospacer targets were not searched, and the functional activity of the I-E and IV-A3 systems and the relevance of the spacer content to plasmid acquisition were therefore not assessed. The sensitivity analysis of the cluster threshold (Supplementary Table S8) was performed after the main analysis and was used only to describe how stable the cluster assignments were, not to redefine the clusters.

Finally, the defence system, prophage, and accessory genome calls were obtained with particular software versions and databases, and version and database updates may affect some calls. Despite these limitations, the joint use of multiple independent tools and the consistent reporting of the findings at the isolate level support the reliability of the results presented.

## 5. Conclusions

In 25 carbapenem-resistant *Klebsiella pneumoniae* isolates from a single tertiary hospital, ST147 was the dominant sequence type (13 isolates, 52%), and five genetic clusters were defined from 2335-locus allele profiles. Carbapenemase genes were carried mainly on conjugative pAA405 (*blaNDM-5*) and pAA002 (*blaOXA-48*) elements, and the co-occurrence of *blaNDM-5* with the aerobactin operon in 15 isolates extended beyond ST147 to S07 (ST307) and S11 (ST437). Isolates within the same genetic cluster differed in carbapenemase content and in susceptibility to amikacin, colistin, and ceftazidime-avibactam, and isolate pairs with zero allelic differences differed in total gene and insertion sequence counts.

The study combined long-read assemblies, a shared core-genome allele profile, assignment of carbapenemase-carrying elements to plasmid families, chromosomal contigs, and an IncX3 element, and isolate-level phenotypic data within a single framework. This allowed genotype and phenotype to be compared for each isolate and not only at the level of the sequence type.

These findings show that sequence type, carbapenemase gene, and virulence locus each describe only part of the diversity in the collection, and that genomic proximity alone does not establish transmission. The results are genotypic and descriptive and are based on 25 isolates from a single centre, without clinical outcome data, a distinction between infection and colonisation, or a carbapenem-susceptible comparison group. Isolate-level genomic characterisation of this kind may support surveillance of carbapenem-resistant *K. pneumoniae* in which cluster membership, carrying element, and resistance and virulence content are evaluated together.

## Figures and Tables

**Table 1:** Molecular typing and genetic cluster assignment based on 2335-locus allele profiles (n = 25).

Isolate	ST	KL/O Type	*wzi*	Cluster
S04	ST17	KL147/O2β	*wzi349*	CL-4
S05	ST17	KL147/O2β	*wzi349*	CL-4
S06	ST147	KL64/O2α	*wzi64*	CL-3
S07	ST307	KL102/O2β	*wzi173*	Singleton
S08	ST147	KL64/O2α	*wzi64*	CL-3
S09	ST147	KL64/O2α	*wzi64*	CL-1
S10	ST147	KL64/O2α	*wzi64*	CL-1
S11	ST437	KL47/O13	*wzi209*	Singleton
S12	ST16	KL51/O3γ	*wzi50*	Singleton
S13	ST307	KL102/O2β	*wzi173*	Singleton
S14	ST147	KL64/O2α	*wzi64*	CL-2
S15	ST307	KL102/O2β	*wzi173*	CL-5
S16	ST101	KL17/O2α	*wzi137*	Singleton
S17	ST307	KL102/O2β	*wzi173*	CL-5
S18	ST147	KL64/O2α	*wzi64*	CL-2
S19	ST147	KL64/O2α	*wzi64*	CL-2
S20	ST147	KL64/O2α	*wzi64*	CL-2
S21	ST17	KL147/O2β	*wzi349*	CL-4
S22	ST147	KL64/O2α	*wzi64*	CL-2
S23	ST147	KL64/O2α	*wzi64*	Singleton
S24	ST147	KL64/O2α	*wzi64*	CL-1
S25	ST147	KL64/O2α	*wzi64*	CL-1
S26	ST147	KL64/O2α	*wzi64*	CL-1
S27	ST307	KL102/O2β	*wzi173*	CL-5
S28	ST307	KL102/O2β	*wzi173*	Singleton

ST, sequence type. KL, capsular locus. CL, genetic cluster. Clusters were defined at a threshold of ≤20 allelic differences by single linkage based on 2335-locus allele profiles. The reference scheme comprised 2358 loci, of which 2335 were retained after excluding 23 loci that could not be called in 3 to 14 isolates. Pairwise allelic differences are shown in Figure 2B,C.

**Table 2:** Carbapenemase profile, Kleborate outputs and 16S rRNA methyltransferase genes (n = 25).

Isolate	ST	Carbapenemase	Kleborate Gene Count	16S-RMTase	Resistance Score	Virulence Score
S04	ST17	–	7	Not detected	1	0
S05	ST17	NDM-7	8	Not detected	2	0
S06	ST147	NDM-5+OXA-48	19	*rmtF1*	2	4
S07	ST307	NDM-5+OXA-48	20	Not detected	2	3
S08	ST147	NDM-5+OXA-48	15	Not detected	2	4
S09	ST147	NDM-5+OXA-48	19	*rmtF1*	2	4
S10	ST147	NDM-5+OXA-48	19	*rmtF1*	2	4
S11	ST437	NDM-5+OXA-48	16	Not detected	2	4
S12	ST16	OXA-48	3	Not detected	2	0
S13	ST307	KPC-3	6	Not detected	2	1
S14	ST147	NDM-5+OXA-48	19	*rmtF1*	3	4
S15	ST307	KPC-3	2	Not detected	2	0
S16	ST101	NDM-1+OXA-48	13	*rmtC*	3	1
S17	ST307	KPC-3	2	Not detected	2	0
S18	ST147	NDM-5	15	*rmtF1*	3	4
S19	ST147	NDM-5+OXA-48	20	*rmtF1*	2	4
S20	ST147	NDM-5+OXA-48	20	*rmtF1*	2	4
S21	ST17	NDM-7	8	Not detected	2	0
S22	ST147	NDM-5+OXA-48	19	*rmtF1*	3	4
S23	ST147	KPC-3+NDM-5+OXA-48	26	*rmtF1*	2	4
S24	ST147	NDM-5+OXA-48	19	*rmtF1*	2	4
S25	ST147	NDM-5+OXA-48	22	*rmtF1*	2	4
S26	ST147	NDM-5+OXA-48	22	*rmtF1*	2	4
S27	ST307	KPC-3	7	Not detected	2	0
S28	ST307	KPC-3	1	Not detected	2	2

ST, sequence type. Values in the carbapenemase, gene count, resistance score and virulence score columns are based on Kleborate outputs. 16S-RMTase refers to genes encoding 16S rRNA methyltransferase enzymes. A dash (–) in the carbapenemase column indicates that no carbapenemase gene was detected in the isolate. The virulence score is a numerical value calculated by Kleborate that reflects the virulence potential of the isolate.

**Table 3:** Plasmid localisation, replicon content, mobility and conjugation potential of carbapenemase-carrying genetic elements.

Isolate	ST	CL	Carbapenemase(s)	Carrying Element	Element Size (bp)	Circularity	Replicon(s)	Mobility	Carbapenemase Distribution
S04	ST17	CL-4	None	—	—	—	—	—	—
S05	ST17	CL-4	NDM-7	IncX3 element (non-pAA452)	46,161	Circular	IncX3	Not classified as a plasmid by MOB-suite	Single element
S06	ST147	CL-3	NDM-5 + OXA-48	pAA405/pAA002	346,454/68,942	Circular/Circular	IncFIB+IncHI1B/IncL/M	Conjugative/Conjugative	Two separate plasmids
S07	ST307	Singleton	NDM-5 + OXA-48	Megaplasmid (joined to chromosomal contig 2)	550,114	Circular	IncFIB+IncFIB+IncFII+IncHI1B	Chromosomally integrated	Single element
S08	ST147	CL-3	NDM-5 + OXA-48	pAA405/pAA002	348,579/68,942	Circular/Circular	IncFIB+IncHI1B/IncL/M	Conjugative/Conjugative	Two separate plasmids
S09	ST147	CL-1	NDM-5 + OXA-48	pAA405/pAA002	367,492/68,942	Circular/Circular	IncFIB+IncHI1B/IncL/M	Conjugative/Conjugative	Two separate plasmids
S10	ST147	CL-1	NDM-5 + OXA-48	pAA405/pAA002	367,492/68,942	Circular/Circular	IncFIB+IncHI1B/IncL/M	Conjugative/Conjugative	Two separate plasmids
S11	ST437	Singleton	NDM-5 + OXA-48	pAA405/pAA002	361,371/65,499	Circular/Circular	IncFIB+IncHI1B/IncL/M	Conjugative/Conjugative	Two separate plasmids
S12	ST16	Singleton	OXA-48	pAA002	63,589	Circular	IncL/M	Conjugative	Single plasmid
S13	ST307	Singleton	KPC-3	pAA018	147,568	Circular	IncFIB+IncFII (rep2183)	Conjugative	Single plasmid
S14	ST147	CL-2	NDM-5 + OXA-48	pAA405/pAA002	349,462/68,942	Circular/Circular	IncFIB+IncHI1B/IncL/M	Conjugative/Conjugative	Two separate plasmids
S15	ST307	CL-5	KPC-3	pAA018	69,799	Circular	IncFIB	Non-mobilizable	Single plasmid
S16	ST101	Singleton	NDM-1 + OXA-48	pAA046/pAA002	109,897/64,913	Circular/Circular	IncFIB+IncFII+rep2272/IncL/M	Conjugative/Conjugative	Two separate plasmids
S17	ST307	CL-5	KPC-3	pAA018	69,799	Circular	IncFIB	Non-mobilizable	Single plasmid
S18	ST147	CL-2	NDM-5	pAA405	351,373	Circular	IncFIB+IncHI1B	Conjugative	Single plasmid
S19	ST147	CL-2	NDM-5 + OXA-48	pAA405/pAA002	350,811/68,942	Circular/Circular	IncFIB+IncHI1B/IncL/M	Conjugative/Conjugative	Two separate plasmids
S20	ST147	CL-2	NDM-5 + OXA-48	pAA405/pAA002	351,638/68,942	Circular/Circular	IncFIB+IncHI1B/IncL/M	Conjugative/Conjugative	Two separate plasmids
S21	ST17	CL-4	NDM-7	IncX3 element (non-pAA452)	46,161	Circular	IncX3	Not classified as a plasmid by MOB-suite	Single element
S22	ST147	CL-2	NDM-5 + OXA-48	pAA405/pAA002	351,996/68,942	Circular/Circular	IncFIB+IncHI1B/IncL/M	Conjugative/Conjugative	Two separate plasmids
S23	ST147	Singleton	NDM-5 + KPC-3 + OXA-48	pAA405/pAA018/pAA002	367,492/89,256/68,942	Circular/Circular/Circular	IncFIB+IncHI1B/IncFIB/IncL/M	Conjugative/Non-mobilizable/Conjugative	Three separate plasmids
S24	ST147	CL-1	NDM-5 + OXA-48	pAA405/pAA002	367,493/68,942	Circular/Circular	IncFIB+IncHI1B/IncL/M	Conjugative/Conjugative	Two separate plasmids
S25	ST147	CL-1	NDM-5 + OXA-48	pAA405/pAA002	367,492/68,942	Circular/Circular	IncFIB+IncHI1B/IncL/M	Conjugative/Conjugative	Two separate plasmids
S26	ST147	CL-1	NDM-5 + OXA-48	Chromosomally integrated pAA405-like element/pAA002	492,660/68,942	Circular/Circular	IncFIB+IncHI1B (chromosomal)/IncL/M	Chromosomally integrated/Conjugative	Chromosomally integrated + one independent plasmid
S27	ST307	CL-5	KPC-3	pAA018	89,256	Circular	IncFIB	Non-mobilizable	Single plasmid
S28	ST307	Singleton	KPC-3	pAA018	105,372	Circular	IncFIB+IncFII (rep2183)	Conjugative	Single plasmid

CL, genetic cluster. ST, sequence type. Plasmid names are given according to the MOB-suite primary cluster identifier. Mobility classification was performed with MOB-suite based on the presence of relaxase, mating pair formation (MPF) type and origin of transfer (*oriT*). Circularity indicates whether the contig carrying the element was closed (circular) in the final assembly, as recorded in the assembly status of each isolate. In the carbapenemase distribution column, “—” indicates that no carbapenemase was detected. In S05 and S21, the 46,161-bp IncX3 element carrying *bla_NDM-7_* was located on contig 3 of the chromosomal assembly and was not included in the plasmid inventory by the repeat element filter. In S07, the carbapenemases are located on a four-replicon megaplasmid assembled as joined to a chromosomal contig. In S26, *bla_NDM-5_* was found on a chromosomally integrated pAA405-like element not classified as a plasmid by MOB-suite, and no separate NDM plasmid was detected.

**Table 4:** Isolate-level distribution of aerobactin, yersiniabactin and colibactin loci (n = 25).

Isolate (ST, CL)	*iuc*	*ybt*	*clb*
S04 (ST17, CL-4)	–	–	–
S05 (ST17, CL-4)	–	–	–
S06 (ST147, CL-3)	+	+	–
S07 (ST307, Singleton)	+	–	–
S08 (ST147, CL-3)	+	+	–
S09 (ST147, CL-1)	+	+	–
S10 (ST147, CL-1)	+	+	–
S11 (ST437, Singleton)	+	+	–
S12 (ST16, Singleton)	–	–	–
S13 (ST307, Singleton)	–	+	–
S14 (ST147, CL-2)	+	+	–
S15 (ST307, CL-5)	–	–	–
S16 (ST101, Singleton)	–	+	–
S17 (ST307, CL-5)	–	–	–
S18 (ST147, CL-2)	+	+	–
S19 (ST147, CL-2)	+	+	–
S20 (ST147, CL-2)	+	+	–
S21 (ST17, CL-4)	–	–	–
S22 (ST147, CL-2)	+	+	–
S23 (ST147, Singleton)	+	+	–
S24 (ST147, CL-1)	+	+	–
S25 (ST147, CL-1)	+	+	–
S26 (ST147, CL-1)	+	+	–
S27 (ST307, CL-5)	–	–	–
S28 (ST307, Singleton)	–	+	+

“+” indicates that the relevant locus was called positive and “–” that it was called negative. ST, sequence type. CL, genetic cluster. The *iuc*, *ybt* and *clb* columns indicate the aerobactin (*iucABCD-iutA*), yersiniabactin (*ybt*) and colibactin (*clb*) loci, respectively. The complete *iucABCD-iutA* operon was recorded in *iuc*-positive isolates. Determinants in the same row were detected in the same isolate, and this co-occurrence does not imply that the determinants are located on the same genetic element.

**Table 5:** Chromosomal alterations associated with carbapenem, quinolone and colistin resistance and the corresponding colistin and ciprofloxacin phenotypes (n = 25).

Isolate (ST, CL)	Porin Alterations	QRDR Alterations	Colistin-Associated Alteration	Colistin MIC (mg/L)	Colistin	Ciprofloxacin MIC (mg/L)
S04 (ST17, CL-4)	Point mutations	Not detected	None	0.5	S	>32 (R)
S05 (ST17, CL-4)	Point mutations	Not detected	None	0.25	S	>32 (R)
S06 (ST147, CL-3)	*ompK36* ins (D135DGD), *ompK35* R60LfsTer31	*gyrA* S83I, *parC* S80I	*pmrB* T157P (AMRFinderPlus)	4	R	>32 (R)
S07 (ST307, Singleton)	Point mutations	*gyrA* S83I, *parC* S80I	None	0.25	S	>32 (R)
S08 (ST147, CL-3)	*ompK36* ins (D135DGD), *ompK35* R60LfsTer31	*gyrA* S83I, *parC* S80I	None	0.25	S	>32 (R)
S09 (ST147, CL-1)	*ompK36* ins (D135DGD), *ompK35* R60LfsTer31	*gyrA* S83I, *parC* S80I	None	0.5	S	>32 (R)
S10 (ST147, CL-1)	*ompK36* ins (D135DGD), *ompK35* R60LfsTer31	*gyrA* S83I, *parC* S80I	None	0.25	S	>32 (R)
S11 (ST437, Singleton)	*ompK36* ins (D135DGD)	*gyrA* S83I, *parC* S80I	None	1	S	>32 (R)
S12 (ST16, Singleton)	*ompK36* ins (D135DGD), *ompK35* W79Ter	*gyrA* D87N+S83F, *parC* E84K	None	0.25	S	>32 (R)
S13 (ST307, Singleton)	*ompK36* ins (D135DGD), *ompK35* Y197Ter	*gyrA* S83I, *parC* S80I	None	0.25	S	>32 (R)
S14 (ST147, CL-2)	*ompK36* ins (D135DGD), *ompK35* R60LfsTer31	*gyrA* S83I, *parC* S80I	*mgrB* c.T132del (Kleborate), 8162-bp insertion	4	R	>32 (R)
S15 (ST307, CL-5)	*ompK36* ins (D135DGD), *ompK35* Y197Ter	*gyrA* S83I, *parC* S80I	None	0.25	S	>32 (R)
S16 (ST101, Singleton)	*ompK35* G62AfsTer1	*gyrA* D87N+S83Y, *parC* S80I	*mgrB* p.Lys2fs (Kleborate), confirmed in assembly	32	R	>32 (R)
S17 (ST307, CL-5)	*ompK36* ins (D135DGD), *ompK35* Y197Ter	*gyrA* S83I, *parC* S80I	None	0.25	S	>32 (R)
S18 (ST147, CL-2)	*ompK36* ins (D135DGD), *ompK35* R60LfsTer31	*gyrA* S83I, *parC* S80I	*mgrB* c.T132del (Kleborate), 8162-bp insertion	4	R	>32 (R)
S19 (ST147, CL-2)	*ompK36* ins (D135DGD), *ompK35* R60LfsTer31	*gyrA* S83I, *parC* S80I	*pmrB* T157P (AMRFinderPlus)	8	R	>32 (R)
S20 (ST147, CL-2)	*ompK36* ins (D135DGD), *ompK35* R60LfsTer31	*gyrA* S83I, *parC* S80I	*pmrB* T157P (AMRFinderPlus)	8	R	>32 (R)
S21 (ST17, CL-4)	Point mutations	Not detected	None	0.25	S	>32 (R)
S22 (ST147, CL-2)	*ompK36* ins (D135DGD), *ompK36* Q313Ter, *ompK35* R60LfsTer31	*gyrA* S83I, *parC* S80I	*mgrB* c.T132del (Kleborate), 8162-bp insertion	4	R	>32 (R)
S23 (ST147, Singleton)	*ompK36* ins (D135DGD), *ompK35* R60LfsTer31	*gyrA* S83I, *parC* S80I	None	0.5	S	>32 (R)
S24 (ST147, CL-1)	*ompK36* ins (D135DGD), *ompK35* R60LfsTer31	*gyrA* S83I, *parC* S80I	None	0.25	S	>32 (R)
S25 (ST147, CL-1)	*ompK36* ins (D135DGD), *ompK35* R60LfsTer31	*gyrA* S83I, *parC* S80I	None	1	S	>32 (R)
S26 (ST147, CL-1)	*ompK36* ins (D135DGD), *ompK35* R60LfsTer31	*gyrA* S83I, *parC* S80I	None	0.25	S	>32 (R)
S27 (ST307, CL-5)	*ompK36* ins (D135DGD), *ompK35* Y197Ter	*gyrA* S83I, *parC* S80I	None	0.5	S	>32 (R)
S28 (ST307, Singleton)	*ompK36* ins (D135DGD), *ompK35* Y197Ter	*gyrA* S83I, *parC* S80I	None	0.25	S	>32 (R)

ST, sequence type. CL, genetic cluster. QRDR, quinolone resistance-determining region. MIC, minimum inhibitory concentration. R, resistant. S, susceptible. Colistin MIC was determined by broth microdilution and ciprofloxacin MIC by gradient test. Only selected inactivation and insertion records are presented in the porin column. Colistin-associated alterations are shown with the tool that reported them. For S14, S18 and S22, the Kleborate call c.T132del corresponds to an 8162-bp insertion within *mgrB* identified in the assemblies, and for S16 the call of Kleborate and the K2E record of AMRFinderPlus were reconciled by assembly inspection (single-adenine deletion in a poly-A tract). The *mgrB* alterations of the susceptible isolates S11 (1066-bp insertion) and S28 (p.Lys43fs) are not shown. No QRDR alteration was recorded in S04, S05 and S21, all of which were ciprofloxacin-resistant.

## Data Availability

The data presented in this study are openly available in the NCBI BioProject database under accession number PRJNA1538236 (https://www.ncbi.nlm.nih.gov/bioproject/PRJNA1538236, accessed on 16 October 2026). Individual isolate records have been registered under BioSample accession numbers SAMN63818781 to SAMN63818805. Raw Oxford Nanopore sequencing reads and genome assemblies have been submitted to NCBI under the same BioProject. The BioSample, read, and assembly records will be made publicly available upon genome release, and the assembly accession numbers will be provided upon completion of processing.

## Supplementary Materials

The following supporting information can be downloaded at https://www.mdpi.com/article/10.3390/microorganisms14102402/s1. Figure S1: UPGMA dendrogram of 25 carbapenem-resistant *Klebsiella pneumoniae* isolates based on pairwise allelic distances derived from 2335-locus allelic profiles. Figure S2: Circular dendrogram of 25 carbapenem-resistant *Klebsiella pneumoniae* isolates based on 2335-locus allelic profiles. Table S1: Clinical and demographic characteristics (n = 25). Table S2: Antimicrobial susceptibility data of carbapenem-resistant *Klebsiella pneumoniae* isolates (n = 25). (a) β-lactams and β-lactam/β-lactamase inhibitor combinations. (b) Non-β-lactam agents. Table S3: Selected resistance determinants and mobile genetic element counts (n = 25). Table S4: Virulence determinants and Kleborate virulence scores (n = 25). Table S5: Genome assembly, quality and pan-genome metrics (n = 25). Table S6: Accessory genome components (n = 25). Table S7: Integrated genotypic table of carbapenem-resistant *Klebsiella pneumoniae* isolates (n = 25). Table S8: Sensitivity of cgMLST-based cluster assignment to the allelic distance threshold in 25 carbapenem-resistant *Klebsiella pneumoniae* isolates. Table S9: GUNC screening results for the 25 carbapenem-resistant *Klebsiella pneumoniae* genomes. Table S10: Antimicrobial resistance elements detected in the 25 isolates, with identity, coverage, functional status and genomic location. Table S11: Integrated isolate-level summary of clonal background, resistance phenotype by antimicrobial class, resistance determinants, carbapenemase-carrying plasmid elements, virulence traits and CRISPR-Cas systems of the 25 carbapenem-resistant *Klebsiella pneumoniae* isolates. Table S12: CRISPR arrays and spacer content of the 30 trusted CRISPR arrays identified in 17 of the 25 carbapenem-resistant *Klebsiella pneumoniae* isolates. Data S1: Spacer sequences of the 30 trusted CRISPR arrays (FASTA).

## Abbreviations

CRKP	carbapenem-resistant *Klebsiella pneumoniae*.
cgMLST	core-genome multilocus sequence typing.
IS	insertion sequence.
KL	capsular locus.
KPC	*Klebsiella pneumoniae* carbapenemase.
MIC	minimum inhibitory concentration.
MLST	multilocus sequence typing.
NDM	New Delhi metallo-β-lactamase.
ST	sequence type.
WGS	whole-genome sequencing.
