# Supplementary material for: Genomic Characterisation of Carbapenem-Resistant Klebsiella pneumoniae Clinical Isolates from a Tertiary-Care Hospital in Türkiye
Source: Microorganisms. 2026 Oct 20;14(10):2402. doi: 10.3390/microorganisms14102402 (PMC13707901; doi:10.3390/microorganisms14102402)
Supplement: Supplementary file 1 [file microorganisms-14-02402-s001.zip › Supplementary Tables S1-S12.pdf]

Supplementary Table S1. Clinical and demographic characteristics (n = 25)

Isolate	Age	Sex	Sampling date	Patient setting	ICU	Specimen type	Unit
**S04**	64	Female	2023-10	Inpatient	No	Blood culture	General Surgery Ward
**S05**	24	Male	2023-10	Inpatient	Yes	Blood culture	Anaesthesiology ICU
**S06**	48	Male	2023-10	Inpatient	No	Blood culture	Medical Oncology Ward
**S07**	76	Male	2023-11	Inpatient	No	Sputum	Infectious Diseases Ward
**S08**	42	Male	2023-11	Inpatient	Yes	Tracheal aspirate	Anaesthesiology ICU
**S09**	83	Male	2023-11	Outpatient	No	Urine	Urology Outpatient Clinic
**S10**	25	Male	2023-12	Inpatient	No	Blood culture	Neurosurgery Ward
**S11**	70	Male	2023-12	Inpatient	Yes	Tracheal aspirate	Anaesthesiology ICU
**S12**	78	Male	2024-02	Inpatient	No	Urine	Neurology Ward
**S13**	88	Male	2024-02	Inpatient	No	Sputum	Chest Diseases Ward
**S14**	74	Female	2024-03	Outpatient	No	Urine	Internal Medicine Outpatient Clinic
**S15**	66	Male	2024-03	Inpatient	Yes	Bronchoalveolar lavage	Anaesthesiology ICU
**S16**	1	Female	2024-03	Inpatient	No	Tracheal aspirate	Paediatrics Ward
**S17**	60	Female	2024-03	Inpatient	No	Urine	Medical Oncology Ward
**S18**	62	Male	2024-05	Inpatient	No	Tissue	Orthopaedics and Traumatology Ward
**S19**	44	Male	2024-05	Inpatient	No	Blood culture	Medical Oncology Ward
**S20**	83	Male	2024-05	Inpatient	No	Urine	Rheumatology Ward
**S21**	69	Male	2024-05	Inpatient	No	Wound	Orthopaedics and Traumatology Ward
**S22**	19	Male	2024-07	Inpatient	Yes	Tracheal aspirate	Internal Medicine ICU
**S23**	86	Male	2024-08	Inpatient	No	Urine	Nephrology Ward
**S24**	65	Male	2024-08	Inpatient	Yes	Peritoneal fluid	General Surgery ICU
**S25**	76	Male	2024-09	Inpatient	No	Urine	Infectious Diseases Ward
**S26**	59	Male	2024-09	Inpatient	Yes	Blood culture	Internal Medicine ICU
**S27**	22	Male	2024-10	Inpatient	No	Blood culture	Palliative Care Unit
**S28**	20	Female	2024-10	Inpatient	Yes	Tracheal aspirate	Internal Medicine ICU

ICU, intensive care unit. Age is given in years. Sampling dates are given in year-month format to protect patient confidentiality. One isolate per patient was included in the study.

Supplementary Table S2. Antimicrobial susceptibility data of carbapenem-resistant Klebsiella pneumoniae isolates (n = 25). (a) β-lactams and β-lactam/β-lactamase inhibitor combinations. (b) Non-β-lactam agents

(a) β-lactams

Isolate	Amoxicillin-clavulanate	Piperacillin-tazobactam	Cefuroxime	Cefoxitin	Ceftriaxone	Ceftazidime	Cefepime	Ertapenem	Imipenem	Meropenem	Ceftazidime-avibactam
S04	>32 (R)	>64 (R)	>32 (R)	>32 (R)	>32 (R)	>16 (R)	>16 (R)	>4 (R)	>8 (R)	24 (R)	0.75 (S)
S05	>32 (R)	>64 (R)	>32 (R)	>32 (R)	>32 (R)	>16 (R)	>16 (R)	>4 (R)	>8 (R)	32 (R)	32 (R)
S06	>32 (R)	>64 (R)	>32 (R)	>32 (R)	>32 (R)	>16 (R)	>16 (R)	>4 (R)	>8 (R)	8 (I)	>256 (R)
S07	>32 (R)	>64 (R)	>32 (R)	>32 (R)	>32 (R)	>16 (R)	>16 (R)	>4 (R)	>8 (R)	16 (R)	256 (R)
S08	>32 (R)	>64 (R)	>32 (R)	>32 (R)	>32 (R)	>16 (R)	>16 (R)	>4 (R)	>8 (R)	>32 (R)	>256 (R)
S09	>32 (R)	>64 (R)	>32 (R)	>32 (R)	>32 (R)	>16 (R)	>16 (R)	>4 (R)	>8 (R)	>32 (R)	>256 (R)
S10	>32 (R)	>64 (R)	>32 (R)	>32 (R)	>32 (R)	>16 (R)	>16 (R)	>4 (R)	>8 (R)	32 (R)	>256 (R)
S11	>32 (R)	>64 (R)	>32 (R)	>32 (R)	>32 (R)	>16 (R)	>16 (R)	>4 (R)	>8 (R)	>32 (R)	>256 (R)
S12	>32 (R)	>64 (R)	>32 (R)	>32 (R)	>32 (R)	>16 (R)	>16 (R)	>4 (R)	>8 (R)	16 (R)	0.38 (S)
S13	>32 (R)	>64 (R)	>32 (R)	>32 (R)	>32 (R)	>16 (R)	>16 (R)	>4 (R)	>8 (R)	16 (R)	0.75 (S)
S14	>32 (R)	>64 (R)	>32 (R)	>32 (R)	>32 (R)	>16 (R)	>16 (R)	>4 (R)	>8 (R)	>32 (R)	>256 (R)
S15	>32 (R)	>64 (R)	>32 (R)	>32 (R)	>32 (R)	>16 (R)	>16 (R)	>4 (R)	>8 (R)	32 (R)	>256 (R)
S16	>32 (R)	>64 (R)	>32 (R)	>32 (R)	>32 (R)	>16 (R)	>16 (R)	>4 (R)	>8 (R)	>32 (R)	>256 (R)
S17	>32 (R)	>64 (R)	>32 (R)	>32 (R)	>32 (R)	>16 (R)	>16 (R)	>4 (R)	>8 (R)	32 (R)	0.75 (S)
S18	>32 (R)	>64 (R)	>32 (R)	>32 (R)	>32 (R)	>16 (R)	>16 (R)	>4 (R)	>8 (R)	>32 (R)	>256 (R)
S19	>32 (R)	>64 (R)	>32 (R)	>32 (R)	>32 (R)	>16 (R)	>16 (R)	>4 (R)	>8 (R)	>32 (R)	>256 (R)
S20	>32 (R)	>64 (R)	>32 (R)	>32 (R)	>32 (R)	>16 (R)	>16 (R)	>4 (R)	>8 (R)	>32 (R)	>256 (R)
S21	>32 (R)	>64 (R)	>32 (R)	>32 (R)	>32 (R)	>16 (R)	>16 (R)	>4 (R)	>8 (R)	16 (R)	32 (R)
S22	>32 (R)	>64 (R)	>32 (R)	>32 (R)	>32 (R)	>16 (R)	>16 (R)	>4 (R)	>8 (R)	>32 (R)	>256 (R)
S23	>32 (R)	>64 (R)	>32 (R)	>32 (R)	>32 (R)	>16 (R)	>16 (R)	>4 (R)	>8 (R)	>32 (R)	>256 (R)
S24	>32 (R)	>64 (R)	>32 (R)	>32 (R)	>32 (R)	>16 (R)	>16 (R)	>4 (R)	>8 (R)	>32 (R)	>256 (R)
S25	>32 (R)	>64 (R)	>32 (R)	>32 (R)	>32 (R)	>16 (R)	>16 (R)	>4 (R)	>8 (R)	>32 (R)	>256 (R)
S26	>32 (R)	>64 (R)	>32 (R)	>32 (R)	>32 (R)	>16 (R)	>16 (R)	>4 (R)	>8 (R)	>32 (R)	>256 (R)
S27	>32 (R)	>64 (R)	>32 (R)	>32 (R)	>32 (R)	>16 (R)	>16 (R)	>4 (R)	>8 (R)	32 (R)	1 (S)
S28	>32 (R)	>64 (R)	>32 (R)	>32 (R)	>32 (R)	>16 (R)	>16 (R)	>4 (R)	>8 (R)	>32 (R)	1 (S)

(b) Non-β-lactam agents

Isolate	Amikacin	Ciprofloxacin	Trimethoprim-sulfamethoxazole	Colistin (broth microdilution)	Phenotypic carbapenem resistance
S04	4 (S)	>32 (R)	>160 (R)	0.5 (S)	Resistant
S05	0.75 (S)	>32 (R)	>160 (R)	0.25 (S)	Resistant
S06	>256 (R)	>32 (R)	>160 (R)	4 (R)	Resistant
S07	8 (S)	>32 (R)	>160 (R)	0.25 (S)	Resistant
S08	8 (S)	>32 (R)	>160 (R)	0.25 (S)	Resistant
S09	>256 (R)	>32 (R)	>160 (R)	0.5 (S)	Resistant
S10	>256 (R)	>32 (R)	>160 (R)	0.25 (S)	Resistant
S11	4 (S)	>32 (R)	>160 (R)	1 (S)	Resistant
S12	0.38 (S)	>32 (R)	40 (R)	0.25 (S)	Resistant
S13	16 (R)	>32 (R)	≤20 (NI)	0.25 (S)	Resistant
S14	>256 (R)	>32 (R)	>160 (R)	4 (R)	Resistant
S15	1.5 (S)	>32 (R)	40 (R)	0.25 (S)	Resistant
S16	>256 (R)	>32 (R)	>160 (R)	32 (R)	Resistant
S17	1.5 (S)	>32 (R)	≤20 (NI)	0.25 (S)	Resistant
S18	>256 (R)	>32 (R)	>160 (R)	4 (R)	Resistant
S19	>256 (R)	>32 (R)	>160 (R)	8 (R)	Resistant
S20	>256 (R)	>32 (R)	>160 (R)	8 (R)	Resistant
S21	4 (S)	>32 (R)	>160 (R)	0.25 (S)	Resistant
S22	>256 (R)	>32 (R)	>160 (R)	4 (R)	Resistant
S23	>256 (R)	>32 (R)	>160 (R)	0.5 (S)	Resistant
S24	>256 (R)	>32 (R)	>160 (R)	0.25 (S)	Resistant
S25	>256 (R)	>32 (R)	>160 (R)	1 (S)	Resistant
S26	>256 (R)	>32 (R)	>160 (R)	0.25 (S)	Resistant
S27	4 (S)	>32 (R)	≤20 (NI)	0.5 (S)	Resistant
S28	0.75 (S)	>32 (R)	≤20 (NI)	0.25 (S)	Resistant

MIC, minimum inhibitory concentration. EUCAST, European Committee on Antimicrobial Susceptibility Testing. Values are given as MIC (mg/L) with categorical interpretation according to EUCAST clinical breakpoints v16.1 (S, susceptible, standard dosing regimen, I, susceptible, increased exposure, R, resistant, NI, not interpretable). MICs of meropenem, amikacin, ciprofloxacin and ceftazidime-avibactam were determined by gradient strip testing, colistin MIC by broth microdilution and those of the other agents by an automated system. For agents tested with the automated system, values given with ">" indicate the upper measurement limit reported by the system. Phenotypic carbapenem resistance was defined as resistance to at least one of ertapenem, imipenem and

meropenem. The meropenem MIC of 8 mg/L in S06 lies between the EUCAST breakpoints (S ≤ 2 mg/L, R > 8 mg/L) and was therefore categorised as I. This isolate was classified as carbapenem-resistant on the basis of ertapenem and imipenem resistance. Trimethoprim-sulfamethoxazole MICs were reported by the automated system as the combined concentration of trimethoprim and sulfamethoxazole at a 1:19 ratio, and "≤20" and ">160" are the limits of the reported range. As EUCAST breakpoints for this combination (S ≤ 0.5 mg/L, R > 0.5 mg/L) are expressed as the concentration of trimethoprim, the reported values were divided by 20 to obtain the trimethoprim concentration. Accordingly, ≤20 corresponds to ≤1/19 mg/L, 40 to 2/38 mg/L and >160 to >8/152 mg/L (trimethoprim/sulfamethoxazole), giving trimethoprim concentrations of ≤1, 2 and >8 mg/L, respectively. Isolates with values of 40 and >160 were therefore categorised as resistant. Isolates with a value of ≤20 were designated as not interpretable, as the lower limit of the reported range (≤1 mg/L trimethoprim) is above the EUCAST breakpoint of 0.5 mg/L and does not allow categorisation as susceptible or resistant.

Supplementary Table S3. Selected resistance determinants and mobile genetic element counts (n = 25)

Isolate	β-Lactamases	Aminoglycoside-related genes	*qnr* genes	Other selected determinants	IS / integron loci
**S04**	$bla_{\text{CTX-M-15}}$, $bla_{\text{SHV-11}}$, $bla_{\text{TEM-1}}$	*aph(3'')-Ib*†, *aph(6)-Id*	*qnrB1*	*sul2*, *dfrA14*, *tet(A)*	32 / 1
**S05**	$bla_{\text{CTX-M-15}}$, $bla_{\text{NDM-7}}$, $bla_{\text{SHV-11}}$, $bla_{\text{TEM-1}}$	*aph(3'')-Ib*†, *aph(6)-Id*	*qnrB1*	*sul2*, *dfrA14*, *tet(A)*	37 / 1
**S06**	$bla_{\text{CTX-M-14}}$, $bla_{\text{CTX-M-15}}$, $bla_{\text{NDM-5}}$, $bla_{\text{OXA-48}}$, $bla_{\text{OXA-9}\dagger}$, $bla_{\text{SHV-11}}$, $bla_{\text{TEM-1}}$	*aac(6')-Ib*, *aadA1*, *aph(3'')-Ib*, *aph(3')-VI*, *aph(6)-Id*, *rmtF1*	*qnrB1*, *qnrS1*	*sul1*, *dfrA5*, *dfrA50*, *mph(A)*, *catA1*†, *catB*, *arr-2*	69 / 3
**S07**	$bla_{\text{CTX-M-14}}$, $bla_{\text{CTX-M-15}}$, $bla_{\text{NDM-5}}$, $bla_{\text{OXA-48}}$, $bla_{\text{OXA-9}\dagger}$, $bla_{\text{SHV-28}}$, $bla_{\text{TEM-1}}$	*aac(6')-Ib*, *aadA1*, *aph(3'')-Ib*, *aph(3')-VI*, *aph(6)-Id*	*qnrB1*, *qnrS1*	*sul1*, *sul2*, *dfrA5*, *dfrA14*, *mph(A)*, *catA1*†	79 / 3
**S08**	$bla_{\text{CTX-M-14}}$, $bla_{\text{CTX-M-15}}$, $bla_{\text{NDM-5}}$, $bla_{\text{OXA-48}}$, $bla_{\text{OXA-9}\dagger}$, $bla_{\text{SHV-11}}$, $bla_{\text{TEM-1}}$	*aac(6')-Ib*, *aadA1*, *aph(3'')-Ib*, *aph(3')-VI*, *aph(6)-Id*	*qnrS1*	*sul1*, *dfrA5*, *dfrA50*, *mph(A)*, *catA1*†	62 / 2
**S09**	$bla_{\text{CTX-M-14}}$, $bla_{\text{CTX-M-15}}$, $bla_{\text{NDM-5}}$, $bla_{\text{OXA-48}}$, $bla_{\text{OXA-9}\dagger}$, $bla_{\text{SHV-11}}$, $bla_{\text{TEM-1}}$	*aac(6')-Ib*, *aadA1*, *aph(3'')-Ib*, *aph(3')-Ia*, *aph(3')-VI*, *aph(6)-Id*, *rmtF1*	*qnrS1*	*sul1*, *dfrA5*, *mph(A)*, *catA1*†, *catB*, *arr-2*	65 / 3
**S10**	$bla_{\text{CTX-M-14}}$, $bla_{\text{CTX-M-15}}$, $bla_{\text{NDM-5}}$, $bla_{\text{OXA-48}}$, $bla_{\text{OXA-9}\dagger}$, $bla_{\text{SHV-11}}$, $bla_{\text{TEM-1}}$	*aac(6')-Ib*, *aadA1*, *aph(3'')-Ib*, *aph(3')-Ia*, *aph(3')-VI*, *aph(6)-Id*, *rmtF1*	*qnrS1*	*sul1*, *dfrA5*, *mph(A)*, *catA1*†, *catB*, *arr-2*	65 / 3
**S11**	$bla_{\text{CTX-M-14}}$, $bla_{\text{CTX-M-15}}$, $bla_{\text{LAP-2}}$, $bla_{\text{NDM-5}}$, $bla_{\text{OXA-48}}$, $bla_{\text{OXA-9}\dagger}$, $bla_{\text{SHV-11}}$, $bla_{\text{TEM-1}}$	*aac(6')-Ib*, *aadA1*, *aph(3')-Ia*, *aph(3')-VI*	*qnrS1*	*sul1*, *dfrA5*, *mph(A)*, *catA1*†	144 / 2
**S12**	$bla_{\text{CTX-M-15}}$, $bla_{\text{OXA-48}}$, $bla_{\text{SHV-1}}$	Not detected	Not detected	Not detected	20 / 0
**S13**	$bla_{\text{KPC-3}}$, $bla_{\text{OXA-1}}$, $bla_{\text{SHV-28}}$	*aac(6')-Ib*, *aac(6')-Ib-cr*	*qnrB1*	*dfrA14*, *catB3*†	39 / 2
**S14**	$bla_{\text{CTX-M-14}}$, $bla_{\text{CTX-M-15}}$, $bla_{\text{NDM-5}}$, $bla_{\text{OXA-48}}$, $bla_{\text{OXA-9}\dagger}$, $bla_{\text{SHV-11}}$, $bla_{\text{TEM-1}}$	*aac(6')-Ib*, *aadA1*, *aph(3'')-Ib*, *aph(3')-VI*, *aph(6)-Id*, *rmtF1*	*qnrB1*, *qnrS1*	*sul1*, *dfrA5*, *dfrA50*, *mph(A)*, *catA1*†, *catB*, *arr-2*	71 / 3
**S15**	$bla_{\text{KPC-3}}$, $bla_{\text{SHV-28}}$	Not detected	Not detected	*dfrA14*	27 / 1
**S16**	$bla_{\text{CTX-M-15}}$, $bla_{\text{NDM-1}}$, $bla_{\text{OXA-1}}$, $bla_{\text{OXA-48}}$, $bla_{\text{SHV-1}}$, $bla_{\text{TEM-1}}$, $bla_{\text{TEM-1A}}$	*aac(6')-Ib-cr*, *aac(6')-Ib-cr5*, *rmtC*	Not detected	*sul1*, *catB3*†, *arr-3*	101 / 2
**S17**	$bla_{\text{KPC-3}}$, $bla_{\text{SHV-28}}$	Not detected	Not detected	*dfrA14*	27 / 1
**S18**	$bla_{\text{CTX-M-15}}$, $bla_{\text{NDM-5}}$, $bla_{\text{OXA-9}\dagger}$, $bla_{\text{SHV-11}}$, $bla_{\text{TEM-1}}$	*aac(6')-Ib*, *aadA1*, *aph(3')-VI*, *rmtF1*	*qnrB1*, *qnrS1*	*sul1*, *dfrA5*, *dfrA50*, *mph(A)*, *catA1*†, *catB*, *arr-2*	67 / 3
**S19**	$bla_{\text{CTX-M-14}}$, $bla_{\text{CTX-M-15}}$, $bla_{\text{NDM-5}}$, $bla_{\text{OXA-48}}$, $bla_{\text{OXA-9}\dagger}$, $bla_{\text{SHV-11}}$, $bla_{\text{TEM-1}}$	*aac(6')-Ib*, *aadA1*, *aph(3'')-Ib*, *aph(3')-Ia*, *aph(3')-VI*, *aph(6)-Id*, *rmtF1*	*qnrB1*, *qnrS1*	*sul1*, *dfrA5*, *dfrA50*, *mph(A)*, *catA1*†, *catB*, *arr-2*	71 / 3
**S20**	$bla_{\text{CTX-M-14}}$, $bla_{\text{CTX-M-15}}$, $bla_{\text{NDM-5}}$, $bla_{\text{OXA-48}}$, $bla_{\text{OXA-9}\dagger}$, $bla_{\text{SHV-11}}$, $bla_{\text{TEM-1}}$	*aac(6')-Ib*, *aadA1*, *aph(3'')-Ib*, *aph(3')-Ia*, *aph(3')-VI*, *aph(6)-Id*, *rmtF1*	*qnrB1*, *qnrS1*	*sul1*, *dfrA5*, *dfrA50*, *mph(A)*, *catA1*†, *catB*, *arr-2*	73 / 3
**S21**	$bla_{\text{CTX-M-15}}$, $bla_{\text{NDM-7}}$, $bla_{\text{SHV-11}}$, $bla_{\text{TEM-1}}$	*aph(3'')-Ib*†, *aph(6)-Id*	*qnrB1*	*sul2*, *dfrA14*, *tet(A)*	37 / 1
**S22**	$bla_{\text{CTX-M-14}}$, $bla_{\text{CTX-M-15}}$, $bla_{\text{NDM-5}}$, $bla_{\text{OXA-48}}$, $bla_{\text{OXA-9}\dagger}$, $bla_{\text{SHV-11}}$, $bla_{\text{TEM-1}}$	*aac(6')-Ib*, *aadA1*, *aph(3'')-Ib*, *aph(3')-VI*, *aph(6)-Id*, *rmtF1*	*qnrB1*, *qnrS1*	*sul1*, *dfrA5*, *dfrA50*, *mph(A)*, *catA1*†, *catB*, *arr-2*	76 / 3
**S23**	$bla_{\text{CTX-M-14}}$, $bla_{\text{CTX-M-15}}$, $bla_{\text{KPC-3}}$, $bla_{\text{NDM-5}}$, $bla_{\text{OXA-1}}$,	*aac(6')-Ib*, *aac(6')-Ib-cr*, *aac(6')-Ib-cr5*, *aadA1*,	*qnrB1*, *qnrS1*	*sul1*, *dfrA5*, *dfrA14*, *dfrA50*, *tet(A)*,	81 / 5

	bla_{OXA-48}, $bla_{OXA-9†}$, bla_{SHV-11}, bla_{TEM-1}	*aph(3'')-Ib*, *aph(3')-Ia*, *aph(3')-VI*, *aph(6)-Id*, *rmtF1*		*mph(A)*, *catA1*†, *catB*, *catB3*†, *arr-2*	
**S24**	$bla_{CTX-M-14}$, $bla_{CTX-M-15}$, bla_{NDM-5}, bla_{OXA-48}, $bla_{OXA-9†}$, bla_{SHV-11}, bla_{TEM-1}	*aac(6')-Ib*, *aadA1*, *aph(3'')-Ib*, *aph(3')-Ia*, *aph(3')-VI*, *aph(6)-Id*, *rmtF1*	*qnrS1*	*sul1*, *dfrA5*, *dfrA50*, *mph(A)*, *catA1*†, *catB*, *arr-2*	65 / 3
**S25**	$bla_{CTX-M-14}$, $bla_{CTX-M-15}$, bla_{NDM-5}, bla_{OXA-48}, $bla_{OXA-9†}$, bla_{SHV-11}, bla_{TEM-1}	*aac(6')-Ib*, *aadA1*, *aph(3'')-Ib*, *aph(3')-Ia*, *aph(3')-VI*, *aph(6)-Id*, *rmtF1*	*qnrS1*	*sul1*, *dfrA5*, *dfrA50*, *mph(A)*, *catA1*†, *catB*, *arr-2*	67 / 4
**S26**	$bla_{CTX-M-14}$, $bla_{CTX-M-15}$, bla_{NDM-5}, bla_{OXA-48}, $bla_{OXA-9†}$, bla_{SHV-11}, bla_{TEM-1}	*aac(6')-Ib*, *aadA1*, *aph(3'')-Ib*, *aph(3')-Ia*, *aph(3')-VI*, *aph(6)-Id*, *rmtF1*	*qnrS1*	*sul1*, *dfrA5*, *dfrA50*, *mph(A)*, *catA1*†, *catB*, *arr-2*	69 / 4
**S27**	bla_{KPC-3}, bla_{OXA-1}, bla_{SHV-28}	*aac(6')-Ib-cr*, *aac(6')-Ib-cr5*	*qnrB1*	*dfrA14*, *tet(A)*, *catB3*†	35 / 2
**S28**	bla_{KPC-3}, bla_{SHV-28}	Not detected	Not detected	Not detected	25 / 0

IS, insertion sequence. Gene presence is shown at the isolate level. Genes are listed with the names reported in the resistance gene detection outputs, and variant names within the same family (e.g. *aac(6')-Ib*, *aac(6')-Ib-cr* and *aac(6')-Ib-cr5*) are retained as separate entries. The aminoglycoside column includes the 16S rRNA methyltransferase genes *rmtF1* and *rmtC*. Other selected determinants comprise sulphonamide (*sul*), trimethoprim (*dfrA*), tetracycline (*tet(A)*), macrolide (*mph(A)*), phenicol (*cat*) and rifampicin (*arr*) resistance genes. Intrinsic chromosomal genes (*fosA*, *oqxAB*), the bleomycin resistance gene *ble* and the macrolide-associated gene *mrx(A)* are not listed. Chromosomal point mutations are not included in this table. The last column gives the numbers of insertion sequences (ISEScan) and integron loci (IntegronFinder) recorded in each isolate. "Not detected" indicates that no gene of the corresponding category was detected. † indicates a gene call classified by AMRFinderPlus as truncated or disrupted (aph(3")-Ib and catA1 and catB3, partial-gene calls with 58%, 66.2% and 70.0% coverage, respectively, and blaOXA-9, internal stop codon). Details are given in Supplementary Table S10.

Supplementary Table S4. Virulence determinants and Kleborate virulence scores (n = 25)

Isolate	VFDB gene count	*ybt*/*irp*/*fyuA* genes	*iucABCD-iutA*	*rmpA/rmpA2 partial match, Kleborate (% of reference)*	Colibactin (*clb*)	Kleborate virulence score
**S04**	70	Not detected	Not detected	None	Negative	0
**S05**	70	Not detected	Not detected	None	Negative	0
**S06**	87	*fyuA*, *ybtE*, *ybtT*, *ybtU*, *irp1*, *irp2*, *ybtA*, *ybtP*, *ybtQ*, *ybtX*, *ybtS*	Complete operon	47%	Negative	4
**S07**	75	Not detected	Complete operon	55%	Negative	3
**S08**	87	*fyuA*, *ybtE*, *ybtT*, *ybtU*, *irp1*, *ybtA*, *ybtP*, *ybtQ*, *ybtX*, *ybtS*	Complete operon	55%	Negative	4
**S09**	87	*fyuA*, *ybtE*, *ybtT*, *ybtU*, *irp1*, *irp2*, *ybtA*, *ybtP*, *ybtQ*, *ybtX*, *ybtS*	Complete operon	47%	Negative	4
**S10**	87	*fyuA*, *ybtE*, *ybtT*, *ybtU*, *irp1*, *irp2*, *ybtA*, *ybtP*, *ybtQ*, *ybtX*, *ybtS*	Complete operon	47%	Negative	4
**S11**	79	*fyuA*, *ybtE*, *ybtT*, *ybtU*, *irp2*, *ybtA*, *ybtP*, *ybtQ*, *ybtX*, *ybtS*	Complete operon	55%	Negative	4
**S12**	63	Not detected	Not detected	None	Negative	0
**S13**	82	*fyuA*, *ybtE*, *ybtT*, *ybtU*, *irp1*, *irp2*, *ybtA*, *ybtP*, *ybtQ*, *ybtX*, *ybtS*	Not detected	None	Negative	1
**S14**	87	*fyuA*, *ybtE*, *ybtT*, *ybtU*, *irp1*, *irp2*, *ybtA*, *ybtP*, *ybtQ*, *ybtX*, *ybtS*	Complete operon	47%	Negative	4
**S15**	71	Not detected	Not detected	None	Negative	0
**S16**	85	*fyuA*, *ybtE*, *ybtT*, *ybtU*, *irp1*, *irp2*, *ybtA*, *ybtP*, *ybtQ*, *ybtX*, *ybtS*	Not detected	None	Negative	1
**S17**	70	Not detected	Not detected	None	Negative	0
**S18**	87	*fyuA*, *ybtE*, *ybtT*, *ybtU*, *irp1*, *irp2*, *ybtA*, *ybtP*, *ybtQ*, *ybtX*, *ybtS*	Complete operon	55%	Negative	4
**S19**	87	*fyuA*, *ybtE*, *ybtT*, *ybtU*, *irp1*, *irp2*, *ybtA*, *ybtP*, *ybtQ*, *ybtX*, *ybtS*	Complete operon	55%	Negative	4

**S20**	87	*fyuA*, *ybtE*, *ybtT*, *ybtU*, *irp1*, *irp2*, *ybtA*, *ybtP*, *ybtQ*, *ybtX*, *ybtS*	Complete operon	47%	Negative	4
**S21**	70	Not detected	Not detected	None	Negative	0
**S22**	87	*fyuA*, *ybtE*, *ybtT*, *ybtU*, *irp1*, *irp2*, *ybtA*, *ybtP*, *ybtQ*, *ybtX*, *ybtS*	Complete operon	47%	Negative	4
**S23**	87	*fyuA*, *ybtE*, *ybtT*, *ybtU*, *irp1*, *irp2*, *ybtA*, *ybtP*, *ybtQ*, *ybtX*, *ybtS*	Complete operon	47%	Negative	4
**S24**	87	*fyuA*, *ybtE*, *ybtT*, *ybtU*, *irp1*, *irp2*, *ybtA*, *ybtP*, *ybtQ*, *ybtX*, *ybtS*	Complete operon	55%	Negative	4
**S25**	87	*fyuA*, *ybtE*, *ybtT*, *ybtU*, *irp1*, *irp2*, *ybtA*, *ybtP*, *ybtQ*, *ybtX*, *ybtS*	Complete operon	47%	Negative	4
**S26**	87	*fyuA*, *ybtE*, *ybtT*, *ybtU*, *irp1*, *irp2*, *ybtA*, *ybtP*, *ybtQ*, *ybtX*, *ybtS*	Complete operon	60%	Negative	4
**S27**	71	Not detected	Not detected	None	Negative	0
**S28**	100	*fyuA*, *ybtE*, *ybtT*, *ybtU*, *irp1*, *irp2*, *ybtA*, *ybtP*, *ybtQ*, *ybtX*, *ybtS*	Not detected	None	Positive	2

VFDB, Virulence Factor Database. Virulence genes were screened with ABRicate v1.4.0 against VFDB (≥ 80% nucleotide identity and ≥ 80% coverage). The *ybt*/*irp*/*fyuA* column lists the yersiniabactin locus genes detected in each isolate. In S08, Kleborate reported a partial match of *irp2* (28% of the reference sequence) and in S11 a partial match of *irp1* (19%), and labelled the yersiniabactin structure of both isolates as truncated ICE*Kp*. These partial matches were not counted as presence of the genes. In the VFDB screening, *rmpA* and *rmpA2* were not detected in any isolate. A complete *rmpA*, *rmpA2*, *rmpC* or *rmpD* was detected neither by Kleborate nor by the separate gene screening, and the Kleborate RmST was 0 in all isolates. The *rmpA*/*rmpA2* column gives the fraction of the reference sequence covered by the partial matches reported by Kleborate (None, no match reported). Partial matches were not counted as presence. The salmochelin *iroBCDE* operon was not detected in any isolate, and *fimH*, *mrkABCD* and *entB* were detected in all isolates. The Kleborate virulence score (0–5) is derived from the yersiniabactin, colibactin and aerobactin loci and was not used as a phenotypic hypervirulence classification.

Supplementary Table S5. Genome assembly, quality and pan-genome metrics (n = 25)

**Isolate**	**Genome / chromosome (bp)**	**Contigs / circular / chromosome topology**	**Coverage (×) / GC (%)**	**BUSCO / completeness / contamination (%)**	**ANI (%) / CSS**	**Total genes**	**Core / soft-core / shell / cloud / unique**
**S04**	5445989 / 5256006	2 / 2 / circular	95.7 / 57	99 / 100 / 0.03	99.03 / 0	4869	4305 / 72 / 349 / 143 / 0
**S05**	5492169 / 5256011	3 / 3 / circular	244.7 / 57	99 / 100 / 0.03	99.03 / 0	4922	4305 / 72 / 355 / 190 / 0
**S06**	6096437 / 5410499	5 / 4 / linear	136.8 / 56	98.7 / 100 / 1.11	98.97 / 0.65	5027	4305 / 72 / 605 / 27 / 18
**S07**	5865560 / 5315461	2 / 2 / circular	120.2 / 57	99 / 100 / 0.24	99.03 / 0	5519	4305 / 71 / 487 / 515 / 141
**S08**	5941992 / 5411631	4 / 4 / circular	175.5 / 57	99 / 100 / 0.96	98.97 / 0.69	5006	4305 / 72 / 627 / 2 / 0
**S09**	5802030 / 5365617	3 / 3 / circular	131.3 / 57	99.1 / 100 / 0.27	98.97 / 0	4937	4305 / 72 / 560 / 0 / 0
**S10**	5802026 / 5365617	3 / 3 / circular	88.3 / 57	99.1 / 100 / 0.27	98.97 / 0	4937	4305 / 72 / 560 / 0 / 0
**S11**	6007511 / 5380377	4 / 4 / circular	165.8 / 57	99 / 100 / 0.25	98.97 / 0	5351	4305 / 58 / 282 / 370 / 336
**S12**	5409674 / 5278487	3 / 3 / circular	174.4 / 57	98.9 / 100 / 0.08	98.92 / 0	5039	4305 / 66 / 372 / 168 / 128
**S13**	5566616 / 5409335	3 / 3 / circular	255.1 / 57	99 / 100 / 0.08	99.13 / 0	4987	4305 / 72 / 562 / 30 / 18
**S14**	6115664 / 5419935	6 / 6 / circular	99.1 / 56	99 / 100 / 1.23	98.97 / 0.67	5011	4305 / 72 / 629 / 5 / 0
**S15**	5504816 / 5337920	6 / 6 / circular	93 / 57	99 / 100 / 0.13	99.03 / 0	4924	4305 / 72 / 512 / 35 / 0
**S16**	5963108 / 5407734	7 / 7 / circular	95.6 / 57	99.1 / 100 / 0.21	98.97 / 0	5335	4305 / 23 / 313 / 365 / 329
**S17**	5420195 / 5336959	4 / 4 / circular	101.8 / 57	99 / 100 / 0.12	99.08 / 0	4925	4305 / 71 / 512 / 36 / 1
**S18**	6047465 / 5418781	5 / 5 / circular	87 / 56	98.9 / 100 / 1.06	99.03 / 0	5010	4305 / 72 / 628 / 5 / 0
**S19**	6102684 / 5415419	5 / 5 / circular	130.4 / 56	99.1 / 100 / 1.14	98.97 / 0.67	5008	4305 / 72 / 629 / 2 / 0
**S20**	6110467 / 5416484	6 / 6 / circular	88.8 / 56	99.1 / 100 / 1.14	98.97 / 0.67	5011	4305 / 72 / 630 / 3 / 1

**S21**	5501887 / 5256010	4 / 4 / circular	118.4 / 57	99 / 100 / 0.03	99.03 / 0	4922	4305 / 72 / 355 / 190 / 0
**S22**	6124210 / 5423108	6 / 6 / circular	131.6 / 56	99 / 100 / 1.23	98.97 / 0.66	5015	4305 / 72 / 633 / 5 / 0
**S23**	6004527 / 5366736	6 / 6 / circular	115.4 / 57	99.1 / 100 / 1.17	98.97 / 0.67	4937	4305 / 72 / 560 / 0 / 0
**S24**	5905466 / 5366726	4 / 4 / circular	137.3 / 57	99.1 / 100 / 0.9	98.97 / 0.69	4938	4305 / 72 / 561 / 0 / 0
**S25**	5929149 / 5369466	4 / 4 / circular	138.5 / 57	99.1 / 100 / 0.92	98.97 / 0.69	4942	4305 / 72 / 562 / 2 / 1
**S26**	5929840 / 5368272	3 / 3 / circular	102.3 / 57	99.1 / 100 / 0.92	98.97 / 0.61	5332	4305 / 72 / 580 / 362 / 13
**S27**	5441003 / 5337919	4 / 4 / circular	125.4 / 57	99 / 100 / 0.09	99.08 / 0	4924	4305 / 72 / 512 / 35 / 0
**S28**	5945164 / 5629521	4 / 4 / circular	88 / 57	99 / 100 / 0.92	99.13 / 0	5308	4305 / 71 / 635 / 181 / 116

ANI, average nucleotide identity. BUSCO, Benchmarking Universal Single-Copy Orthologs. CSS, clade separation score. GC, guanine–cytosine content. Coverage denotes mean sequencing coverage. The contig column gives the total number of contigs, the number of circular contigs and the topology of the chromosomal contig. BUSCO values are based on the enterobacteriaceae_odb12.2 marker set. Completeness and contamination are presented as CheckM2 outputs, ANI as GTDB-Tk output and CSS as GUNC output. A CSS of 0 indicates that GUNC reported no clade separation signal. CSS values above the GUNC threshold of 0.45 were reported for 10 genomes (S06, S08, S14, S19, S20, S22, S23, S24, S25 and S26), all at the family level only, and the GUNC results of all 25 genomes are given in Supplementary Table S9. Total genes represent the sum of core, soft-core, shell, cloud and unique (strain-specific) genes in each isolate. As gene categories are counted separately for each isolate, column sums across isolates do not correspond to the number of gene clusters in the pan-genome.

Supplementary Table S6. Accessory genome components (n = 25)

Isolate	CRISPR-Cas subtype	Defence systems, number and types	Anti-defence systems	Secretion systems, number and types	BGCs	Prophages: total / QC / high / medium / low quality and lifestyle
**S04**	None	17<br>RM Type II(2), AbiE, DS-28, DS-9, FS GIY YIG, Hachiman, Mok Hok Sok, NLR like bNACHT01, PD-Lambda-3, PD-T4-3, PD-T7-1, RM Type I, RM Type III, RM Type IV, SDIC3, UG17	ardb klca acric11(2), acrie9, apyc1, arda ardu, psiab	6<br>T1SS(2), T2SS, T4aP, T5bSS, T6SSi	5	6 / 4 / 1 / 3 / 2 temperate (4), unassigned (2)
**S05**	None	17<br>RM Type II(2), AbiE, DS-28, DS-9, FS GIY YIG, Hachiman, Mok Hok Sok, NLR like bNACHT01, PD-Lambda-3, PD-T4-3, PD-T7-1, RM Type I, RM Type III, RM Type IV, SDIC3, UG17	ardb klca acric11(2), acrie9, apyc1, arda ardu, psiab	7<br>T1SS(2), T2SS, T4aP, T5bSS, T6SSi, pT4SSt	5	6 / 4 / 1 / 3 / 2 temperate (4), unassigned (2)
**S06**	I-E, IV-A3	23<br>RM Type I(4), Mok Hok Sok(2), Pif(2), AbiE, CRISPR-Cas I-E, CRISPR-Cas IV-A, DS-32, Druantia III, Gao Hhe, Lamassu-Mrr, MazEF, PD-T4-3, PD-T4-5, RM Type II, RM Type IV, Retron I B, TIR-III, TgvAB	ardb klca acric11(2), acrie9, apyc1, arda ardu, ardc, psiab	6<br>T1SS, T2SS, T4aP, T5bSS, T6SSi, pT4SSt	7	4 / 4 / 1 / 3 / 0 temperate (4)
**S07**	IV-A3	19<br>Mok Hok Sok(2), RM Type II(2), AbiE, CRISPR-Cas IV-A, DS-19, Gabija, Gao Tmn, Hypnos, MazEF, PD-T4-3, Pif, RM Type I, Rst TIR-NLR, SDIC4, Shango, TIR-III, TgvAB	ardb klca acric11(2), acrie9, apyc1, psiab	7<br>T1SS(2), T2SS, T4aP, T5bSS, T6SSi(2)	6	2 / 2 / 1 / 1 / 0 temperate (2)
**S08**	I-E, IV-A3	17<br>Pif(2), RM Type I(2), AbiE, CRISPR-Cas I-E, CRISPR-Cas IV-A, DS-32, Druantia III, Lamassu-Mrr, MazEF, Mok Hok Sok, PD-T4-3, RM Type II, RM Type IV, Retron I B, TIR-III	acrie8, apyc1, ardb klca acric11, ardc	6<br>T1SS, T2SS, T4aP, T5bSS, T6SSi, pT4SSt	7	4 / 4 / 0 / 4 / 0 temperate (4)

**S09**	I-E, IV-A3	17<br>Pif(2), RM Type I(2), AbiE, CRISPR-Cas I-E, CRISPR-Cas IV-A, DS-32, Druantia III, Lamassu-Mrr, MazEF, Mok Hok Sok, PD-T4-3, RM Type II, RM Type IV, Retron I B, TIR-III	apyc1, ardb klca acric11, ardc	6<br>T1SS,<br>T2SS,<br>T4aP,<br>T5bSS,<br>T6SSi,<br>pT4SSt	7	3 / 3 / 0 / 3 / 0<br>temperate (3)
**S10**	I-E, IV-A3	17<br>Pif(2), RM Type I(2), AbiE, CRISPR-Cas I-E, CRISPR-Cas IV-A, DS-32, Druantia III, Lamassu-Mrr, MazEF, Mok Hok Sok, PD-T4-3, RM Type II, RM Type IV, Retron I B, TIR-III	apyc1, ardb klca acric11, ardc	6<br>T1SS,<br>T2SS,<br>T4aP,<br>T5bSS,<br>T6SSi,<br>pT4SSt	7	3 / 3 / 0 / 3 / 0<br>temperate (3)
**S11**	IV-A3	23<br>GAPS1(2), RM Type I(2), SDIC3(2), TgvAB(2), AbiE, AbiU, CRISPR-Cas IV-A, CBASS II, DS-24, DS-34, Gao Hhe, MazEF, Mok Hok Sok, PD-T7-1, Pif, RM Type II, RM Type IV, Rst 3HP, TIR-III	ardb klca acric11(3), ardc(2), acrie9, apyc1, psiab	5<br>T2SS,<br>T4aP,<br>T5bSS,<br>T6SSi,<br>pT4SSt	7	6 / 4 / 0 / 4 / 2<br>temperate (4), unassigned (2)
**S12**	None	12<br>MazEF(2), AbiE, DS-1, FS HP, Mok Hok Sok, PD-T4-3, RM Type I, RM Type II, RM Type IV, Septu, VP1839	ardb klca acric11(2), apyc1, psiab	5<br>T1SS,<br>T2SS,<br>T4aP,<br>T5bSS,<br>T6SSi	5	6 / 5 / 1 / 4 / 1<br>temperate (5), unassigned (1)
**S13**	None	14<br>RM Type I(2), AbiE, DS-19, Gabija, Gao Tmn, Hypnos, Lamassu-Mrr, PD-T4-3, RM Type II, Retron I B, Rst TIR-NLR, SDIC4, Shango	ardb klca acric11(2), acrie9, apyc1, arda ardu, ardc, psiab	8<br>T1SS(2),<br>T2SS,<br>T4aP,<br>T5bSS,<br>T6SSi(2),<br>pT4SSt	6	3 / 3 / 0 / 3 / 0<br>temperate (3)
**S14**	I-E, IV-A3	24<br>RM Type I(5), Mok Hok Sok(2), Pif(2), AbiE, CRISPR-Cas I-E, CRISPR-Cas IV-A, DS-32, Druantia III, Gao Hhe, Lamassu-Mrr, MazEF, PD-T4-3, PD-T4-5, RM Type II, RM Type IV, Retron I B, TIR-III, TgvAB	ardb klca acric11(2), acrie8, acrie9, apyc1, arda ardu, ardc, psiab	6<br>T1SS,<br>T2SS,<br>T4aP,<br>T5bSS,<br>T6SSi,<br>pT4SSt	7	4 / 4 / 0 / 4 / 0<br>temperate (4)
**S15**	None	13<br>RM Type I(2), AbiE, DS-19, Gabija, Gao Tmn, Hypnos, PD-T4-3, RM Type II, Rst TIR-NLR, SDIC4, Shango, ShosTA	ardb klca acric11(2), acrie9, apyc1, arda ardu, hdf, psiab, ulx	6<br>T1SS(2),<br>T2SS,<br>T4aP,<br>T5bSS,<br>T6SSi	6	4 / 3 / 0 / 3 / 1<br>temperate (3), unassigned (1)

**S16**	IV-A3	16<br>HEC-09(2), MazEF(2), RM Type II(2), AbiE, CRISPR-Cas IV-A, CBASS II, Lamassu-Mrr, Mok Hok Sok, PD-Lambda-1, PD-T4-3, PD-T7-1, RM Type IV, Retron I B	ardb klca acric11(2), ardc(2), psiab(2), abc1, apyc1, arda ardu	7<br>T1SS, T2SS, T4aP, T5bSS, T6SSi(2), pT4SSt	6	7 / 3 / 0 / 3 / 4<br>unassigned (4), temperate (2), virulent (1)
**S17**	None	13<br>RM Type I(2), AbiE, DS-19, Gabija, Gao Tmn, Hypnos, PD-T4-3, RM Type II, Rst TIR-NLR, SDIC4, Shango, ShosTA	ardb klca acric11(2), acrie9, apyc1, arda ardu, psiab	6<br>T1SS(2), T2SS, T4aP, T5bSS, T6SSi	6	4 / 3 / 0 / 3 / 1<br>temperate (3), unassigned (1)
**S18**	I-E, IV-A3	23<br>RM Type I(5), Mok Hok Sok(2), Pif(2), AbiE, CRISPR-Cas I-E, CRISPR-Cas IV-A, DS-32, Druantia III, Gao Hhe, Lamassu-Mrr, PD-T4-3, PD-T4-5, RM Type II, RM Type IV, Retron I B, TIR-III, TgvAB	acrie8, acrie9, apyc1, arda ardu, ardb klca acric11, ardc, psiab	6<br>T1SS, T2SS, T4aP, T5bSS, T6SSi, pT4SSt	7	4 / 4 / 0 / 4 / 0<br>temperate (4)
**S19**	I-E, IV-A3	23<br>RM Type I(4), Mok Hok Sok(2), Pif(2), AbiE, CRISPR-Cas I-E, CRISPR-Cas IV-A, DS-32, Druantia III, Gao Hhe, Lamassu-Mrr, MazEF, PD-T4-3, PD-T4-5, RM Type II, RM Type IV, Retron I B, TIR-III, TgvAB	ardb klca acric11(2), acrie8, acrie9, apyc1, arda ardu, ardc, psiab	6<br>T1SS, T2SS, T4aP, T5bSS, T6SSi, pT4SSt	7	4 / 4 / 0 / 4 / 0<br>temperate (4)
**S20**	I-E, IV-A3	23<br>RM Type I(4), Mok Hok Sok(2), Pif(2), AbiE, CRISPR-Cas I-E, CRISPR-Cas IV-A, DS-32, Druantia III, Gao Hhe, Lamassu-Mrr, MazEF, PD-T4-3, PD-T4-5, RM Type II, RM Type IV, Retron I B, TIR-III, TgvAB	ardb klca acric11(2), acrie8, acrie9, apyc1, arda ardu, ardc, psiab	6<br>T1SS, T2SS, T4aP, T5bSS, T6SSi, pT4SSt	7	4 / 4 / 0 / 4 / 0<br>temperate (4)
**S21**	None	17<br>RM Type II(2), AbiE, DS-28, DS-9, FS GIY YIG, Hachiman, Mok Hok Sok, NLR like bNACHT01, PD-Lambda-3, PD-T4-3, PD-T7-1, RM Type I, RM Type III, RM Type IV, SDIC3, UG17	ardb klca acric11(2), acrie9, apyc1, arda ardu, psiab	7<br>T1SS(2), T2SS, T4aP, T5bSS, T6SSi, pT4SSt	5	6 / 4 / 1 / 3 / 2<br>temperate (4), unassigned (2)

**S22**	I-E, IV-A3	24<br>RM Type I(5), Mok Hok Sok(2), Pif(2), AbiE, CRISPR-Cas I-E, CRISPR-Cas IV-A, DS-32, Druantia III, Gao Hhe, Lamassu-Mrr, MazEF, PD-T4-3, PD-T4-5, RM Type II, RM Type IV, Retron I B, TIR-III, TgvAB	ardb klca acric11(2), acrie8, acrie9, apyc1, arda ardu, ardc, psiab	6<br>T1SS, T2SS, T4aP, T5bSS, T6SSi, pT4SSt	7	4 / 4 / 0 / 4 / 0<br>temperate (4)
**S23**	I-E, IV-A3	19<br>RM Type I(3), Pif(2), RM Type II(2), AbiE, CRISPR-Cas I-E, CRISPR-Cas IV-A, DS-32, Druantia III, Lamassu-Mrr, MazEF, Mok Hok Sok, PD-T4-3, RM Type IV, Retron I B, TIR-III	ardb klca acric11(2), acrie9, apyc1, arda ardu, ardc, psiab	6<br>T1SS, T2SS, T4aP, T5bSS, T6SSi, pT4SSt	7	3 / 3 / 0 / 3 / 0<br>temperate (3)
**S24**	I-E, IV-A3	17<br>Pif(2), RM Type I(2), AbiE, CRISPR-Cas I-E, CRISPR-Cas IV-A, DS-32, Druantia III, Lamassu-Mrr, MazEF, Mok Hok Sok, PD-T4-3, RM Type II, RM Type IV, Retron I B, TIR-III	apyc1, ardb klca acric11, ardc	6<br>T1SS, T2SS, T4aP, T5bSS, T6SSi, pT4SSt	7	3 / 3 / 0 / 3 / 0<br>temperate (3)
**S25**	I-E, IV-A3	17<br>Pif(2), RM Type I(2), AbiE, CRISPR-Cas I-E, CRISPR-Cas IV-A, DS-32, Druantia III, Lamassu-Mrr, MazEF, Mok Hok Sok, PD-T4-3, RM Type II, RM Type IV, Retron I B, TIR-III	apyc1, ardb klca acric11, ardc	6<br>T1SS, T2SS, T4aP, T5bSS, T6SSi, pT4SSt	7	5 / 4 / 0 / 4 / 1<br>temperate (3), virulent (1), unassigned (1)
**S26**	I-E, IV-A3	17<br>Pif(2), RM Type I(2), AbiE, CRISPR-Cas I-E, CRISPR-Cas IV-A, DS-32, Druantia III, Lamassu-Mrr, MazEF, Mok Hok Sok, PD-T4-3, RM Type II, RM Type IV, Retron I B, TIR-III	apyc1, ardb klca acric11, ardc	6<br>T1SS, T2SS, T4aP, T5bSS, T6SSi, pT4SSt	7	5 / 4 / 0 / 4 / 1<br>temperate (3), virulent (1), unassigned (1)
**S27**	None	13<br>RM Type I(2), AbiE, DS-19, Gabija, Gao Tmn, Hypnos, PD-T4-3, RM Type II, Rst TIR-NLR, SDIC4, Shango, ShosTA	ardb klca acric11(2), acrie9, apyc1, arda ardu, psiab	6<br>T1SS(2), T2SS, T4aP, T5bSS, T6SSi	6	4 / 3 / 0 / 3 / 1<br>temperate (3), unassigned (1)

**S28**	IV-A3	14<br>RM Type I(2), AbiE, CRISPR-Cas IV-A, DS-19, Gabija, Gao Tmn, Hypnos, Mok Hok Sok, PD-T4-3, RM Type II, Rst TIR-NLR, SDIC4, Shango	ardb klca acric11(2), psiab(2), acrie9, apyc1, arda ardu	8<br>T1SS(2), T2SS, T4aP, T5bSS, T6SSi(2), pT4SSt	6	6 / 5 / 1 / 4 / 1<br>temperate (5), unassigned (1)

BGC, biosynthetic gene cluster. QC, number of prophage regions passing CheckV quality control (high- and medium-quality regions). Defence and anti-defence systems were identified with DefenseFinder, CRISPR-Cas subtypes with CRISPRCasTyper, secretion systems with TXSScan, BGCs with antiSMASH and prophage regions with geNomad and CheckV. Numbers in parentheses indicate the copy number of the same system or lifestyle category in the source output. Anti-defence entries are given with the names reported by DefenseFinder. Lifestyle predictions were obtained with PhaBOX2, and "unassigned" indicates regions without a predicted lifestyle. No prophage region was classified as complete, and no CARD or VFDB gene match was detected in any prophage region. The I-E operon and arrays were located on the main chromosomal contig of the 13 ST147 isolates. The IV-A3 operon and array were located on the contig carrying blaNDM-5 in 15 isolates (S06, S07, S08, S09, S10, S11, S14, S18, S19, S20, S22, S23, S24, S25 and S26) and on contigs of 296,447 bp (S16) and 200,551 bp (S28) that carried no carbapenemase gene. In S28, a second array (21 repeats, 28 bp) classified as untrusted by CRISPRCasTyper was not counted. AcrIE8 and AcrIE9 entries are listed as reported by DefenseFinder.

Supplementary Table S7. Integrated genotypic table of carbapenem-resistant Klebsiella pneumoniae isolates (n = 25)

Isolate	ST	K/O locus	Carbapenemase	Carbapenemase location	Colistin-associated alteration	Other selected chromosomal variants	Genetic cluster (≤20 alleles)	GUNC (CSS)	Coverage (×)	SRA / BioSample / GenBank
S04	ST17	KL147/O2β	None	None	None	None	CL-4	Not flagged	95.7	Pending
S05	ST17	KL147/O2β	NDM-7	blaNDM-7, IncX3 element (46,161 bp), not classified as a plasmid by MOB-suite	None	None	CL-4	Not flagged	244.7	Pending
S06	ST147	KL64/O2α	NDM-5+OXA-48	blaNDM-5, plasmid 1 (346,454 bp) / blaOXA-48, plasmid 4 (68,942 bp)	pmrB T157P (AMRFinderPlus)	OmpK36 D135DGD ins, OmpK35 R60LfsTer31, gyrA S83I, parC S80I	CL-3	Flagged (0.65)	136.8	Pending
S07	ST307	KL102/O2β	NDM-5+OXA-48	blaNDM-5 + blaOXA-48, 550,114 bp megaplasmid merged with chromosomal contig 2	None	gyrA S83I, parC S80I	Singleton	Not flagged	120.2	Pending
S08	ST147	KL64/O2α	NDM-5+OXA-48	blaNDM-5, plasmid 1 (348,579 bp) / blaOXA-48, plasmid 3 (68,942 bp)	None	OmpK36 D135DGD ins, OmpK35 R60LfsTer31, gyrA S83I, parC S80I	CL-3	Flagged (0.69)	175.5	Pending
S09	ST147	KL64/O2α	NDM-5+OXA-48	blaNDM-5, plasmid 1 (367,492 bp) / blaOXA-48, plasmid 2 (68,942 bp)	None	OmpK36 D135DGD ins, OmpK35 R60LfsTer31, gyrA S83I, parC S80I	CL-1	Not flagged	131.3	Pending
S10	ST147	KL64/O2α	NDM-5+OXA-48	blaNDM-5, plasmid 1 (367,492 bp) / blaOXA-48, plasmid 2 (68,942 bp)	None	OmpK36 D135DGD ins, OmpK35 R60LfsTer31, gyrA S83I, parC S80I	CL-1	Not flagged	88.3	Pending
S11	ST437	KL47/O13	NDM-5+OXA-48	blaNDM-5, plasmid 1 (361,371 bp) / blaOXA-48, plasmid 3 (65,499 bp)	None (mgrB 1,066-bp IS insertion after codon 38, colistin-susceptible)	OmpK36 D135DGD ins, gyrA S83I, parC S80I	Singleton	Not flagged	165.8	Pending
S12	ST16	KL51/O3γ	OXA-48	blaOXA-48, plasmid 2 (63,589 bp)	None	OmpK36 D135DGD ins, OmpK35 W79Ter, gyrA D87N+S83F, parC E84K	Singleton	Not flagged	174.4	Pending
S13	ST307	KL102/O2β	KPC-3	blaKPC-3, plasmid 1 (147,568 bp)	None	OmpK36 D135DGD ins, OmpK35 Y197Ter, gyrA S83I, parC S80I	Singleton	Not flagged	255.1	Pending
S14	ST147	KL64/O2α	NDM-5+OXA-48	blaNDM-5, plasmid 1 (349,462 bp) / blaOXA-48, plasmid 4 (68,942 bp)	mgrB 8,162-bp insertion at codon 45 (reported by Kleborate as c.T132del)	OmpK36 D135DGD ins, OmpK35 R60LfsTer31, gyrA S83I, parC S80I	CL-2	Flagged (0.67)	99.1	Pending
S15	ST307	KL102/O2β	KPC-3	blaKPC-3, plasmid 2 (69,799 bp)	None	OmpK36 D135DGD ins, OmpK35 Y197Ter, gyrA S83I, parC S80I	CL-5	Not flagged	93.0	Pending

Isolate	ST	K/O locus	Carbapenemase	Carbapenemase location	Colistin-associated alteration	Other selected chromosomal variants	Genetic cluster (≤20 alleles)	GUNC (CSS)	Coverage (×)	SRA / BioSample / GenBank
S16	ST101	KL17/O2α	NDM-1+OXA-48	blaNDM-1, plasmid 2 (109,897 bp) / blaOXA-48, plasmid 4 (64,913 bp)	mgrB Lys2fs (Kleborate), single-adenine deletion in a poly-A tract confirmed in the assembly. K2E (AMRFinderPlus, unclassified)	OmpK35 G62AfsTer1, gyrA D87N+S83Y, parC S80I	Singleton	Not flagged	95.6	Pending
S17	ST307	KL102/O2β	KPC-3	blaKPC-3, plasmid 1 (69,799 bp)	None	OmpK36 D135DGD ins, OmpK35 Y197Ter, gyrA S83I, parC S80I	CL-5	Not flagged	101.8	Pending
S18	ST147	KL64/O2α	NDM-5	blaNDM-5, plasmid 1 (351,373 bp)	mgrB 8,162-bp insertion at codon 45 (reported by Kleborate as c.T132del)	OmpK36 D135DGD ins, OmpK35 R60LfsTer31, gyrA S83I, parC S80I	CL-2	Not flagged	87.0	Pending
S19	ST147	KL64/O2α	NDM-5+OXA-48	blaNDM-5, plasmid 1 (350,811 bp) / blaOXA-48, plasmid 4 (68,942 bp)	pmrB T157P (AMRFinderPlus)	OmpK36 D135DGD ins, OmpK35 R60LfsTer31, gyrA S83I, parC S80I	CL-2	Flagged (0.67)	130.4	Pending
S20	ST147	KL64/O2α	NDM-5+OXA-48	blaNDM-5, plasmid 1 (351,638 bp) / blaOXA-48, plasmid 4 (68,942 bp)	pmrB T157P (AMRFinderPlus)	OmpK36 D135DGD ins, OmpK35 R60LfsTer31, gyrA S83I, parC S80I	CL-2	Flagged (0.67)	88.8	Pending
S21	ST17	KL147/O2β	NDM-7	blaNDM-7, IncX3 element (46,161 bp), not classified as a plasmid by MOB-suite	None	None	CL-4	Not flagged	118.4	Pending
S22	ST147	KL64/O2α	NDM-5+OXA-48	blaNDM-5, plasmid 1 (351,996 bp) / blaOXA-48, plasmid 4 (68,942 bp)	mgrB 8,162-bp insertion at codon 45 (reported by Kleborate as c.T132del)	OmpK36 D135DGD ins, OmpK36 Q313Ter, OmpK35 R60LfsTer31, gyrA S83I, parC S80I	CL-2	Flagged (0.66)	131.6	Pending
S23	ST147	KL64/O2α	KPC-3+NDM-5+OXA-48	blaNDM-5, plasmid 1 (367,492 bp) / blaKPC-3, plasmid 3 (89,256 bp) / blaOXA-48, plasmid 4 (68,942 bp)	None	OmpK36 D135DGD ins, OmpK35 R60LfsTer31, gyrA S83I, parC S80I	Singleton	Flagged (0.67)	115.4	Pending
S24	ST147	KL64/O2α	NDM-5+OXA-48	blaNDM-5, plasmid 1 (367,493 bp) / blaOXA-48, plasmid 3 (68,942 bp)	None	OmpK36 D135DGD ins, OmpK35 R60LfsTer31, gyrA S83I, parC S80I	CL-1	Flagged (0.69)	137.3	Pending
S25	ST147	KL64/O2α	NDM-5+OXA-48	blaNDM-5, plasmid 1 (367,492 bp) / blaOXA-48, plasmid 3 (68,942 bp)	None	OmpK36 D135DGD ins, OmpK35 R60LfsTer31, gyrA S83I, parC S80I, ramR K104RfsTer6	CL-1	Flagged (0.69)	138.5	Pending
S26	ST147	KL64/O2α	NDM-5+OXA-48	blaNDM-5, chromosome-integrated pAA405-like element (492,660 bp), blaOXA-48, pAA002 (68,942 bp)	None	OmpK36 D135DGD ins, OmpK35 R60LfsTer31, gyrA S83I, parC S80I	CL-1	Flagged (0.61)	102.3	Pending

Isolate	ST	K/O locus	Carbapenemase	Carbapenemase location	Colistin-associated alteration	Other selected chromosomal variants	Genetic cluster (≤20 alleles)	GUNC (CSS)	Coverage (×)	SRA / BioSample / GenBank
S27	ST307	KL102/O2β	KPC-3	blaKPC-3, plasmid 1 (89,256 bp)	None	OmpK36 D135DGD ins, OmpK35 Y197Ter, gyrA S83I, parC S80I	CL-5	Not flagged	125.4	Pending
S28	ST307	KL102/O2β	KPC-3	blaKPC-3, plasmid 2 (105,372 bp)	None (mgrB p.Lys43fs, colistin-susceptible)	OmpK36 D135DGD ins, OmpK35 Y197Ter, gyrA S83I, parC S80I, nfsB H128VfsTer6	Singleton	Not flagged	88.0	Pending

ST, sequence type. CSS, clade separation score. Genetic clusters (CL) were defined at ≤20 allele differences. GUNC flags were raised at the family level only. Carbapenemase locations were derived from the assembled replicons. Colistin-associated alterations were identified with Kleborate and AMRFinderPlus. For S14, S18 and S22, the Kleborate call c.T132del corresponds to an 8,162-bp insertion within mgrB identified in the assemblies. The mgrB alterations of S11 and S28 were not accompanied by colistin resistance. These assembly-level findings were not validated with read-level data. Accession numbers (SRA, BioSample, GenBank) are under BioProject PRJNA1538236 and will be added after release.

Supplementary Table S8. Sensitivity of cgMLST-based cluster assignment to the allelic distance threshold in 25 carbapenem-resistant Klebsiella pneumoniae isolates.

Allelic difference threshold	Number of clusters	Sizes of multi-isolate clusters	Singleton isolates
≤6	15	3, 3, 4, 2, 3	10
≤7	14	3, 3, 5, 2, 3	9
≤10	14	3, 3, 5, 2, 3	9
≤15	14	3, 3, 5, 2, 3	9
≤20*	12	3, 3, 2, 5, 5	7
≤25	11	3, 3, 2, 10	7
≤30	11	3, 3, 2, 10	7
≤50	9	3, 4, 12	6

Clusters were defined by single-linkage clustering on pairwise allelic differences of the cgMLST matrix (2,335 loci, 25 isolates). Threshold of ≤20 allelic differences, which was selected before the allelic distance matrix was examined and used in the main analysis. The remaining thresholds were evaluated afterwards as a sensitivity analysis. No pairwise distances fell between 21 and 24 alleles. At ≤7, CL-1, CL-4 and CL-5 remained intact, CL-2 separated into groups of three and two isolates, and CL-3 was not formed. The two clusters of five isolates at ≤20 merged into a single cluster of ten isolates at ≤25.

Supplementary Table S9. GUNC screening results for the 25 carbapenem-resistant Klebsiella pneumoniae genomes.

Isolate	Genes called	Genes mapped	Contigs	GUNC result	Level with max CSS	CSS	Contamination portion	Effective surplus clades	Mean hit identity	Reference representation score	Genes assigned to other families (contigs)
S04	5150	5107	2	Not flagged	Kingdom	0.00	0.00	0.00	0.97	0.97	-
S05	5208	5158	3	Not flagged	Kingdom	0.00	0.00	0.00	0.97	0.96	-
S06	5831	5641	5	Flagged	Family	0.65	0.02	0.05	0.97	0.89	424 (5)
S07	5580	5432	2	Not flagged	Kingdom	0.00	0.00	0.00	0.96	0.94	-
S08	5664	5490	4	Flagged	Family	0.69	0.02	0.04	0.97	0.90	378 (4)
S09	5491	5348	3	Not flagged	Kingdom	0.00	0.00	0.00	0.96	0.94	-
S10	5491	5348	3	Not flagged	Kingdom	0.00	0.00	0.00	0.96	0.94	-
S11	5791	5640	4	Not flagged	Kingdom	0.00	0.00	0.00	0.97	0.94	-
S12	5163	5098	3	Not flagged	Kingdom	0.00	0.00	0.00	0.97	0.96	-
S13	5215	5166	3	Not flagged	Kingdom	0.00	0.00	0.00	0.98	0.97	-
S14	5841	5650	6	Flagged	Family	0.67	0.02	0.05	0.97	0.89	430 (6)
S15	5200	5101	6	Not flagged	Kingdom	0.00	0.00	0.00	0.97	0.95	-
S16	5734	5583	7	Not flagged	Kingdom	0.00	0.00	0.00	0.96	0.93	-
S17	5095	5045	4	Not flagged	Kingdom	0.00	0.00	0.00	0.98	0.97	-
S18	5755	5581	5	Not flagged	Kingdom	0.00	0.00	0.00	0.96	0.93	-
S19	5833	5642	5	Flagged	Family	0.67	0.02	0.05	0.97	0.89	427 (5)
S20	5842	5649	6	Flagged	Family	0.67	0.02	0.05	0.97	0.89	433 (6)
S21	5219	5169	4	Not flagged	Kingdom	0.00	0.00	0.00	0.97	0.96	-
S22	5849	5657	6	Flagged	Family	0.66	0.02	0.05	0.97	0.89	432 (6)
S23	5706	5527	6	Flagged	Family	0.67	0.02	0.05	0.97	0.89	427 (6)
S24	5604	5436	4	Flagged	Family	0.69	0.02	0.05	0.97	0.90	376 (4)
S25	5631	5458	4	Flagged	Family	0.69	0.02	0.05	0.97	0.89	396 (4)
S26	5635	5458	3	Flagged	Family	0.61	0.02	0.05	0.97	0.89	395 (3)
S27	5110	5059	4	Not flagged	Kingdom	0.00	0.00	0.00	0.98	0.97	-
S28	5646	5500	4	Not flagged	Kingdom	0.00	0.00	0.00	0.96	0.94	-

Values are taken from the GUNC maxCSS report of each genome (progenomes reference database). Ten genomes (S06, S08, S14, S19, S20, S22, S23, S24, S25 and S26) were flagged by GUNC, all at the family level only (CSS 0.61–0.69, contamination portion 0.02), and were not flagged at the kingdom, phylum, class, order, genus and species levels. The remaining 15 genomes had CSS 0. The last column gives the number of genes at the family level assigned to families other than the dominant family and the number of contigs carrying them (from the contig assignment files). CSS, clade separation score.

Supplementary Table S10. Antimicrobial resistance elements detected in the 25 isolates, with identity, coverage, functional status and genomic location.

Isolate	AMRFinderPlus element	Subclass	Identity (%)	Coverage (%)	Method	Functional status	Location	ResFinder call, identity/coverage (%)
S04	emrD	Efflux	99.49	100.0	BLASTP	Intact	Chromosome, contig 1	Not reported
S04	oqxB19	Phenicol/Quinolone	100.00	100.0	ALLELEP	Intact	Chromosome, contig 1	OqxB (98.45/100.0)
S04	oqxA10	Phenicol/Quinolone	100.00	100.0	ALLELEP	Intact	Chromosome, contig 1	OqxA (99.15/100.0)
S04	blaSHV-11	Beta-Lactam	100.00	100.0	ALLELEP	Intact	Chromosome, contig 1	blaSHV-89 (99.88/100.0); blaSHV-85 (99.88/100.0); blaSHV-79 (99.88/100.0); blaSHV-56 (99.88/100.0); blaSHV-40 (99.88/100.0)
S04	fosA	Fosfomycin	99.28	99.3	BLASTP	Intact	Chromosome, contig 1	fosA6 (99.76/98.3)
S04	tet(A)	Tetracycline	100.00	100.0	EXACTP	Intact	Plasmid contig, contig 2	tet(A) (100.00/100.0)
S04	qnrB1	Quinolone	100.00	100.0	ALLELEP	Intact	Plasmid contig, contig 2	qnrB1 (100.00/100.0)
S04	dfrA14	Trimethoprim	100.00	100.0	EXACTX	Intact	Plasmid contig, contig 2	dfrA14 (100.00/100.0)
S04	blaCTX-M-15	Cephalosporin	100.00	100.0	ALLELEP	Intact	Plasmid contig, contig 2	blaCTX-M-15 (100.00/100.0)
S04	blaTEM-1	Beta-Lactam	100.00	100.0	ALLELEP	Intact	Plasmid contig, contig 2	blaTEM-1B (100.00/100.0)
S04	aph(6)-Id	Streptomycin	100.00	100.0	EXACTX	Intact	Plasmid contig, contig 2	aph(6)-Id (100.00/100.0); aph(3")-Ib (98.96/35.8)
S04	aph(3")-Ib	Streptomycin	100.00	58.0	PARTIALP	Truncated or disrupted	Plasmid contig, contig 2	aph(3")-Ib (100.00/57.8)
S04	sul2	Sulfonamide	100.00	100.0	EXACTP	Intact	Plasmid contig, contig 2	sul2 (100.00/100.0)
S05	emrD	Efflux	99.49	100.0	BLASTP	Intact	Chromosome, contig 1	Not reported
S05	oqxB19	Phenicol/Quinolone	100.00	100.0	ALLELEP	Intact	Chromosome, contig 1	OqxB (98.45/100.0)
S05	oqxA10	Phenicol/Quinolone	100.00	100.0	ALLELEP	Intact	Chromosome, contig 1	OqxA (99.15/100.0)
S05	blaSHV-11	Beta-Lactam	100.00	100.0	ALLELEP	Intact	Chromosome, contig 1	blaSHV-89 (99.88/100.0); blaSHV-85 (99.88/100.0); blaSHV-79 (99.88/100.0); blaSHV-56 (99.88/100.0); blaSHV-40 (99.88/100.0)
S05	fosA	Fosfomycin	99.28	99.3	BLASTP	Intact	Chromosome, contig 1	fosA6 (99.76/98.3)
S05	tet(A)	Tetracycline	100.00	100.0	EXACTP	Intact	Plasmid contig, contig 2	tet(A) (100.00/100.0)
S05	qnrB1	Quinolone	100.00	100.0	ALLELEP	Intact	Plasmid contig, contig 2	qnrB1 (100.00/100.0)
S05	dfrA14	Trimethoprim	100.00	100.0	EXACTX	Intact	Plasmid contig, contig 2	dfrA14 (100.00/100.0)
S05	blaCTX-M-15	Cephalosporin	100.00	100.0	ALLELEP	Intact	Plasmid contig, contig 2	blaCTX-M-15 (100.00/100.0)
S05	blaTEM-1	Beta-Lactam	100.00	100.0	ALLELEP	Intact	Plasmid contig, contig 2	blaTEM-1B (100.00/100.0)
S05	aph(6)-Id	Streptomycin	100.00	100.0	EXACTX	Intact	Plasmid contig, contig 2	aph(6)-Id (100.00/100.0); aph(3")-Ib (98.96/35.8)
S05	aph(3")-Ib	Streptomycin	100.00	58.0	PARTIALP	Truncated or disrupted	Plasmid contig, contig 2	aph(3")-Ib (100.00/57.8)
S05	sul2	Sulfonamide	100.00	100.0	EXACTP	Intact	Plasmid contig, contig 2	sul2 (100.00/100.0)
S05	ble	Bleomycin	100.00	100.0	EXACTP	Intact	Plasmid contig, contig 3	Not reported
S05	blaNDM-7	Carbapenem	100.00	100.0	ALLELEP	Intact	Plasmid contig, contig 3	blaNDM-7 (100.00/100.0)
S06	emrD	Efflux	99.49	100.0	BLASTP	Intact	Chromosome, contig 1	Not reported
S06	parC_S80I	Quinolone	99.27	98.8	POINTP	Point variant	Chromosome, contig 1	n/a
S06	oqxB	Nitrofurantoin/Phenicol/Quinolone/Tigecycline	99.81	100.0	BLASTP	Intact	Chromosome, contig 1	OqxB (98.54/100.0)

Isolate	AMRFinderPlus element	Subclass	Identity (%)	Coverage (%)	Method	Functional status	Location	ResFinder call, identity/coverage (%)
S06	oqxA	Nitrofurantoin/Phenicol/Quinolone/Tigecycline	100.00	100.0	EXACTP	Intact	Chromosome, contig 1	OqxA (99.49/100.0)
S06	gyrA_S83I	Quinolone	99.77	100.0	POINTP	Point variant	Chromosome, contig 1	n/a
S06	ompK36_D135DGD	Carbapenem	98.92	100.0	POINTP	Point variant	Chromosome, contig 1	n/a
S06	blaSHV-11	Beta-Lactam	100.00	100.0	ALLELEP	Intact	Chromosome, contig 1	blaSHV-11 (99.77/100.0); blaSHV-67 (99.77/100.0)
S06	ompK35_R60LfsTer31	Carbapenem	100.00	100.0	POINTX	Truncated or disrupted	Chromosome, contig 1	n/a
S06	pmrB_T157P	Colistin	99.18	100.0	POINTP	Point variant	Chromosome, contig 1	n/a
S06	fosA	Fosfomycin	99.28	99.3	BLASTP	Intact	Chromosome, contig 1	fosA (98.79/98.3)
S06	catA1	Chloramphenicol	100.00	66.2	PARTIALP	Truncated or disrupted	Plasmid contig, contig 5	catA1 (99.56/68.8)
S06	ble	Bleomycin	100.00	100.0	EXACTP	Intact	Plasmid contig, contig 5	Not reported
S06	blaNDM-5	Carbapenem	100.00	100.0	ALLELEP	Intact	Plasmid contig, contig 5	blaNDM-5 (100.00/100.0)
S06	aph(3')-VI	Amikacin/Kanamycin	100.00	100.0	EXACTP	Intact	Plasmid contig, contig 5	aph(3')-VI (100.00/100.0)
S06	qnrS1	Quinolone	100.00	100.0	ALLELEP	Intact	Plasmid contig, contig 5	qnrS1 (100.00/100.0)
S06	blaCTX-M-15	Cephalosporin	100.00	100.0	ALLELEP	Intact	Plasmid contig, contig 5	blaCTX-M-15 (100.00/100.0)
S06	aac(6')-Ib	Amikacin/Kanamycin/Tobramycin	100.00	100.0	BLASTP	Intact	Plasmid contig, contig 5	aac(6')-Ib (100.00/100.0)
S06	aadA1	Streptomycin	100.00	100.0	EXACTX	Intact	Plasmid contig, contig 5	aadA1 (100.00/100.0)
S06	blaOXA	Beta-Lactam	99.64	100.0	INTERNAL_STOP	Truncated or disrupted	Plasmid contig, contig 5	blaOXA-9 (99.88/100.0)
S06	blaTEM-1	Beta-Lactam	100.00	100.0	ALLELEP	Intact	Plasmid contig, contig 5	blaTEM-1C (99.88/100.0); blaTEM-1B (99.88/100.0)
S06	mph(A)	Azithromycin/Erythromycin/Spiramycin/Telithromycin	100.00	100.0	BLASTP	Intact	Plasmid contig, contig 5	mph(A) (100.00/99.7)
S06	mrx(A)	Azithromycin/Erythromycin/Spiramycin/Telithromycin	100.00	100.0	EXACTP	Intact	Plasmid contig, contig 5	Not reported
S06	sul1	Sulfonamide	100.00	100.0	EXACTP	Intact	Plasmid contig, contig 5	sul1 (100.00/100.0)
S06	dfrA5	Trimethoprim	100.00	100.0	EXACTX	Intact	Plasmid contig, contig 5	dfrA5 (100.00/99.6)
S06	arr-2	Rifamycin	100.00	100.0	ALLELEP	Intact	Plasmid contig, contig 6	ARR-2 (100.00/100.0)
S06	aac(6')-Ib	Gentamicin	100.00	100.0	EXACTP	Intact	Plasmid contig, contig 6	aac(6')-Ib-Hangzhou (99.61/100.0)
S06	rmtF1	Aminoglycoside	100.00	100.0	ALLELEP	Intact	Plasmid contig, contig 6	rmtF (100.00/100.0)
S06	catB	Chloramphenicol	100.00	100.0	EXACTP	Intact	Plasmid contig, contig 6	Not reported
S06	qnrB1	Quinolone	100.00	100.0	ALLELEP	Intact	Plasmid contig, contig 6	qnrB1 (100.00/100.0)
S06	dfrA50	Trimethoprim	98.73	100.0	BLASTP	Intact	Unclassified contig, contig 7	Not reported
S06	blaOXA-48	Carbapenem	100.00	100.0	ALLELEP	Intact	Plasmid contig, contig 8	blaOXA-48 (100.00/100.0)
S06	blaCTX-M-14	Cephalosporin	100.00	100.0	ALLELEP	Intact	Plasmid contig, contig 8	blaCTX-M-14b (100.00/100.0)
S06	aph(3'')-Ib	Streptomycin	100.00	100.0	EXACTP	Intact	Plasmid contig, contig 8	aph(6)-Id (100.00/100.0); aph(3'')-Ib (100.00/99.9)

Isolate	AMRFinderPlus element	Subclass	Identity (%)	Coverage (%)	Method	Functional status	Location	ResFinder call, identity/coverage (%)
S06	aph(6)-Id	Streptomycin	100.00	100.0	EXACTP	Intact	Plasmid contig, contig 8	aph(6)-Id (100.00/100.0); aph(3")-Ib (100.00/99.9)
S07	emrD	Efflux	99.24	100.0	BLASTP	Intact	Chromosome, contig 1	Not reported
S07	parC_S80I	Quinolone	99.12	98.8	POINTP	Point variant	Chromosome, contig 1	n/a
S07	oqxB19	Phenicol/Quinolone	100.00	100.0	ALLELEP	Intact	Chromosome, contig 1	OqxB (98.57/100.0)
S07	oqxA	Nitrofurantoin/Phenicol/Quinolone/Tigecycline	100.00	100.0	EXACTP	Intact	Chromosome, contig 1	OqxA (98.89/100.0)
S07	gyrA_S83I	Quinolone	99.77	100.0	POINTP	Point variant	Chromosome, contig 1	n/a
S07	blaSHV-28	Beta-Lactam	100.00	100.0	ALLELEP	Intact	Chromosome, contig 1	blaSHV-106 (99.88/100.0); blaSHV-28 (99.88/100.0)
S07	fosA	Fosfomycin	100.00	100.0	EXACTP	Intact	Chromosome, contig 1	fosA6 (99.76/100.0)
S07	qnrB1	Quinolone	100.00	100.0	ALLELEP	Intact	Plasmid contig, contig 2	qnrB1 (100.00/100.0)
S07	dfrA14	Trimethoprim	100.00	100.0	EXACTX	Intact	Plasmid contig, contig 2	dfrA14 (100.00/100.0)
S07	blaCTX-M-15	Cephalosporin	100.00	100.0	ALLELEP	Intact	Plasmid contig, contig 2	blaCTX-M-15 (100.00/100.0)
S07	aac(6')-Ib	Amikacin/Kanamycin/Tobramycin	100.00	100.0	BLASTP	Intact	Plasmid contig, contig 2	aac(6')-Ib (100.00/100.0)
S07	aadA1	Streptomycin	100.00	100.0	EXACTX	Intact	Plasmid contig, contig 2	aadA1 (100.00/100.0)
S07	blaOXA	Beta-Lactam	99.64	100.0	INTERNAL_STOP	Truncated or disrupted	Plasmid contig, contig 2	blaOXA-9 (99.88/100.0)
S07	blaTEM-1	Beta-Lactam	100.00	100.0	ALLELEP	Intact	Plasmid contig, contig 2	blaTEM-1B (99.88/100.0); blaTEM-1C (99.88/100.0)
S07	blaOXA-48	Carbapenem	100.00	100.0	ALLELEP	Intact	Plasmid contig, contig 2	blaOXA-48 (100.00/100.0)
S07	blaCTX-M-14	Cephalosporin	100.00	100.0	ALLELEP	Intact	Plasmid contig, contig 2	blaCTX-M-14b (100.00/100.0)
S07	mph(A)	Azithromycin/Erythromycin/Spiramycin/Telithromycin	100.00	100.0	BLASTP	Intact	Plasmid contig, contig 2	mph(A) (100.00/99.7)
S07	mrx(A)	Azithromycin/Erythromycin/Spiramycin/Telithromycin	100.00	100.0	EXACTP	Intact	Plasmid contig, contig 2	Not reported
S07	dfrA5	Trimethoprim	100.00	100.0	EXACTX	Intact	Plasmid contig, contig 2	dfrA5 (100.00/100.0)
S07	sul1	Sulfonamide	100.00	100.0	EXACTP	Intact	Plasmid contig, contig 2	sul1 (100.00/100.0)
S07	catA1	Chloramphenicol	100.00	66.2	PARTIALP	Truncated or disrupted	Plasmid contig, contig 2	catA1 (99.56/68.8)
S07	ble	Bleomycin	100.00	100.0	EXACTP	Intact	Plasmid contig, contig 2	Not reported
S07	blaNDM-5	Carbapenem	100.00	100.0	ALLELEP	Intact	Plasmid contig, contig 2	blaNDM-5 (100.00/100.0)
S07	aph(3')-VI	Amikacin/Kanamycin	100.00	100.0	EXACTP	Intact	Plasmid contig, contig 2	aph(3')-VI (100.00/100.0)
S07	qnrS1	Quinolone	100.00	100.0	ALLELEP	Intact	Plasmid contig, contig 2	qnrS1 (100.00/100.0)
S07	blaCTX-M-15	Cephalosporin	100.00	100.0	ALLELEP	Intact	Plasmid contig, contig 2	blaCTX-M-15 (100.00/100.0)
S07	blaTEM-1	Beta-Lactam	100.00	100.0	ALLELEP	Intact	Plasmid contig, contig 2	blaTEM-1B (100.00/100.0)
S07	aph(6)-Id	Streptomycin	100.00	100.0	EXACTP	Intact	Plasmid contig, contig 2	aph(6)-Id (100.00/100.0); aph(3")-Ib (100.00/100.0)
S07	aph(3")-Ib	Streptomycin	100.00	100.0	EXACTP	Intact	Plasmid contig, contig 2	aph(6)-Id (100.00/100.0); aph(3")-Ib (100.00/100.0)
S07	sul2	Sulfonamide	100.00	100.0	EXACTP	Intact	Plasmid contig, contig 2	sul2 (100.00/100.0)

Isolate	AMRFinderPlus element	Subclass	Identity (%)	Coverage (%)	Method	Functional status	Location	ResFinder call, identity/coverage (%)
S08	emrD	Efflux	99.49	100.0	BLASTP	Intact	Chromosome, contig 1	Not reported
S08	parC_S80I	Quinolone	99.27	98.8	POINTP	Point variant	Chromosome, contig 1	n/a
S08	oqxB	Nitrofurantoin/Phenicol/Quinolone/Tigecycline	99.81	100.0	BLASTP	Intact	Chromosome, contig 1	OqxB (98.54/100.0)
S08	oqxA	Nitrofurantoin/Phenicol/Quinolone/Tigecycline	100.00	100.0	EXACTP	Intact	Chromosome, contig 1	OqxA (99.49/100.0)
S08	gyrA_S83I	Quinolone	99.77	100.0	POINTP	Point variant	Chromosome, contig 1	n/a
S08	ompK36_D135DGD	Carbapenem	98.92	100.0	POINTP	Point variant	Chromosome, contig 1	n/a
S08	blaSHV-11	Beta-Lactam	100.00	100.0	ALLELEP	Intact	Chromosome, contig 1	blaSHV-11 (99.77/100.0); blaSHV-67 (99.77/100.0)
S08	ompK35_R60LfsTer31	Carbapenem	100.00	100.0	POINTX	Truncated or disrupted	Chromosome, contig 1	n/a
S08	fosA	Fosfomycin	99.28	99.3	BLASTP	Intact	Chromosome, contig 1	fosA (98.79/98.3)
S08	catA1	Chloramphenicol	100.00	66.2	PARTIALP	Truncated or disrupted	Plasmid contig, contig 2	catA1 (99.56/68.8)
S08	ble	Bleomycin	100.00	100.0	EXACTP	Intact	Plasmid contig, contig 2	Not reported
S08	blaNDM-5	Carbapenem	100.00	100.0	ALLELEP	Intact	Plasmid contig, contig 2	blaNDM-5 (100.00/100.0)
S08	aph(3')-VI	Amikacin/Kanamycin	100.00	100.0	EXACTP	Intact	Plasmid contig, contig 2	aph(3')-VI (100.00/100.0)
S08	qnrS1	Quinolone	100.00	100.0	ALLELEP	Intact	Plasmid contig, contig 2	qnrS1 (100.00/100.0)
S08	blaCTX-M-15	Cephalosporin	100.00	100.0	ALLELEP	Intact	Plasmid contig, contig 2	blaCTX-M-15 (100.00/100.0)
S08	aac(6')-Ib	Amikacin/Kanamycin/Tobramycin	100.00	100.0	BLASTP	Intact	Plasmid contig, contig 2	aac(6')-Ib (100.00/100.0)
S08	aadA1	Streptomycin	100.00	100.0	EXACTX	Intact	Plasmid contig, contig 2	aadA1 (100.00/100.0)
S08	blaOXA	Beta-Lactam	99.64	100.0	INTERNAL_STOP	Truncated or disrupted	Plasmid contig, contig 2	blaOXA-9 (99.88/100.0)
S08	blaTEM-1	Beta-Lactam	100.00	100.0	ALLELEP	Intact	Plasmid contig, contig 2	blaTEM-1C (99.88/100.0); blaTEM-1B (99.88/100.0)
S08	mph(A)	Azithromycin/Erythromycin/Spiramycin/Telithromycin	100.00	100.0	BLASTP	Intact	Plasmid contig, contig 2	mph(A) (100.00/99.7)
S08	mrx(A)	Azithromycin/Erythromycin/Spiramycin/Telithromycin	100.00	100.0	EXACTP	Intact	Plasmid contig, contig 2	Not reported
S08	sul1	Sulfonamide	100.00	100.0	EXACTP	Intact	Plasmid contig, contig 2	sul1 (100.00/100.0)
S08	dfrA5	Trimethoprim	100.00	100.0	EXACTX	Intact	Plasmid contig, contig 2	dfrA5 (100.00/99.6)
S08	dfrA50	Trimethoprim	98.73	100.0	BLASTP	Intact	Unclassified contig, contig 3	Not reported
S08	blaOXA-48	Carbapenem	100.00	100.0	ALLELEP	Intact	Plasmid contig, contig 4	blaOXA-48 (100.00/100.0)
S08	blaCTX-M-14	Cephalosporin	100.00	100.0	ALLELEP	Intact	Plasmid contig, contig 4	blaCTX-M-14b (100.00/100.0)
S08	aph(3")-Ib	Streptomycin	100.00	100.0	EXACTP	Intact	Plasmid contig, contig 4	aph(6)-Id (100.00/100.0); aph(3")-Ib (100.00/99.9)
S08	aph(6)-Id	Streptomycin	100.00	100.0	EXACTP	Intact	Plasmid contig, contig 4	aph(6)-Id (100.00/100.0); aph(3")-Ib (100.00/99.9)

Isolate	AMRFinderPlus element	Subclass	Identity (%)	Coverage (%)	Method	Functional status	Location	ResFinder call, identity/coverage (%)
S09	emrD	Efflux	99.49	100.0	BLASTP	Intact	Chromosome, contig 1	Not reported
S09	parC_S80I	Quinolone	99.27	98.8	POINTP	Point variant	Chromosome, contig 1	n/a
S09	oqxB	Nitrofurantoin/Phenicol/Quinolone/Tigecycline	99.81	100.0	BLASTP	Intact	Chromosome, contig 1	OqxB (98.54/100.0)
S09	oqxA	Nitrofurantoin/Phenicol/Quinolone/Tigecycline	100.00	100.0	EXACTP	Intact	Chromosome, contig 1	OqxA (99.49/100.0)
S09	gyrA_S83I	Quinolone	99.77	100.0	POINTP	Point variant	Chromosome, contig 1	n/a
S09	ompK36_D135DGD	Carbapenem	98.92	100.0	POINTP	Point variant	Chromosome, contig 1	n/a
S09	blaSHV-11	Beta-Lactam	100.00	100.0	ALLELEP	Intact	Chromosome, contig 1	blaSHV-11 (99.77/100.0); blaSHV-67 (99.77/100.0)
S09	ompK35_R60LfsTer31	Carbapenem	100.00	100.0	POINTX	Truncated or disrupted	Chromosome, contig 1	n/a
S09	fosA	Fosfomycin	99.28	99.3	BLASTP	Intact	Chromosome, contig 1	fosA (98.79/98.3)
S09	catA1	Chloramphenicol	100.00	66.2	PARTIALP	Truncated or disrupted	Plasmid contig, contig 2	catA1 (99.56/68.8)
S09	ble	Bleomycin	100.00	100.0	EXACTP	Intact	Plasmid contig, contig 2	Not reported
S09	blaNDM-5	Carbapenem	100.00	100.0	ALLELEP	Intact	Plasmid contig, contig 2	blaNDM-5 (100.00/100.0)
S09	aph(3')-VI	Amikacin/Kanamycin	100.00	100.0	EXACTP	Intact	Plasmid contig, contig 2	aph(3')-VI (100.00/100.0)
S09	qnrS1	Quinolone	100.00	100.0	ALLELEP	Intact	Plasmid contig, contig 2	qnrS1 (100.00/100.0)
S09	blaCTX-M-15	Cephalosporin	100.00	100.0	ALLELEP	Intact	Plasmid contig, contig 2	blaCTX-M-15 (100.00/100.0)
S09	aac(6')-Ib	Amikacin/Kanamycin/Tobramycin	100.00	100.0	BLASTP	Intact	Plasmid contig, contig 2	aac(6')-Ib (100.00/100.0)
S09	aadA1	Streptomycin	100.00	100.0	EXACTX	Intact	Plasmid contig, contig 2	aadA1 (100.00/100.0)
S09	blaOXA	Beta-Lactam	99.64	100.0	INTERNAL_STOP	Truncated or disrupted	Plasmid contig, contig 2	blaOXA-9 (99.88/100.0)
S09	blaTEM-1	Beta-Lactam	100.00	100.0	ALLELEP	Intact	Plasmid contig, contig 2	blaTEM-1C (99.88/100.0); blaTEM-1B (99.88/100.0)
S09	arr-2	Rifamycin	100.00	100.0	ALLELEP	Intact	Plasmid contig, contig 2	ARR-2 (100.00/100.0)
S09	aac(6')-Ib	Gentamicin	100.00	100.0	EXACTP	Intact	Plasmid contig, contig 2	aac(6')-Ib-Hangzhou (99.61/100.0)
S09	rmtF1	Aminoglycoside	100.00	100.0	ALLELEP	Intact	Plasmid contig, contig 2	rmtF (100.00/100.0)
S09	catB	Chloramphenicol	100.00	100.0	EXACTP	Intact	Plasmid contig, contig 2	Not reported
S09	dfrA5	Trimethoprim	100.00	100.0	EXACTX	Intact	Plasmid contig, contig 2	dfrA5 (100.00/100.0)
S09	sul1	Sulfonamide	100.00	100.0	EXACTP	Intact	Plasmid contig, contig 2	sul1 (100.00/100.0)
S09	mrx(A)	Azithromycin/Erythromycin/Spiramycin/Telithromycin	100.00	100.0	EXACTP	Intact	Plasmid contig, contig 2	Not reported
S09	mph(A)	Azithromycin/Erythromycin/Spiramycin/Telithromycin	100.00	100.0	BLASTP	Intact	Plasmid contig, contig 2	mph(A) (100.00/99.7)
S09	aph(3')-Ia	Kanamycin	100.00	100.0	EXACTP	Intact	Plasmid contig, contig 2	aph(3')-Ia (99.88/100.0)
S09	blaOXA-48	Carbapenem	100.00	100.0	ALLELEP	Intact	Plasmid contig, contig 3	blaOXA-48 (100.00/100.0)

Isolate	AMRFinderPlus element	Subclass	Identity (%)	Coverage (%)	Method	Functional status	Location	ResFinder call, identity/coverage (%)
S09	blaCTX-M-14	Cephalosporin	100.00	100.0	ALLELEP	Intact	Plasmid contig, contig 3	blaCTX-M-14b (100.00/100.0)
S09	aph(3")-Ib	Streptomycin	100.00	100.0	EXACTP	Intact	Plasmid contig, contig 3	aph(6)-Id (100.00/100.0); aph(3")-Ib (100.00/99.9)
S09	aph(6)-Id	Streptomycin	100.00	100.0	EXACTP	Intact	Plasmid contig, contig 3	aph(6)-Id (100.00/100.0); aph(3")-Ib (100.00/99.9)
S10	emrD	Efflux	99.49	100.0	BLASTP	Intact	Chromosome, contig 1	Not reported
S10	parC_S80I	Quinolone	99.27	98.8	POINTP	Point variant	Chromosome, contig 1	n/a
S10	oqxB	Nitrofurantoin/Phenicol/Quinolone/Tigecycline	99.81	100.0	BLASTP	Intact	Chromosome, contig 1	OqxB (98.54/100.0)
S10	oqxA	Nitrofurantoin/Phenicol/Quinolone/Tigecycline	100.00	100.0	EXACTP	Intact	Chromosome, contig 1	OqxA (99.49/100.0)
S10	gyrA_S83I	Quinolone	99.77	100.0	POINTP	Point variant	Chromosome, contig 1	n/a
S10	ompK36_D135DGD	Carbapenem	98.92	100.0	POINTP	Point variant	Chromosome, contig 1	n/a
S10	blaSHV-11	Beta-Lactam	100.00	100.0	ALLELEP	Intact	Chromosome, contig 1	blaSHV-11 (99.77/100.0); blaSHV-67 (99.77/100.0)
S10	ompK35_R60LfsTer31	Carbapenem	100.00	100.0	POINTX	Truncated or disrupted	Chromosome, contig 1	n/a
S10	fosA	Fosfomycin	99.28	99.3	BLASTP	Intact	Chromosome, contig 1	fosA (98.79/98.3)
S10	catA1	Chloramphenicol	100.00	66.2	PARTIALP	Truncated or disrupted	Plasmid contig, contig 2	catA1 (99.56/68.8)
S10	ble	Bleomycin	100.00	100.0	EXACTP	Intact	Plasmid contig, contig 2	Not reported
S10	blaNDM-5	Carbapenem	100.00	100.0	ALLELEP	Intact	Plasmid contig, contig 2	blaNDM-5 (100.00/100.0)
S10	aph(3')-VI	Amikacin/Kanamycin	100.00	100.0	EXACTP	Intact	Plasmid contig, contig 2	aph(3')-VI (100.00/100.0)
S10	qnrS1	Quinolone	100.00	100.0	ALLELEP	Intact	Plasmid contig, contig 2	qnrS1 (100.00/100.0)
S10	blaCTX-M-15	Cephalosporin	100.00	100.0	ALLELEP	Intact	Plasmid contig, contig 2	blaCTX-M-15 (100.00/100.0)
S10	aac(6')-Ib	Amikacin/Kanamycin/Tobramycin	100.00	100.0	BLASTP	Intact	Plasmid contig, contig 2	aac(6')-Ib (100.00/100.0)
S10	aadA1	Streptomycin	100.00	100.0	EXACTX	Intact	Plasmid contig, contig 2	aadA1 (100.00/100.0)
S10	blaOXA	Beta-Lactam	99.64	100.0	INTERNAL_STOP	Truncated or disrupted	Plasmid contig, contig 2	blaOXA-9 (99.88/100.0)
S10	blaTEM-1	Beta-Lactam	100.00	100.0	ALLELEP	Intact	Plasmid contig, contig 2	blaTEM-1C (99.88/100.0); blaTEM-1B (99.88/100.0)
S10	arr-2	Rifamycin	100.00	100.0	ALLELEP	Intact	Plasmid contig, contig 2	ARR-2 (100.00/100.0)
S10	aac(6')-Ib	Gentamicin	100.00	100.0	EXACTP	Intact	Plasmid contig, contig 2	aac(6')-Ib-Hangzhou (99.61/100.0)
S10	rmtF1	Aminoglycoside	100.00	100.0	ALLELEP	Intact	Plasmid contig, contig 2	rmtF (100.00/100.0)
S10	catB	Chloramphenicol	100.00	100.0	EXACTP	Intact	Plasmid contig, contig 2	Not reported
S10	dfrA5	Trimethoprim	100.00	100.0	EXACTX	Intact	Plasmid contig, contig 2	dfrA5 (100.00/100.0)
S10	sul1	Sulfonamide	100.00	100.0	EXACTP	Intact	Plasmid contig, contig 2	sul1 (100.00/100.0)
S10	mrx(A)	Azithromycin/Erythromycin/Spiramycin/Telithromycin	100.00	100.0	EXACTP	Intact	Plasmid contig, contig 2	Not reported

Isolate	AMRFinderPlus element	Subclass	Identity (%)	Coverage (%)	Method	Functional status	Location	ResFinder call, identity/coverage (%)
S10	mph(A)	Azithromycin/Erythromycin/Spiramycin/Telithromycin	100.00	100.0	BLASTP	Intact	Plasmid contig, contig 2	mph(A) (100.00/99.7)
S10	aph(3')-Ia	Kanamycin	100.00	100.0	EXACTP	Intact	Plasmid contig, contig 2	aph(3')-Ia (99.88/100.0)
S10	blaOXA-48	Carbapenem	100.00	100.0	ALLELEP	Intact	Plasmid contig, contig 3	blaOXA-48 (100.00/100.0)
S10	blaCTX-M-14	Cephalosporin	100.00	100.0	ALLELEP	Intact	Plasmid contig, contig 3	blaCTX-M-14b (100.00/100.0)
S10	aph(3")-Ib	Streptomycin	100.00	100.0	EXACTP	Intact	Plasmid contig, contig 3	aph(6)-Id (100.00/100.0); aph(3")-Ib (100.00/99.9)
S10	aph(6)-Id	Streptomycin	100.00	100.0	EXACTP	Intact	Plasmid contig, contig 3	aph(6)-Id (100.00/100.0); aph(3")-Ib (100.00/99.9)
S11	emrD	Efflux	99.24	100.0	BLASTP	Intact	Chromosome, contig 1	Not reported
S11	blaCTX-M-14	Cephalosporin	100.00	100.0	ALLELEP	Intact	Chromosome, contig 1	blaCTX-M-14 (100.00/100.0)
S11	parC_S80I	Quinolone	99.41	98.8	POINTP	Point variant	Chromosome, contig 1	n/a
S11	oqxA	Nitrofurantoin/Phenicol/Quinolone/Tigecycline	100.00	100.0	EXACTP	Intact	Chromosome, contig 1	OqxA (100.00/100.0)
S11	oqxB	Nitrofurantoin/Phenicol/Quinolone/Tigecycline	100.00	100.0	EXACTP	Intact	Chromosome, contig 1	OqxB (100.00/100.0)
S11	gyrA_S83I	Quinolone	99.77	100.0	POINTP	Point variant	Chromosome, contig 1	n/a
S11	ompK36_D135DGD	Carbapenem	99.46	100.0	POINTP	Point variant	Chromosome, contig 1	n/a
S11	blaSHV-11	Beta-Lactam	100.00	100.0	ALLELEP	Intact	Chromosome, contig 1	blaSHV-182 (99.88/100.0); blaSHV-159 (99.88/100.0); blaSHV-158 (99.88/100.0)
S11	fosA	Fosfomycin	98.56	100.0	BLASTP	Intact	Chromosome, contig 1	fosA6 (98.81/100.0)
S11	catA1	Chloramphenicol	100.00	66.2	PARTIALP	Truncated or disrupted	Plasmid contig, contig 2	catA1 (99.56/68.8)
S11	ble	Bleomycin	100.00	100.0	EXACTP	Intact	Plasmid contig, contig 2	Not reported
S11	blaNDM-5	Carbapenem	100.00	100.0	ALLELEP	Intact	Plasmid contig, contig 2	blaNDM-5 (100.00/100.0)
S11	aph(3')-VI	Amikacin/Kanamycin	100.00	100.0	EXACTP	Intact	Plasmid contig, contig 2	aph(3')-VI (100.00/100.0)
S11	qnrS1	Quinolone	100.00	100.0	ALLELEP	Intact	Plasmid contig, contig 2	qnrS1 (100.00/100.0)
S11	blaCTX-M-15	Cephalosporin	100.00	100.0	ALLELEP	Intact	Plasmid contig, contig 2	blaCTX-M-15 (100.00/100.0)
S11	aac(6')-Ib	Amikacin/Kanamycin/Tobramycin	100.00	100.0	BLASTP	Intact	Plasmid contig, contig 2	aac(6')-Ib (100.00/100.0)
S11	aadA1	Streptomycin	100.00	100.0	EXACTX	Intact	Plasmid contig, contig 2	aadA1 (100.00/100.0)
S11	blaOXA	Beta-Lactam	99.64	100.0	INTERNAL_STOP	Truncated or disrupted	Plasmid contig, contig 2	blaOXA-9 (99.88/100.0)
S11	blaTEM-1	Beta-Lactam	100.00	100.0	ALLELEP	Intact	Plasmid contig, contig 2	blaTEM-1C (99.88/100.0); blaTEM-1B (99.88/100.0)
S11	aph(3')-Ia	Kanamycin	100.00	100.0	EXACTP	Intact	Plasmid contig, contig 2	aph(3')-Ia (99.88/100.0)
S11	mph(A)	Azithromycin/Erythromycin/Spiramycin/Telithromycin	100.00	100.0	BLASTP	Intact	Plasmid contig, contig 2	mph(A) (100.00/99.7)

Isolate	AMRFinderPlus element	Subclass	Identity (%)	Coverage (%)	Method	Functional status	Location	ResFinder call, identity/coverage (%)
S11	mrx(A)	Azithromycin/Erythromycin/Spiramycin/Telithromycin	100.00	100.0	EXACTP	Intact	Plasmid contig, contig 2	Not reported
S11	dfrA5	Trimethoprim	100.00	100.0	EXACTX	Intact	Plasmid contig, contig 2	dfrA5 (100.00/100.0)
S11	sul1	Sulfonamide	100.00	100.0	EXACTP	Intact	Plasmid contig, contig 2	sul1 (100.00/100.0)
S11	qnrS1	Quinolone	100.00	100.0	ALLELEP	Intact	Plasmid contig, contig 3	qnrS1 (100.00/100.0)
S11	blaLAP-2	Beta-Lactam	100.00	100.0	ALLELEP	Intact	Plasmid contig, contig 3	blaLAP-2 (100.00/100.0)
S11	blaOXA-48	Carbapenem	100.00	100.0	ALLELEP	Intact	Plasmid contig, contig 4	blaOXA-48 (100.00/100.0)
S12	emrD	Efflux	99.49	100.0	BLASTP	Intact	Chromosome, contig 1	Not reported
S12	parC_E84K	Quinolone	99.27	98.8	POINTP	Point variant	Chromosome, contig 1	n/a
S12	oqxB	Nitrofurantoin/Phenicol/Quinolone/Tigecycline	99.90	100.0	BLASTP	Intact	Chromosome, contig 1	OqxB (98.86/100.0)
S12	oqxA	Nitrofurantoin/Phenicol/Quinolone/Tigecycline	100.00	100.0	EXACTP	Intact	Chromosome, contig 1	OqxA (99.15/100.0)
S12	gyrA_D87N	Quinolone	99.66	100.0	POINTP	Point variant	Chromosome, contig 1	n/a
S12	gyrA_S83F	Quinolone	99.66	100.0	POINTP	Point variant	Chromosome, contig 1	n/a
S12	ompK36_D135DGD	Carbapenem	94.05	100.0	POINTP	Point variant	Chromosome, contig 1	n/a
S12	blaSHV-1	Beta-Lactam	100.00	100.0	ALLELEP	Intact	Chromosome, contig 1	blaSHV-199 (99.88/100.0); blaSHV-194 (99.88/100.0); blaSHV-179 (99.88/100.0); blaSHV-145 (99.88/100.0); blaSHV-98 (99.88/100.0); blaSHV-78 (99.88/100.0); blaSHV-26 (99.88/100.0)
S12	ompK35_W79Ter	Carbapenem	99.72	100.0	POINTX	Truncated or disrupted	Chromosome, contig 1	n/a
S12	blaCTX-M-15	Cephalosporin	100.00	100.0	ALLELEP	Intact	Chromosome, contig 1	blaCTX-M-15 (100.00/100.0)
S12	blaCTX-M-15	Cephalosporin	100.00	100.0	ALLELEP	Intact	Chromosome, contig 1	blaCTX-M-15 (100.00/100.0)
S12	fosA	Fosfomycin	97.10	99.3	BLASTP	Intact	Chromosome, contig 1	fosA5 (95.00/99.8)
S12	blaOXA-48	Carbapenem	100.00	100.0	ALLELEP	Intact	Plasmid contig, contig 3	blaOXA-48 (100.00/100.0)
S13	emrD	Efflux	99.24	100.0	BLASTP	Intact	Chromosome, contig 1	Not reported
S13	parC_S80I	Quinolone	99.27	98.8	POINTP	Point variant	Chromosome, contig 1	n/a
S13	oqxB19	Phenicol/Quinolone	100.00	100.0	ALLELEP	Intact	Chromosome, contig 1	OqxB (98.57/100.0)
S13	oqxA	Nitrofurantoin/Phenicol/Quinolone/Tigecycline	100.00	100.0	EXACTP	Intact	Chromosome, contig 1	OqxA (98.89/100.0)
S13	gyrA_S83I	Quinolone	99.77	100.0	POINTP	Point variant	Chromosome, contig 1	n/a
S13	ompK36_D135DGD	Carbapenem	92.29	100.0	POINTP	Point variant	Chromosome, contig 1	n/a
S13	blaSHV-28	Beta-Lactam	100.00	100.0	ALLELEP	Intact	Chromosome, contig 1	blaSHV-106 (99.88/100.0); blaSHV-28 (99.88/100.0)
S13	ompK35_Y197Ter	Carbapenem	99.72	100.0	POINTX	Truncated or disrupted	Chromosome, contig 1	n/a
S13	fosA	Fosfomycin	100.00	100.0	EXACTP	Intact	Chromosome, contig 1	fosA6 (99.76/100.0)
S13	qnrB1	Quinolone	100.00	100.0	ALLELEP	Intact	Plasmid contig, contig 2	qnrB1 (100.00/100.0)

Isolate	AMRFinderPlus element	Subclass	Identity (%)	Coverage (%)	Method	Functional status	Location	ResFinder call, identity/coverage (%)
S13	blaKPC-3	Carbapenem	100.00	100.0	ALLELEP	Intact	Plasmid contig, contig 2	blaKPC-3 (100.00/100.0)
S13	dfrA14	Trimethoprim	100.00	100.0	EXACTX	Intact	Plasmid contig, contig 2	dfrA14 (100.00/100.0)
S13	catB3	Chloramphenicol	100.00	70.0	PARTIALP	Truncated or disrupted	Plasmid contig, contig 2	catB3 (100.00/69.8)
S13	blaOXA-1	Cephalosporin	100.00	100.0	ALLELEP	Intact	Plasmid contig, contig 2	blaOXA-1 (100.00/100.0)
S13	aac(6')-Ib	Amikacin/Kanamycin/Tobramycin	100.00	100.0	BLASTP	Intact	Plasmid contig, contig 2	aac(6')-Ib-cr (99.83/100.0)
S14	emrD	Efflux	99.49	100.0	BLASTP	Intact	Chromosome, contig 1	Not reported
S14	parC_S80I	Quinolone	99.27	98.8	POINTP	Point variant	Chromosome, contig 1	n/a
S14	oqxB	Nitrofurantoin/Phenicol/Quinolone/Tigecycline	99.81	100.0	BLASTP	Intact	Chromosome, contig 1	OqxB (98.54/100.0)
S14	oqxA	Nitrofurantoin/Phenicol/Quinolone/Tigecycline	100.00	100.0	EXACTP	Intact	Chromosome, contig 1	OqxA (99.49/100.0)
S14	gyrA_S83I	Quinolone	99.77	100.0	POINTP	Point variant	Chromosome, contig 1	n/a
S14	ompK36_D135DGD	Carbapenem	98.92	100.0	POINTP	Point variant	Chromosome, contig 1	n/a
S14	blaSHV-11	Beta-Lactam	100.00	100.0	ALLELEP	Intact	Chromosome, contig 1	blaSHV-11 (99.77/100.0); blaSHV-67 (99.77/100.0)
S14	ompK35_R60LfsTer31	Carbapenem	100.00	100.0	POINTX	Truncated or disrupted	Chromosome, contig 1	n/a
S14	fosA	Fosfomycin	99.28	99.3	BLASTP	Intact	Chromosome, contig 1	fosA (98.79/98.3)
S14	catA1	Chloramphenicol	100.00	66.2	PARTIALP	Truncated or disrupted	Plasmid contig, contig 2	catA1 (99.56/68.8)
S14	ble	Bleomycin	100.00	100.0	EXACTP	Intact	Plasmid contig, contig 2	Not reported
S14	blaNDM-5	Carbapenem	100.00	100.0	ALLELEP	Intact	Plasmid contig, contig 2	blaNDM-5 (100.00/100.0)
S14	aph(3')-VI	Amikacin/Kanamycin	100.00	100.0	EXACTP	Intact	Plasmid contig, contig 2	aph(3')-VI (100.00/100.0)
S14	qnrS1	Quinolone	100.00	100.0	ALLELEP	Intact	Plasmid contig, contig 2	qnrS1 (100.00/100.0)
S14	blaCTX-M-15	Cephalosporin	100.00	100.0	ALLELEP	Intact	Plasmid contig, contig 2	blaCTX-M-15 (100.00/100.0)
S14	aac(6')-Ib	Amikacin/Kanamycin/Tobramycin	100.00	100.0	BLASTP	Intact	Plasmid contig, contig 2	aac(6')-Ib (100.00/100.0)
S14	aadA1	Streptomycin	100.00	100.0	EXACTX	Intact	Plasmid contig, contig 2	aadA1 (100.00/100.0)
S14	blaOXA	Beta-Lactam	99.64	100.0	INTERNAL_STOP	Truncated or disrupted	Plasmid contig, contig 2	blaOXA-9 (99.88/100.0)
S14	blaTEM-1	Beta-Lactam	100.00	100.0	ALLELEP	Intact	Plasmid contig, contig 2	blaTEM-1C (99.88/100.0); blaTEM-1B (99.88/100.0)
S14	mph(A)	Azithromycin/Erythromycin/Spiramycin/Telithromycin	100.00	100.0	BLASTP	Intact	Plasmid contig, contig 2	mph(A) (100.00/99.7)
S14	mrx(A)	Azithromycin/Erythromycin/Spiramycin/Telithromycin	100.00	100.0	EXACTP	Intact	Plasmid contig, contig 2	Not reported
S14	sul1	Sulfonamide	100.00	100.0	EXACTP	Intact	Plasmid contig, contig 2	sul1 (100.00/100.0)

Isolate	AMRFinderPlus element	Subclass	Identity (%)	Coverage (%)	Method	Functional status	Location	ResFinder call, identity/coverage (%)
S14	dfrA5	Trimethoprim	100.00	100.0	EXACTX	Intact	Plasmid contig, contig 2	dfrA5 (100.00/100.0)
S14	catB	Chloramphenicol	100.00	100.0	EXACTP	Intact	Plasmid contig, contig 3	Not reported
S14	rmtF1	Aminoglycoside	100.00	100.0	ALLELEP	Intact	Plasmid contig, contig 3	rmtF (100.00/100.0)
S14	aac(6')-Ib	Gentamicin	100.00	100.0	EXACTP	Intact	Plasmid contig, contig 3	aac(6')-Ib-Hangzhou (99.61/100.0)
S14	arr-2	Rifamycin	100.00	100.0	ALLELEP	Intact	Plasmid contig, contig 3	ARR-2 (100.00/100.0)
S14	qnrB1	Quinolone	100.00	100.0	ALLELEP	Intact	Plasmid contig, contig 3	qnrB1 (100.00/100.0)
S14	dfrA50	Trimethoprim	98.73	100.0	BLASTP	Intact	Unclassified contig, contig 4	Not reported
S14	blaOXA-48	Carbapenem	100.00	100.0	ALLELEP	Intact	Plasmid contig, contig 5	blaOXA-48 (100.00/100.0)
S14	blaCTX-M-14	Cephalosporin	100.00	100.0	ALLELEP	Intact	Plasmid contig, contig 5	blaCTX-M-14b (100.00/100.0)
S14	aph(3")-Ib	Streptomycin	100.00	100.0	EXACTP	Intact	Plasmid contig, contig 5	aph(6)-Id (100.00/100.0); aph(3")-Ib (100.00/99.9)
S14	aph(6)-Id	Streptomycin	100.00	100.0	EXACTP	Intact	Plasmid contig, contig 5	aph(6)-Id (100.00/100.0); aph(3")-Ib (100.00/99.9)
S15	emrD	Efflux	99.24	100.0	BLASTP	Intact	Chromosome, contig 1	Not reported
S15	parC_S80I	Quinolone	99.27	98.8	POINTP	Point variant	Chromosome, contig 1	n/a
S15	oqxB19	Phenicol/Quinolone	100.00	100.0	ALLELEP	Intact	Chromosome, contig 1	OqxB (98.57/100.0)
S15	oqxA	Nitrofurantoin/Phenicol/Quinolone/Tigecycline	100.00	100.0	EXACTP	Intact	Chromosome, contig 1	OqxA (98.89/100.0)
S15	gyrA_S83I	Quinolone	99.77	100.0	POINTP	Point variant	Chromosome, contig 1	n/a
S15	ompK36_D135DGD	Carbapenem	92.29	100.0	POINTP	Point variant	Chromosome, contig 1	n/a
S15	blaSHV-28	Beta-Lactam	100.00	100.0	ALLELEP	Intact	Chromosome, contig 1	blaSHV-106 (99.88/100.0); blaSHV-28 (99.88/100.0)
S15	ompK35_Y197Ter	Carbapenem	99.72	100.0	POINTX	Truncated or disrupted	Chromosome, contig 1	n/a
S15	fosA	Fosfomycin	100.00	100.0	EXACTP	Intact	Chromosome, contig 1	fosA6 (99.76/100.0)
S15	blaKPC-3	Carbapenem	100.00	100.0	ALLELEP	Intact	Plasmid contig, contig 3	blaKPC-3 (100.00/100.0)
S15	dfrA14	Trimethoprim	100.00	100.0	EXACTX	Intact	Plasmid contig, contig 3	dfrA14 (100.00/100.0)
S16	emrD	Efflux	99.49	100.0	BLASTP	Intact	Chromosome, contig 1	Not reported
S16	parC_S80I	Quinolone	99.27	98.8	POINTP	Point variant	Chromosome, contig 1	n/a
S16	gyrA_D87N	Quinolone	99.66	100.0	POINTP	Point variant	Chromosome, contig 1	n/a
S16	gyrA_S83Y	Quinolone	99.66	100.0	POINTP	Point variant	Chromosome, contig 1	n/a
S16	blaCTX-M-15	Cephalosporin	100.00	100.0	ALLELEP	Intact	Chromosome, contig 1	blaCTX-M-15 (100.00/100.0)
S16	blaSHV-1	Beta-Lactam	100.00	100.0	ALLELEP	Intact	Chromosome, contig 1	blaSHV-106 (99.77/100.0); blaSHV-28 (99.77/100.0)
S16	ompK35_G62AfsTer1	Carbapenem	100.00	100.0	POINTX	Truncated or disrupted	Chromosome, contig 1	n/a
S16	fosA	Fosfomycin	98.56	100.0	BLASTP	Intact	Chromosome, contig 1	fosA (96.43/100.0)
S16	sul1	Sulfonamide	99.64	100.0	BLASTP	Intact	Plasmid contig, contig 3	sul1 (99.88/100.0)
S16	rmtC	Aminoglycoside	100.00	100.0	EXACTX	Intact	Plasmid contig, contig 3	rmtC (100.00/100.0)
S16	blaNDM-1	Carbapenem	100.00	100.0	ALLELEP	Intact	Plasmid contig, contig 3	blaNDM-1 (100.00/100.0)
S16	ble	Bleomycin	100.00	100.0	EXACTP	Intact	Plasmid contig, contig 3	Not reported
S16	catB3	Chloramphenicol	100.00	70.0	PARTIALP	Truncated or disrupted	Plasmid contig, contig 4	catB3 (100.00/69.8)

Isolate	AMRFinderPlus element	Subclass	Identity (%)	Coverage (%)	Method	Functional status	Location	ResFinder call, identity/coverage (%)
S16	blaOXA-1	Cephalosporin	100.00	100.0	ALLELEP	Intact	Plasmid contig, contig 4	blaOXA-1 (100.00/100.0)
S16	aac(6')-Ib-cr5	Amikacin/Kanamycin/Quinolone/Tobramycin	100.00	100.0	ALLELEX	Intact	Plasmid contig, contig 4	aac(6')-Ib-cr (100.00/100.0)
S16	blaTEM-1	Beta-Lactam	100.00	100.0	ALLELEP	Intact	Plasmid contig, contig 4	blaTEM-1A (100.00/100.0)
S16	aac(6')-Ib-cr5	Amikacin/Kanamycin/Quinolone/Tobramycin	100.00	100.0	ALLELEX	Intact	Plasmid contig, contig 4	aac(6')-Ib-cr (100.00/100.0)
S16	arr-3	Rifamycin	100.00	100.0	EXACTP	Intact	Plasmid contig, contig 4	ARR-3 (100.00/100.0)
S16	sul1	Sulfonamide	100.00	100.0	EXACTP	Intact	Plasmid contig, contig 4	sul1 (100.00/100.0)
S16	blaOXA-48	Carbapenem	100.00	100.0	ALLELEP	Intact	Plasmid contig, contig 5	blaOXA-48 (100.00/100.0)
S17	emrD	Efflux	99.24	100.0	BLASTP	Intact	Chromosome, contig 1	Not reported
S17	parC_S80I	Quinolone	99.27	98.8	POINTP	Point variant	Chromosome, contig 1	n/a
S17	oqxB19	Phenicol/Quinolone	100.00	100.0	ALLELEP	Intact	Chromosome, contig 1	OqxB (98.57/100.0)
S17	oqxA	Nitrofurantoin/Phenicol/Quinolone/Tigecycline	100.00	100.0	EXACTP	Intact	Chromosome, contig 1	OqxA (98.89/100.0)
S17	gyrA_S83I	Quinolone	99.77	100.0	POINTP	Point variant	Chromosome, contig 1	n/a
S17	ompK36_D135DGD	Carbapenem	92.29	100.0	POINTP	Point variant	Chromosome, contig 1	n/a
S17	blaSHV-28	Beta-Lactam	100.00	100.0	ALLELEP	Intact	Chromosome, contig 1	blaSHV-106 (99.88/100.0); blaSHV-28 (99.88/100.0)
S17	ompK35_Y197Ter	Carbapenem	99.72	100.0	POINTX	Truncated or disrupted	Chromosome, contig 1	n/a
S17	fosA	Fosfomycin	100.00	100.0	EXACTP	Intact	Chromosome, contig 1	fosA6 (99.76/100.0)
S17	blaKPC-3	Carbapenem	100.00	100.0	ALLELEP	Intact	Plasmid contig, contig 2	blaKPC-3 (100.00/100.0)
S17	dfrA14	Trimethoprim	100.00	100.0	EXACTX	Intact	Plasmid contig, contig 2	dfrA14 (100.00/100.0)
S18	emrD	Efflux	99.49	100.0	BLASTP	Intact	Chromosome, contig 1	Not reported
S18	parC_S80I	Quinolone	99.27	98.8	POINTP	Point variant	Chromosome, contig 1	n/a
S18	oqxB	Nitrofurantoin/Phenicol/Quinolone/Tigecycline	99.81	100.0	BLASTP	Intact	Chromosome, contig 1	OqxB (98.54/100.0)
S18	oqxA	Nitrofurantoin/Phenicol/Quinolone/Tigecycline	100.00	100.0	EXACTP	Intact	Chromosome, contig 1	OqxA (99.49/100.0)
S18	gyrA_S83I	Quinolone	99.77	100.0	POINTP	Point variant	Chromosome, contig 1	n/a
S18	ompK36_D135DGD	Carbapenem	98.92	100.0	POINTP	Point variant	Chromosome, contig 1	n/a
S18	blaSHV-11	Beta-Lactam	100.00	100.0	ALLELEP	Intact	Chromosome, contig 1	blaSHV-11 (99.77/100.0); blaSHV-67 (99.77/100.0)
S18	ompK35_R60LfsTer31	Carbapenem	100.00	100.0	POINTX	Truncated or disrupted	Chromosome, contig 1	n/a
S18	fosA	Fosfomycin	99.28	99.3	BLASTP	Intact	Chromosome, contig 1	fosA (98.79/98.3)
S18	catA1	Chloramphenicol	100.00	66.2	PARTIALP	Truncated or disrupted	Plasmid contig, contig 2	catA1 (99.56/68.8)

Isolate	AMRFinderPlus element	Subclass	Identity (%)	Coverage (%)	Method	Functional status	Location	ResFinder call, identity/coverage (%)
S18	blaTEM-1	Beta-Lactam	100.00	100.0	ALLELEP	Intact	Plasmid contig, contig 2	blaTEM-1C (99.88/100.0); blaTEM-1B (99.88/100.0)
S18	blaOXA	Beta-Lactam	99.64	100.0	INTERNAL_STOP	Truncated or disrupted	Plasmid contig, contig 2	blaOXA-9 (99.88/100.0)
S18	aadA1	Streptomycin	100.00	100.0	EXACTX	Intact	Plasmid contig, contig 2	aadA1 (100.00/100.0)
S18	aac(6')-Ib	Amikacin/Kanamycin/Tobramycin	100.00	100.0	BLASTP	Intact	Plasmid contig, contig 2	aac(6')-Ib (100.00/100.0)
S18	blaCTX-M-15	Cephalosporin	100.00	100.0	ALLELEP	Intact	Plasmid contig, contig 2	blaCTX-M-15 (100.00/100.0)
S18	qnrS1	Quinolone	100.00	100.0	ALLELEP	Intact	Plasmid contig, contig 2	qnrS1 (100.00/100.0)
S18	aph(3')-VI	Amikacin/Kanamycin	100.00	100.0	EXACTP	Intact	Plasmid contig, contig 2	aph(3')-VI (100.00/100.0)
S18	blaNDM-5	Carbapenem	100.00	100.0	ALLELEP	Intact	Plasmid contig, contig 2	blaNDM-5 (100.00/100.0)
S18	ble	Bleomycin	100.00	100.0	EXACTP	Intact	Plasmid contig, contig 2	Not reported
S18	mph(A)	Azithromycin/Erythromycin/Spiramycin/Telithromycin	100.00	100.0	BLASTP	Intact	Plasmid contig, contig 2	mph(A) (100.00/99.7)
S18	mrx(A)	Azithromycin/Erythromycin/Spiramycin/Telithromycin	100.00	100.0	EXACTP	Intact	Plasmid contig, contig 2	Not reported
S18	sul1	Sulfonamide	100.00	100.0	EXACTP	Intact	Plasmid contig, contig 2	sul1 (100.00/100.0)
S18	dfrA5	Trimethoprim	100.00	100.0	EXACTX	Intact	Plasmid contig, contig 2	dfrA5 (100.00/100.0)
S18	catB	Chloramphenicol	100.00	100.0	EXACTP	Intact	Plasmid contig, contig 3	Not reported
S18	rmtF1	Aminoglycoside	100.00	100.0	ALLELEP	Intact	Plasmid contig, contig 3	rmtF (100.00/100.0)
S18	aac(6')-Ib	Gentamicin	100.00	100.0	EXACTP	Intact	Plasmid contig, contig 3	aac(6')-Ib-Hangzhou (99.61/100.0)
S18	arr-2	Rifamycin	100.00	100.0	ALLELEP	Intact	Plasmid contig, contig 3	ARR-2 (100.00/100.0)
S18	qnrB1	Quinolone	100.00	100.0	ALLELEP	Intact	Plasmid contig, contig 3	qnrB1 (100.00/100.0)
S18	dfrA50	Trimethoprim	98.73	100.0	BLASTP	Intact	Unclassified contig, contig 4	Not reported
S19	emrD	Efflux	99.49	100.0	BLASTP	Intact	Chromosome, contig 1	Not reported
S19	parC_S80I	Quinolone	99.27	98.8	POINTP	Point variant	Chromosome, contig 1	n/a
S19	oqxB	Nitrofurantoin/Phenicol/Quinolone/Tigecycline	99.81	100.0	BLASTP	Intact	Chromosome, contig 1	OqxB (98.54/100.0)
S19	oqxA	Nitrofurantoin/Phenicol/Quinolone/Tigecycline	100.00	100.0	EXACTP	Intact	Chromosome, contig 1	OqxA (99.49/100.0)
S19	gyrA_S83I	Quinolone	99.77	100.0	POINTP	Point variant	Chromosome, contig 1	n/a
S19	ompK36_D135DGD	Carbapenem	98.92	100.0	POINTP	Point variant	Chromosome, contig 1	n/a
S19	blaSHV-11	Beta-Lactam	100.00	100.0	ALLELEP	Intact	Chromosome, contig 1	blaSHV-11 (99.77/100.0); blaSHV-67 (99.77/100.0)
S19	ompK35_R60LfsTer31	Carbapenem	100.00	100.0	POINTX	Truncated or disrupted	Chromosome, contig 1	n/a
S19	pmrB_T157P	Colistin	99.18	100.0	POINTP	Point variant	Chromosome, contig 1	n/a
S19	fosA	Fosfomycin	99.28	99.3	BLASTP	Intact	Chromosome, contig 1	fosA (98.79/98.3)

Isolate	AMRFinderPlus element	Subclass	Identity (%)	Coverage (%)	Method	Functional status	Location	ResFinder call, identity/coverage (%)
S19	catA1	Chloramphenicol	100.00	66.2	PARTIALP	Truncated or disrupted	Plasmid contig, contig 2	catA1 (99.56/68.8)
S19	ble	Bleomycin	100.00	100.0	EXACTP	Intact	Plasmid contig, contig 2	Not reported
S19	blaNDM-5	Carbapenem	100.00	100.0	ALLELEP	Intact	Plasmid contig, contig 2	blaNDM-5 (100.00/100.0)
S19	aph(3')-VI	Amikacin/Kanamycin	100.00	100.0	EXACTP	Intact	Plasmid contig, contig 2	aph(3')-VI (100.00/100.0)
S19	qnrS1	Quinolone	100.00	100.0	ALLELEP	Intact	Plasmid contig, contig 2	qnrS1 (100.00/100.0)
S19	blaCTX-M-15	Cephalosporin	100.00	100.0	ALLELEP	Intact	Plasmid contig, contig 2	blaCTX-M-15 (100.00/100.0)
S19	aac(6')-Ib	Amikacin/Kanamycin/Tobramycin	100.00	100.0	BLASTP	Intact	Plasmid contig, contig 2	aac(6')-Ib (100.00/100.0)
S19	aadA1	Streptomycin	100.00	100.0	EXACTX	Intact	Plasmid contig, contig 2	aadA1 (100.00/100.0)
S19	blaOXA	Beta-Lactam	99.64	100.0	INTERNAL_STOP	Truncated or disrupted	Plasmid contig, contig 2	blaOXA-9 (99.88/100.0)
S19	blaTEM-1	Beta-Lactam	100.00	100.0	ALLELEP	Intact	Plasmid contig, contig 2	blaTEM-1C (99.88/100.0); blaTEM-1B (99.88/100.0)
S19	aph(3')-Ia	Kanamycin	100.00	100.0	EXACTP	Intact	Plasmid contig, contig 2	aph(3')-Ia (99.88/100.0)
S19	mph(A)	Azithromycin/Erythromycin/Spiramycin/Telithromycin	100.00	100.0	BLASTP	Intact	Plasmid contig, contig 2	mph(A) (100.00/99.7)
S19	mrx(A)	Azithromycin/Erythromycin/Spiramycin/Telithromycin	100.00	100.0	EXACTP	Intact	Plasmid contig, contig 2	Not reported
S19	sul1	Sulfonamide	100.00	100.0	EXACTP	Intact	Plasmid contig, contig 2	sul1 (100.00/100.0)
S19	dfrA5	Trimethoprim	100.00	100.0	EXACTX	Intact	Plasmid contig, contig 2	dfrA5 (100.00/100.0)
S19	catB	Chloramphenicol	100.00	100.0	EXACTP	Intact	Plasmid contig, contig 3	Not reported
S19	rmtF1	Aminoglycoside	100.00	100.0	ALLELEP	Intact	Plasmid contig, contig 3	rmtF (100.00/100.0)
S19	aac(6')-Ib	Gentamicin	100.00	100.0	EXACTP	Intact	Plasmid contig, contig 3	aac(6')-Ib-Hangzhou (99.61/100.0)
S19	arr-2	Rifamycin	100.00	100.0	ALLELEP	Intact	Plasmid contig, contig 3	ARR-2 (100.00/100.0)
S19	qnrB1	Quinolone	100.00	100.0	ALLELEP	Intact	Plasmid contig, contig 3	qnrB1 (100.00/100.0)
S19	dfrA50	Trimethoprim	98.73	100.0	BLASTP	Intact	Unclassified contig, contig 4	Not reported
S19	blaOXA-48	Carbapenem	100.00	100.0	ALLELEP	Intact	Plasmid contig, contig 5	blaOXA-48 (100.00/100.0)
S19	blaCTX-M-14	Cephalosporin	100.00	100.0	ALLELEP	Intact	Plasmid contig, contig 5	blaCTX-M-14b (100.00/100.0)
S19	aph(3")-Ib	Streptomycin	100.00	100.0	EXACTP	Intact	Plasmid contig, contig 5	aph(6)-Id (100.00/100.0); aph(3")-Ib (100.00/99.9)
S19	aph(6)-Id	Streptomycin	100.00	100.0	EXACTP	Intact	Plasmid contig, contig 5	aph(6)-Id (100.00/100.0); aph(3")-Ib (100.00/99.9)
S20	emrD	Efflux	99.49	100.0	BLASTP	Intact	Chromosome, contig 1	Not reported
S20	parC_S80I	Quinolone	99.27	98.8	POINTP	Point variant	Chromosome, contig 1	n/a
S20	oqxB	Nitrofurantoin/Phenicol/Quinolone/Tigecycline	99.81	100.0	BLASTP	Intact	Chromosome, contig 1	OqxB (98.54/100.0)
S20	oqxA	Nitrofurantoin/Phenicol/Quinolone/Tigecycline	100.00	100.0	EXACTP	Intact	Chromosome, contig 1	OqxA (99.49/100.0)

Isolate	AMRFinderPlus element	Subclass	Identity (%)	Coverage (%)	Method	Functional status	Location	ResFinder call, identity/coverage (%)
S20	gyrA_S83I	Quinolone	99.77	100.0	POINTP	Point variant	Chromosome, contig 1	n/a
S20	ompK36_D135DGD	Carbapenem	98.92	100.0	POINTP	Point variant	Chromosome, contig 1	n/a
S20	blaSHV-11	Beta-Lactam	100.00	100.0	ALLELEP	Intact	Chromosome, contig 1	blaSHV-11 (99.77/100.0); blaSHV-67 (99.77/100.0)
S20	ompK35_R60LfsTer31	Carbapenem	100.00	100.0	POINTX	Truncated or disrupted	Chromosome, contig 1	n/a
S20	pmrB_T157P	Colistin	99.18	100.0	POINTP	Point variant	Chromosome, contig 1	n/a
S20	fosA	Fosfomycin	99.28	99.3	BLASTP	Intact	Chromosome, contig 1	fosA (98.79/98.3)
S20	catA1	Chloramphenicol	100.00	66.2	PARTIALP	Truncated or disrupted	Plasmid contig, contig 2	catA1 (99.56/68.8)
S20	ble	Bleomycin	100.00	100.0	EXACTP	Intact	Plasmid contig, contig 2	Not reported
S20	blaNDM-5	Carbapenem	100.00	100.0	ALLELEP	Intact	Plasmid contig, contig 2	blaNDM-5 (100.00/100.0)
S20	aph(3')-VI	Amikacin/Kanamycin	100.00	100.0	EXACTP	Intact	Plasmid contig, contig 2	aph(3')-VI (100.00/100.0)
S20	qnrS1	Quinolone	100.00	100.0	ALLELEP	Intact	Plasmid contig, contig 2	qnrS1 (100.00/100.0)
S20	blaCTX-M-15	Cephalosporin	100.00	100.0	ALLELEP	Intact	Plasmid contig, contig 2	blaCTX-M-15 (100.00/100.0)
S20	aac(6')-Ib	Amikacin/Kanamycin/Tobramycin	100.00	100.0	BLASTP	Intact	Plasmid contig, contig 2	aac(6')-Ib (100.00/100.0)
S20	aadA1	Streptomycin	100.00	100.0	EXACTX	Intact	Plasmid contig, contig 2	aadA1 (100.00/100.0)
S20	blaOXA	Beta-Lactam	99.64	100.0	INTERNAL_STOP	Truncated or disrupted	Plasmid contig, contig 2	blaOXA-9 (99.88/100.0)
S20	blaTEM-1	Beta-Lactam	100.00	100.0	ALLELEP	Intact	Plasmid contig, contig 2	blaTEM-1C (99.88/100.0); blaTEM-1B (99.88/100.0)
S20	aph(3')-Ia	Kanamycin	100.00	100.0	EXACTP	Intact	Plasmid contig, contig 2	aph(3')-Ia (99.88/100.0)
S20	mph(A)	Azithromycin/Erythromycin/Spiramycin/Telithromycin	100.00	100.0	BLASTP	Intact	Plasmid contig, contig 2	mph(A) (100.00/99.7)
S20	mrx(A)	Azithromycin/Erythromycin/Spiramycin/Telithromycin	100.00	100.0	EXACTP	Intact	Plasmid contig, contig 2	Not reported
S20	sul1	Sulfonamide	100.00	100.0	EXACTP	Intact	Plasmid contig, contig 2	sul1 (100.00/100.0)
S20	dfrA5	Trimethoprim	100.00	100.0	EXACTX	Intact	Plasmid contig, contig 2	dfrA5 (100.00/100.0)
S20	catB	Chloramphenicol	100.00	100.0	EXACTP	Intact	Plasmid contig, contig 3	Not reported
S20	rmtF1	Aminoglycoside	100.00	100.0	ALLELEP	Intact	Plasmid contig, contig 3	rmtF (100.00/100.0)
S20	aac(6')-Ib	Gentamicin	100.00	100.0	EXACTP	Intact	Plasmid contig, contig 3	aac(6')-Ib-Hangzhou (99.61/100.0)
S20	arr-2	Rifamycin	100.00	100.0	ALLELEP	Intact	Plasmid contig, contig 3	ARR-2 (100.00/100.0)
S20	qnrB1	Quinolone	100.00	100.0	ALLELEP	Intact	Plasmid contig, contig 3	qnrB1 (100.00/100.0)
S20	dfrA50	Trimethoprim	98.73	100.0	BLASTP	Intact	Unclassified contig, contig 4	Not reported
S20	blaOXA-48	Carbapenem	100.00	100.0	ALLELEP	Intact	Plasmid contig, contig 5	blaOXA-48 (100.00/100.0)
S20	blaCTX-M-14	Cephalosporin	100.00	100.0	ALLELEP	Intact	Plasmid contig, contig 5	blaCTX-M-14b (100.00/100.0)
S20	aph(3")-Ib	Streptomycin	100.00	100.0	EXACTP	Intact	Plasmid contig, contig 5	aph(6)-Id (100.00/100.0); aph(3")-Ib (100.00/99.9)
S20	aph(6)-Id	Streptomycin	100.00	100.0	EXACTP	Intact	Plasmid contig, contig 5	aph(6)-Id (100.00/100.0); aph(3")-Ib (100.00/99.9)
S21	emrD	Efflux	99.49	100.0	BLASTP	Intact	Chromosome, contig 1	Not reported

Isolate	AMRFinderPlus element	Subclass	Identity (%)	Coverage (%)	Method	Functional status	Location	ResFinder call, identity/coverage (%)
S21	oqxB19	Phenicol/Quinolone	100.00	100.0	ALLELEP	Intact	Chromosome, contig 1	OqxB (98.45/100.0)
S21	oqxA10	Phenicol/Quinolone	100.00	100.0	ALLELEP	Intact	Chromosome, contig 1	OqxA (99.15/100.0)
S21	blaSHV-11	Beta-Lactam	100.00	100.0	ALLELEP	Intact	Chromosome, contig 1	blaSHV-89 (99.88/100.0); blaSHV-85 (99.88/100.0); blaSHV-79 (99.88/100.0); blaSHV-56 (99.88/100.0); blaSHV-40 (99.88/100.0)
S21	fosA	Fosfomycin	99.28	99.3	BLASTP	Intact	Chromosome, contig 1	fosA6 (99.76/98.3)
S21	tet(A)	Tetracycline	100.00	100.0	EXACTP	Intact	Plasmid contig, contig 2	tet(A) (100.00/100.0)
S21	qnrB1	Quinolone	100.00	100.0	ALLELEP	Intact	Plasmid contig, contig 2	qnrB1 (100.00/100.0)
S21	dfrA14	Trimethoprim	100.00	100.0	EXACTX	Intact	Plasmid contig, contig 2	dfrA14 (100.00/100.0)
S21	blaCTX-M-15	Cephalosporin	100.00	100.0	ALLELEP	Intact	Plasmid contig, contig 2	blaCTX-M-15 (100.00/100.0)
S21	blaTEM-1	Beta-Lactam	100.00	100.0	ALLELEP	Intact	Plasmid contig, contig 2	blaTEM-1B (100.00/100.0)
S21	aph(6)-Id	Streptomycin	100.00	100.0	EXACTX	Intact	Plasmid contig, contig 2	aph(6)-Id (100.00/100.0); aph(3")-Ib (98.96/35.8)
S21	aph(3")-Ib	Streptomycin	100.00	58.0	PARTIALP	Truncated or disrupted	Plasmid contig, contig 2	aph(3")-Ib (100.00/57.8)
S21	sul2	Sulfonamide	100.00	100.0	EXACTP	Intact	Plasmid contig, contig 2	sul2 (100.00/100.0)
S21	ble	Bleomycin	100.00	100.0	EXACTP	Intact	Plasmid contig, contig 3	Not reported
S21	blaNDM-7	Carbapenem	100.00	100.0	ALLELEP	Intact	Plasmid contig, contig 3	blaNDM-7 (100.00/100.0)
S22	emrD	Efflux	99.49	100.0	BLASTP	Intact	Chromosome, contig 1	Not reported
S22	parC_S80I	Quinolone	99.27	98.8	POINTP	Point variant	Chromosome, contig 1	n/a
S22	oqxB	Nitrofurantoin/Phenicol/Quinolone/Tigecycline	99.81	100.0	BLASTP	Intact	Chromosome, contig 1	OqxB (98.54/100.0)
S22	oqxA	Nitrofurantoin/Phenicol/Quinolone/Tigecycline	100.00	100.0	EXACTP	Intact	Chromosome, contig 1	OqxA (99.49/100.0)
S22	gyrA_S83I	Quinolone	99.77	100.0	POINTP	Point variant	Chromosome, contig 1	n/a
S22	ompK36_D135DGD	Carbapenem	98.64	100.0	POINTX	Point variant	Chromosome, contig 1	n/a
S22	ompK36_Q313Ter	Carbapenem	98.64	100.0	POINTX	Point variant	Chromosome, contig 1	n/a
S22	blaSHV-11	Beta-Lactam	100.00	100.0	ALLELEP	Intact	Chromosome, contig 1	blaSHV-11 (99.77/100.0); blaSHV-67 (99.77/100.0)
S22	ompK35_R60LfsTer31	Carbapenem	100.00	100.0	POINTX	Truncated or disrupted	Chromosome, contig 1	n/a
S22	fosA	Fosfomycin	99.28	99.3	BLASTP	Intact	Chromosome, contig 1	fosA (98.79/98.3)
S22	catA1	Chloramphenicol	100.00	66.2	PARTIALP	Truncated or disrupted	Plasmid contig, contig 2	catA1 (99.56/68.8)
S22	blaTEM-1	Beta-Lactam	100.00	100.0	ALLELEP	Intact	Plasmid contig, contig 2	blaTEM-1C (99.88/100.0); blaTEM-1B (99.88/100.0)
S22	blaOXA	Beta-Lactam	99.64	100.0	INTERNAL_STOP	Truncated or disrupted	Plasmid contig, contig 2	blaOXA-9 (99.88/100.0)
S22	aadA1	Streptomycin	100.00	100.0	EXACTX	Intact	Plasmid contig, contig 2	aadA1 (100.00/100.0)
S22	aac(6')-Ib	Amikacin/Kanamycin/Tobramycin	100.00	100.0	BLASTP	Intact	Plasmid contig, contig 2	aac(6')-Ib (100.00/100.0)
S22	blaCTX-M-15	Cephalosporin	100.00	100.0	ALLELEP	Intact	Plasmid contig, contig 2	blaCTX-M-15 (100.00/100.0)

Isolate	AMRFinderPlus element	Subclass	Identity (%)	Coverage (%)	Method	Functional status	Location	ResFinder call, identity/coverage (%)
S22	qnrS1	Quinolone	100.00	100.0	ALLELEP	Intact	Plasmid contig, contig 2	qnrS1 (100.00/100.0)
S22	aph(3')-VI	Amikacin/Kanamycin	100.00	100.0	EXACTP	Intact	Plasmid contig, contig 2	aph(3')-VI (100.00/100.0)
S22	blaNDM-5	Carbapenem	100.00	100.0	ALLELEP	Intact	Plasmid contig, contig 2	blaNDM-5 (100.00/100.0)
S22	ble	Bleomycin	100.00	100.0	EXACTP	Intact	Plasmid contig, contig 2	Not reported
S22	mph(A)	Azithromycin/Erythromycin/Spiramycin/Telithromycin	100.00	100.0	BLASTP	Intact	Plasmid contig, contig 2	mph(A) (100.00/99.7)
S22	mrx(A)	Azithromycin/Erythromycin/Spiramycin/Telithromycin	100.00	100.0	EXACTP	Intact	Plasmid contig, contig 2	Not reported
S22	sul1	Sulfonamide	100.00	100.0	EXACTP	Intact	Plasmid contig, contig 2	sul1 (100.00/100.0)
S22	dfrA5	Trimethoprim	100.00	100.0	EXACTX	Intact	Plasmid contig, contig 2	dfrA5 (100.00/100.0)
S22	arr-2	Rifamycin	100.00	100.0	ALLELEP	Intact	Plasmid contig, contig 3	ARR-2 (100.00/100.0)
S22	aac(6')-Ib	Gentamicin	100.00	100.0	EXACTP	Intact	Plasmid contig, contig 3	aac(6')-Ib-Hangzhou (99.61/100.0)
S22	rmtF1	Aminoglycoside	100.00	100.0	ALLELEP	Intact	Plasmid contig, contig 3	rmtF (100.00/100.0)
S22	catB	Chloramphenicol	100.00	100.0	EXACTP	Intact	Plasmid contig, contig 3	Not reported
S22	qnrB1	Quinolone	100.00	100.0	ALLELEP	Intact	Plasmid contig, contig 3	qnrB1 (100.00/100.0)
S22	dfrA50	Trimethoprim	98.73	100.0	BLASTP	Intact	Unclassified contig, contig 4	Not reported
S22	blaOXA-48	Carbapenem	100.00	100.0	ALLELEP	Intact	Plasmid contig, contig 5	blaOXA-48 (100.00/100.0)
S22	blaCTX-M-14	Cephalosporin	100.00	100.0	ALLELEP	Intact	Plasmid contig, contig 5	blaCTX-M-14b (100.00/100.0)
S22	aph(3")-Ib	Streptomycin	100.00	100.0	EXACTP	Intact	Plasmid contig, contig 5	aph(6)-Id (100.00/100.0); aph(3")-Ib (100.00/99.9)
S22	aph(6)-Id	Streptomycin	100.00	100.0	EXACTP	Intact	Plasmid contig, contig 5	aph(6)-Id (100.00/100.0); aph(3")-Ib (100.00/99.9)
S23	emrD	Efflux	99.49	100.0	BLASTP	Intact	Chromosome, contig 1	Not reported
S23	parC_S80I	Quinolone	99.27	98.8	POINTP	Point variant	Chromosome, contig 1	n/a
S23	oqxB	Nitrofurantoin/Phenicol/Quinolone/Tigecycline	99.81	100.0	BLASTP	Intact	Chromosome, contig 1	OqxB (98.54/100.0)
S23	oqxA	Nitrofurantoin/Phenicol/Quinolone/Tigecycline	100.00	100.0	EXACTP	Intact	Chromosome, contig 1	OqxA (99.49/100.0)
S23	gyrA_S83I	Quinolone	99.77	100.0	POINTP	Point variant	Chromosome, contig 1	n/a
S23	ompK36_D135DGD	Carbapenem	98.92	100.0	POINTP	Point variant	Chromosome, contig 1	n/a
S23	blaSHV-11	Beta-Lactam	100.00	100.0	ALLELEP	Intact	Chromosome, contig 1	blaSHV-11 (99.77/100.0); blaSHV-67 (99.77/100.0)
S23	ompK35_R60LfsTer31	Carbapenem	100.00	100.0	POINTX	Truncated or disrupted	Chromosome, contig 1	n/a
S23	fosA	Fosfomycin	99.28	99.3	BLASTP	Intact	Chromosome, contig 1	fosA (98.79/98.3)
S23	catA1	Chloramphenicol	100.00	66.2	PARTIALP	Truncated or disrupted	Plasmid contig, contig 2	catA1 (99.56/68.8)
S23	ble	Bleomycin	100.00	100.0	EXACTP	Intact	Plasmid contig, contig 2	Not reported
S23	blaNDM-5	Carbapenem	100.00	100.0	ALLELEP	Intact	Plasmid contig, contig 2	blaNDM-5 (100.00/100.0)

Isolate	AMRFinderPlus element	Subclass	Identity (%)	Coverage (%)	Method	Functional status	Location	ResFinder call, identity/coverage (%)
S23	aph(3')-VI	Amikacin/Kanamycin	100.00	100.0	EXACTP	Intact	Plasmid contig, contig 2	aph(3')-VI (100.00/100.0)
S23	qnrS1	Quinolone	100.00	100.0	ALLELEP	Intact	Plasmid contig, contig 2	qnrS1 (100.00/100.0)
S23	blaCTX-M-15	Cephalosporin	100.00	100.0	ALLELEP	Intact	Plasmid contig, contig 2	blaCTX-M-15 (100.00/100.0)
S23	aac(6')-Ib	Amikacin/Kanamycin/Tobramycin	100.00	100.0	BLASTP	Intact	Plasmid contig, contig 2	aac(6')-Ib (100.00/100.0)
S23	aadA1	Streptomycin	100.00	100.0	EXACTX	Intact	Plasmid contig, contig 2	aadA1 (100.00/100.0)
S23	blaOXA	Beta-Lactam	99.64	100.0	INTERNAL_STOP	Truncated or disrupted	Plasmid contig, contig 2	blaOXA-9 (99.88/100.0)
S23	blaTEM-1	Beta-Lactam	100.00	100.0	ALLELEP	Intact	Plasmid contig, contig 2	blaTEM-1C (99.88/100.0); blaTEM-1B (99.88/100.0)
S23	arr-2	Rifamycin	100.00	100.0	ALLELEP	Intact	Plasmid contig, contig 2	ARR-2 (100.00/100.0)
S23	aac(6')-Ib	Gentamicin	100.00	100.0	EXACTP	Intact	Plasmid contig, contig 2	aac(6')-Ib-Hangzhou (99.61/100.0)
S23	rmtF1	Aminoglycoside	100.00	100.0	ALLELEP	Intact	Plasmid contig, contig 2	rmtF (100.00/100.0)
S23	catB	Chloramphenicol	100.00	100.0	EXACTP	Intact	Plasmid contig, contig 2	Not reported
S23	dfrA5	Trimethoprim	100.00	100.0	EXACTX	Intact	Plasmid contig, contig 2	dfrA5 (100.00/100.0)
S23	sul1	Sulfonamide	100.00	100.0	EXACTP	Intact	Plasmid contig, contig 2	sul1 (100.00/100.0)
S23	mrx(A)	Azithromycin/Erythromycin/Spiramycin/Telithromycin	100.00	100.0	EXACTP	Intact	Plasmid contig, contig 2	Not reported
S23	mph(A)	Azithromycin/Erythromycin/Spiramycin/Telithromycin	100.00	100.0	BLASTP	Intact	Plasmid contig, contig 2	mph(A) (100.00/99.7)
S23	aph(3')-Ia	Kanamycin	100.00	100.0	EXACTP	Intact	Plasmid contig, contig 2	aph(3')-Ia (99.88/100.0)
S23	dfrA50	Trimethoprim	98.73	100.0	BLASTP	Intact	Unclassified contig, contig 3	Not reported
S23	blaKPC-3	Carbapenem	100.00	100.0	ALLELEP	Intact	Plasmid contig, contig 4	blaKPC-3 (100.00/100.0)
S23	aac(6')-Ib-cr5	Amikacin/Kanamycin/Quinolone/Tobramycin	100.00	100.0	ALLELEX	Intact	Plasmid contig, contig 4	aac(6')-Ib-cr (100.00/100.0)
S23	blaOXA-1	Cephalosporin	100.00	100.0	ALLELEP	Intact	Plasmid contig, contig 4	blaOXA-1 (100.00/100.0)
S23	catB3	Chloramphenicol	100.00	70.0	PARTIALP	Truncated or disrupted	Plasmid contig, contig 4	catB3 (100.00/69.8)
S23	tet(A)	Tetracycline	100.00	100.0	EXACTP	Intact	Plasmid contig, contig 4	tet(A) (100.00/100.0)
S23	qnrB1	Quinolone	100.00	100.0	ALLELEP	Intact	Plasmid contig, contig 4	qnrB1 (100.00/100.0)
S23	dfrA14	Trimethoprim	100.00	100.0	EXACTX	Intact	Plasmid contig, contig 4	dfrA14 (100.00/100.0)
S23	blaOXA-48	Carbapenem	100.00	100.0	ALLELEP	Intact	Plasmid contig, contig 5	blaOXA-48 (100.00/100.0)
S23	blaCTX-M-14	Cephalosporin	100.00	100.0	ALLELEP	Intact	Plasmid contig, contig 5	blaCTX-M-14b (100.00/100.0)
S23	aph(3")-Ib	Streptomycin	100.00	100.0	EXACTP	Intact	Plasmid contig, contig 5	aph(6)-Id (100.00/100.0); aph(3")-Ib (100.00/99.9)
S23	aph(6)-Id	Streptomycin	100.00	100.0	EXACTP	Intact	Plasmid contig, contig 5	aph(6)-Id (100.00/100.0); aph(3")-Ib (100.00/99.9)
S24	emrD	Efflux	99.49	100.0	BLASTP	Intact	Chromosome, contig 1	Not reported
S24	parC_S80I	Quinolone	99.27	98.8	POINTP	Point variant	Chromosome, contig 1	n/a

Isolate	AMRFinderPlus element	Subclass	Identity (%)	Coverage (%)	Method	Functional status	Location	ResFinder call, identity/coverage (%)
S24	oqxB	Nitrofurantoin/Phenicol/Quinolone/Tigecycline	99.81	100.0	BLASTP	Intact	Chromosome, contig 1	OqxB (98.54/100.0)
S24	oqxA	Nitrofurantoin/Phenicol/Quinolone/Tigecycline	100.00	100.0	EXACTP	Intact	Chromosome, contig 1	OqxA (99.49/100.0)
S24	gyrA_S83I	Quinolone	99.77	100.0	POINTP	Point variant	Chromosome, contig 1	n/a
S24	ompK36_D135DGD	Carbapenem	98.92	100.0	POINTP	Point variant	Chromosome, contig 1	n/a
S24	blaSHV-11	Beta-Lactam	100.00	100.0	ALLELEP	Intact	Chromosome, contig 1	blaSHV-11 (99.77/100.0); blaSHV-67 (99.77/100.0)
S24	ompK35_R60LfsTer31	Carbapenem	100.00	100.0	POINTX	Truncated or disrupted	Chromosome, contig 1	n/a
S24	fosA	Fosfomycin	99.28	99.3	BLASTP	Intact	Chromosome, contig 1	fosA (98.79/98.3)
S24	catA1	Chloramphenicol	100.00	66.2	PARTIALP	Truncated or disrupted	Plasmid contig, contig 2	catA1 (99.56/68.8)
S24	ble	Bleomycin	100.00	100.0	EXACTP	Intact	Plasmid contig, contig 2	Not reported
S24	blaNDM-5	Carbapenem	100.00	100.0	ALLELEP	Intact	Plasmid contig, contig 2	blaNDM-5 (100.00/100.0)
S24	aph(3')-VI	Amikacin/Kanamycin	100.00	100.0	EXACTP	Intact	Plasmid contig, contig 2	aph(3')-VI (100.00/100.0)
S24	qnrS1	Quinolone	100.00	100.0	ALLELEP	Intact	Plasmid contig, contig 2	qnrS1 (100.00/100.0)
S24	blaCTX-M-15	Cephalosporin	100.00	100.0	ALLELEP	Intact	Plasmid contig, contig 2	blaCTX-M-15 (100.00/100.0)
S24	aac(6')-Ib	Amikacin/Kanamycin/Tobramycin	100.00	100.0	BLASTP	Intact	Plasmid contig, contig 2	aac(6')-Ib (100.00/100.0)
S24	aadA1	Streptomycin	100.00	100.0	EXACTX	Intact	Plasmid contig, contig 2	aadA1 (100.00/100.0)
S24	blaOXA	Beta-Lactam	99.64	100.0	INTERNAL_STOP	Truncated or disrupted	Plasmid contig, contig 2	blaOXA-9 (99.88/100.0)
S24	blaTEM-1	Beta-Lactam	100.00	100.0	ALLELEP	Intact	Plasmid contig, contig 2	blaTEM-1C (99.88/100.0); blaTEM-1B (99.88/100.0)
S24	arr-2	Rifamycin	100.00	100.0	ALLELEP	Intact	Plasmid contig, contig 2	ARR-2 (100.00/100.0)
S24	aac(6')-Ib	Gentamicin	100.00	100.0	EXACTP	Intact	Plasmid contig, contig 2	aac(6')-Ib-Hangzhou (99.61/100.0)
S24	rmtF1	Aminoglycoside	100.00	100.0	ALLELEP	Intact	Plasmid contig, contig 2	rmtF (100.00/100.0)
S24	catB	Chloramphenicol	100.00	100.0	EXACTP	Intact	Plasmid contig, contig 2	Not reported
S24	dfrA5	Trimethoprim	100.00	100.0	EXACTX	Intact	Plasmid contig, contig 2	dfrA5 (100.00/100.0)
S24	sul1	Sulfonamide	100.00	100.0	EXACTP	Intact	Plasmid contig, contig 2	sul1 (100.00/100.0)
S24	mrx(A)	Azithromycin/Erythromycin/Spiramycin/Telithromycin	100.00	100.0	EXACTP	Intact	Plasmid contig, contig 2	Not reported
S24	mph(A)	Azithromycin/Erythromycin/Spiramycin/Telithromycin	100.00	100.0	BLASTP	Intact	Plasmid contig, contig 2	mph(A) (100.00/99.7)
S24	aph(3')-Ia	Kanamycin	100.00	100.0	EXACTP	Intact	Plasmid contig, contig 2	aph(3')-Ia (99.88/100.0)
S24	dfrA50	Trimethoprim	98.73	100.0	BLASTP	Intact	Unclassified contig, contig 3	Not reported
S24	blaOXA-48	Carbapenem	100.00	100.0	ALLELEP	Intact	Plasmid contig, contig 4	blaOXA-48 (100.00/100.0)

Isolate	AMRFinderPlus element	Subclass	Identity (%)	Coverage (%)	Method	Functional status	Location	ResFinder call, identity/coverage (%)
S24	blaCTX-M-14	Cephalosporin	100.00	100.0	ALLELEP	Intact	Plasmid contig, contig 4	blaCTX-M-14b (100.00/100.0)
S24	aph(3")-Ib	Streptomycin	100.00	100.0	EXACTP	Intact	Plasmid contig, contig 4	aph(6)-Id (100.00/100.0); aph(3")-Ib (100.00/99.9)
S24	aph(6)-Id	Streptomycin	100.00	100.0	EXACTP	Intact	Plasmid contig, contig 4	aph(6)-Id (100.00/100.0); aph(3")-Ib (100.00/99.9)
S25	emrD	Efflux	99.49	100.0	BLASTP	Intact	Chromosome, contig 1	Not reported
S25	parC_S80I	Quinolone	99.27	98.8	POINTP	Point variant	Chromosome, contig 1	n/a
S25	oqxB	Nitrofurantoin/Phenicol/Quinolone/Tigecycline	99.81	100.0	BLASTP	Intact	Chromosome, contig 1	OqxB (98.54/100.0)
S25	oqxA	Nitrofurantoin/Phenicol/Quinolone/Tigecycline	100.00	100.0	EXACTP	Intact	Chromosome, contig 1	OqxA (99.49/100.0)
S25	gyrA_S83I	Quinolone	99.77	100.0	POINTP	Point variant	Chromosome, contig 1	n/a
S25	ompK36_D135DGD	Carbapenem	98.92	100.0	POINTP	Point variant	Chromosome, contig 1	n/a
S25	blaSHV-11	Beta-Lactam	100.00	100.0	ALLELEP	Intact	Chromosome, contig 1	blaSHV-11 (99.77/100.0); blaSHV-67 (99.77/100.0)
S25	ompK35_R60LfsTer31	Carbapenem	100.00	100.0	POINTX	Truncated or disrupted	Chromosome, contig 1	n/a
S25	ramR_K104RfsTer6	Chloramphenicol/Fluoroquinolone/Temocillin/Tetracycline/Tigecycline	99.48	100.0	POINTX	Truncated or disrupted	Chromosome, contig 1	n/a
S25	fosA	Fosfomycin	99.28	99.3	BLASTP	Intact	Chromosome, contig 1	fosA (98.79/98.3)
S25	catA1	Chloramphenicol	100.00	66.2	PARTIALP	Truncated or disrupted	Plasmid contig, contig 2	catA1 (99.56/68.8)
S25	ble	Bleomycin	100.00	100.0	EXACTP	Intact	Plasmid contig, contig 2	Not reported
S25	blaNDM-5	Carbapenem	100.00	100.0	ALLELEP	Intact	Plasmid contig, contig 2	blaNDM-5 (100.00/100.0)
S25	aph(3')-VI	Amikacin/Kanamycin	100.00	100.0	EXACTP	Intact	Plasmid contig, contig 2	aph(3')-VI (100.00/100.0)
S25	qnrS1	Quinolone	100.00	100.0	ALLELEP	Intact	Plasmid contig, contig 2	qnrS1 (100.00/100.0)
S25	blaCTX-M-15	Cephalosporin	100.00	100.0	ALLELEP	Intact	Plasmid contig, contig 2	blaCTX-M-15 (100.00/100.0)
S25	aac(6')-Ib	Amikacin/Kanamycin/Tobramycin	100.00	100.0	BLASTP	Intact	Plasmid contig, contig 2	aac(6')-Ib (100.00/100.0)
S25	aadA1	Streptomycin	100.00	100.0	EXACTX	Intact	Plasmid contig, contig 2	aadA1 (100.00/100.0)
S25	blaOXA	Beta-Lactam	99.64	100.0	INTERNAL_STOP	Truncated or disrupted	Plasmid contig, contig 2	blaOXA-9 (99.88/100.0)
S25	blaTEM-1	Beta-Lactam	100.00	100.0	ALLELEP	Intact	Plasmid contig, contig 2	blaTEM-1C (99.88/100.0); blaTEM-1B (99.88/100.0)
S25	arr-2	Rifamycin	100.00	100.0	ALLELEP	Intact	Plasmid contig, contig 2	ARR-2 (100.00/100.0)
S25	aac(6')-Ib	Gentamicin	100.00	100.0	EXACTP	Intact	Plasmid contig, contig 2	aac(6')-Ib-Hangzhou (99.61/100.0)
S25	rmtF1	Aminoglycoside	100.00	100.0	ALLELEP	Intact	Plasmid contig, contig 2	rmtF (100.00/100.0)
S25	catB	Chloramphenicol	100.00	100.0	EXACTP	Intact	Plasmid contig, contig 2	Not reported
S25	dfrA5	Trimethoprim	100.00	100.0	EXACTX	Intact	Plasmid contig, contig 2	dfrA5 (100.00/100.0)
S25	sul1	Sulfonamide	100.00	100.0	EXACTP	Intact	Plasmid contig, contig 2	sul1 (100.00/100.0)

Isolate	AMRFinderPlus element	Subclass	Identity (%)	Coverage (%)	Method	Functional status	Location	ResFinder call, identity/coverage (%)
S25	mrx(A)	Azithromycin/Erythromycin/Spiramycin/Telithromycin	100.00	100.0	EXACTP	Intact	Plasmid contig, contig 2	Not reported
S25	mph(A)	Azithromycin/Erythromycin/Spiramycin/Telithromycin	100.00	100.0	BLASTP	Intact	Plasmid contig, contig 2	mph(A) (100.00/99.7)
S25	aph(3')-Ia	Kanamycin	100.00	100.0	EXACTP	Intact	Plasmid contig, contig 2	aph(3')-Ia (99.88/100.0)
S25	catB	Chloramphenicol	100.00	100.0	EXACTP	Intact	Plasmid contig, contig 3	Not reported
S25	rmtF1	Aminoglycoside	100.00	100.0	ALLELEP	Intact	Plasmid contig, contig 3	rmtF (100.00/100.0)
S25	aac(6')-Ib	Gentamicin	100.00	100.0	EXACTP	Intact	Plasmid contig, contig 3	aac(6')-Ib-Hangzhou (99.61/100.0)
S25	arr-2	Rifamycin	100.00	100.0	ALLELEP	Intact	Plasmid contig, contig 3	ARR-2 (100.00/100.0)
S25	dfrA50	Trimethoprim	98.73	100.0	BLASTP	Intact	Plasmid contig, contig 3	Not reported
S25	blaOXA-48	Carbapenem	100.00	100.0	ALLELEP	Intact	Plasmid contig, contig 4	blaOXA-48 (100.00/100.0)
S25	blaCTX-M-14	Cephalosporin	100.00	100.0	ALLELEP	Intact	Plasmid contig, contig 4	blaCTX-M-14b (100.00/100.0)
S25	aph(3")-Ib	Streptomycin	100.00	100.0	EXACTP	Intact	Plasmid contig, contig 4	aph(6)-Id (100.00/100.0); aph(3")-Ib (100.00/99.9)
S25	aph(6)-Id	Streptomycin	100.00	100.0	EXACTP	Intact	Plasmid contig, contig 4	aph(6)-Id (100.00/100.0); aph(3")-Ib (100.00/99.9)
S26	emrD	Efflux	99.49	100.0	BLASTP	Intact	Chromosome, contig 1	Not reported
S26	parC_S80I	Quinolone	99.27	98.8	POINTP	Point variant	Chromosome, contig 1	n/a
S26	oqxB	Nitrofurantoin/Phenicol/Quinolone/Tigecycline	99.81	100.0	BLASTP	Intact	Chromosome, contig 1	OqxB (98.54/100.0)
S26	oqxA	Nitrofurantoin/Phenicol/Quinolone/Tigecycline	100.00	100.0	EXACTP	Intact	Chromosome, contig 1	OqxA (99.49/100.0)
S26	gyrA_S83I	Quinolone	99.77	100.0	POINTP	Point variant	Chromosome, contig 1	n/a
S26	ompK36_D135DGD	Carbapenem	98.92	100.0	POINTP	Point variant	Chromosome, contig 1	n/a
S26	blaSHV-11	Beta-Lactam	100.00	100.0	ALLELEP	Intact	Chromosome, contig 1	blaSHV-11 (99.77/100.0); blaSHV-67 (99.77/100.0)
S26	ompK35_R60LfsTer31	Carbapenem	100.00	100.0	POINTX	Truncated or disrupted	Chromosome, contig 1	n/a
S26	fosA	Fosfomycin	99.28	99.3	BLASTP	Intact	Chromosome, contig 1	fosA (98.79/98.3)
S26	catB	Chloramphenicol	100.00	100.0	EXACTP	Intact	Plasmid contig, contig 2	Not reported
S26	rmtF1	Aminoglycoside	100.00	100.0	ALLELEP	Intact	Plasmid contig, contig 2	rmtF (100.00/100.0)
S26	aac(6')-Ib	Gentamicin	100.00	100.0	EXACTP	Intact	Plasmid contig, contig 2	aac(6')-Ib-Hangzhou (99.61/100.0)
S26	arr-2	Rifamycin	100.00	100.0	ALLELEP	Intact	Plasmid contig, contig 2	ARR-2 (100.00/100.0)
S26	catA1	Chloramphenicol	100.00	66.2	PARTIALP	Truncated or disrupted	Plasmid contig, contig 2	catA1 (99.56/68.8)
S26	ble	Bleomycin	100.00	100.0	EXACTP	Intact	Plasmid contig, contig 2	Not reported
S26	blaNDM-5	Carbapenem	100.00	100.0	ALLELEP	Intact	Plasmid contig, contig 2	blaNDM-5 (100.00/100.0)
S26	aph(3')-VI	Amikacin/Kanamycin	100.00	100.0	EXACTP	Intact	Plasmid contig, contig 2	aph(3')-VI (100.00/100.0)
S26	qnrS1	Quinolone	100.00	100.0	ALLELEP	Intact	Plasmid contig, contig 2	qnrS1 (100.00/100.0)
S26	blaCTX-M-15	Cephalosporin	100.00	100.0	ALLELEP	Intact	Plasmid contig, contig 2	blaCTX-M-15 (100.00/100.0)

Isolate	AMRFinderPlus element	Subclass	Identity (%)	Coverage (%)	Method	Functional status	Location	ResFinder call, identity/coverage (%)
S26	aac(6')-Ib	Amikacin/Kanamycin/Tobramycin	100.00	100.0	BLASTP	Intact	Plasmid contig, contig 2	aac(6')-Ib (100.00/100.0)
S26	aadA1	Streptomycin	100.00	100.0	EXACTX	Intact	Plasmid contig, contig 2	aadA1 (100.00/100.0)
S26	blaOXA	Beta-Lactam	99.64	100.0	INTERNAL_STOP	Truncated or disrupted	Plasmid contig, contig 2	blaOXA-9 (99.88/100.0)
S26	blaTEM-1	Beta-Lactam	100.00	100.0	ALLELEP	Intact	Plasmid contig, contig 2	blaTEM-1C (99.88/100.0); blaTEM-1B (99.88/100.0)
S26	arr-2	Rifamycin	100.00	100.0	ALLELEP	Intact	Plasmid contig, contig 2	ARR-2 (100.00/100.0)
S26	aac(6')-Ib	Gentamicin	100.00	100.0	EXACTP	Intact	Plasmid contig, contig 2	aac(6')-Ib-Hangzhou (99.61/100.0)
S26	rmtF1	Aminoglycoside	100.00	100.0	ALLELEP	Intact	Plasmid contig, contig 2	rmtF (100.00/100.0)
S26	catB	Chloramphenicol	100.00	100.0	EXACTP	Intact	Plasmid contig, contig 2	Not reported
S26	aph(3')-Ia	Kanamycin	100.00	100.0	EXACTP	Intact	Plasmid contig, contig 2	aph(3')-Ia (99.88/100.0)
S26	mph(A)	Azithromycin/Erythromycin/Spiramycin/Telithromycin	100.00	100.0	BLASTP	Intact	Plasmid contig, contig 2	mph(A) (100.00/99.7)
S26	mrx(A)	Azithromycin/Erythromycin/Spiramycin/Telithromycin	100.00	100.0	EXACTP	Intact	Plasmid contig, contig 2	Not reported
S26	sul1	Sulfonamide	100.00	100.0	EXACTP	Intact	Plasmid contig, contig 2	sul1 (100.00/100.0)
S26	dfrA5	Trimethoprim	100.00	100.0	EXACTX	Intact	Plasmid contig, contig 2	dfrA5 (100.00/100.0)
S26	dfrA50	Trimethoprim	98.73	100.0	BLASTP	Intact	Plasmid contig, contig 2	Not reported
S26	blaOXA-48	Carbapenem	100.00	100.0	ALLELEP	Intact	Plasmid contig, contig 3	blaOXA-48 (100.00/100.0)
S26	blaCTX-M-14	Cephalosporin	100.00	100.0	ALLELEP	Intact	Plasmid contig, contig 3	blaCTX-M-14b (100.00/100.0)
S26	aph(3")-Ib	Streptomycin	100.00	100.0	EXACTP	Intact	Plasmid contig, contig 3	aph(6)-Id (100.00/100.0); aph(3")-Ib (100.00/99.9)
S26	aph(6)-Id	Streptomycin	100.00	100.0	EXACTP	Intact	Plasmid contig, contig 3	aph(6)-Id (100.00/100.0); aph(3")-Ib (100.00/99.9)
S27	emrD	Efflux	99.24	100.0	BLASTP	Intact	Chromosome, contig 1	Not reported
S27	parC_S80I	Quinolone	99.27	98.8	POINTP	Point variant	Chromosome, contig 1	n/a
S27	oqxB19	Phenicol/Quinolone	100.00	100.0	ALLELEP	Intact	Chromosome, contig 1	OqxB (98.57/100.0)
S27	oqxA	Nitrofurantoin/Phenicol/Quinolone/Tigecycline	100.00	100.0	EXACTP	Intact	Chromosome, contig 1	OqxA (98.89/100.0)
S27	gyrA_S83I	Quinolone	99.77	100.0	POINTP	Point variant	Chromosome, contig 1	n/a
S27	ompK36_D135DGD	Carbapenem	92.29	100.0	POINTP	Point variant	Chromosome, contig 1	n/a
S27	blaSHV-28	Beta-Lactam	100.00	100.0	ALLELEP	Intact	Chromosome, contig 1	blaSHV-106 (99.88/100.0); blaSHV-28 (99.88/100.0)
S27	ompK35_Y197Ter	Carbapenem	99.72	100.0	POINTX	Truncated or disrupted	Chromosome, contig 1	n/a
S27	fosA	Fosfomycin	100.00	100.0	EXACTP	Intact	Chromosome, contig 1	fosA6 (99.76/100.0)
S27	blaKPC-3	Carbapenem	100.00	100.0	ALLELEP	Intact	Plasmid contig, contig 2	blaKPC-3 (100.00/100.0)
S27	aac(6')-Ib-cr5	Amikacin/Kanamycin/Quinolone/Tobramycin	100.00	100.0	ALLELEX	Intact	Plasmid contig, contig 2	aac(6')-Ib-cr (100.00/100.0)
S27	blaOXA-1	Cephalosporin	100.00	100.0	ALLELEP	Intact	Plasmid contig, contig 2	blaOXA-1 (100.00/100.0)

Isolate	AMRFinderPlus element	Subclass	Identity (%)	Coverage (%)	Method	Functional status	Location	ResFinder call, identity/coverage (%)
S27	catB3	Chloramphenicol	100.00	70.0	PARTIALP	Truncated or disrupted	Plasmid contig, contig 2	catB3 (100.00/69.8)
S27	tet(A)	Tetracycline	100.00	100.0	EXACTP	Intact	Plasmid contig, contig 2	tet(A) (100.00/100.0)
S27	qnrB1	Quinolone	100.00	100.0	ALLELEP	Intact	Plasmid contig, contig 2	qnrB1 (100.00/100.0)
S27	dfrA14	Trimethoprim	100.00	100.0	EXACTX	Intact	Plasmid contig, contig 2	dfrA14 (100.00/100.0)
S28	emrD	Efflux	99.24	100.0	BLASTP	Intact	Chromosome, contig 1	Not reported
S28	parC_S80I	Quinolone	99.27	98.8	POINTP	Point variant	Chromosome, contig 1	n/a
S28	oqxB19	Phenicol/Quinolone	100.00	100.0	ALLELEP	Intact	Chromosome, contig 1	OqxB (98.57/100.0)
S28	oqxA	Nitrofurantoin/Phenicol/Quinolone/Tigecycline	100.00	100.0	EXACTP	Intact	Chromosome, contig 1	OqxA (98.89/100.0)
S28	gyrA_S83I	Quinolone	99.77	100.0	POINTP	Point variant	Chromosome, contig 1	n/a
S28	ompK36_D135DGD	Carbapenem	92.29	100.0	POINTP	Point variant	Chromosome, contig 1	n/a
S28	blaSHV-28	Beta-Lactam	100.00	100.0	ALLELEP	Intact	Chromosome, contig 1	blaSHV-106 (99.88/100.0); blaSHV-28 (99.88/100.0)
S28	ompK35_Y197Ter	Carbapenem	99.72	100.0	POINTX	Truncated or disrupted	Chromosome, contig 1	n/a
S28	nfsB_H128VfsTer6	Nitrofurantoin	99.54	100.0	POINTX	Truncated or disrupted	Chromosome, contig 1	n/a
S28	fosA	Fosfomycin	100.00	100.0	EXACTP	Intact	Chromosome, contig 1	fosA6 (99.76/100.0)
S28	blaKPC-3	Carbapenem	100.00	100.0	ALLELEP	Intact	Plasmid contig, contig 3	blaKPC-3 (100.00/100.0)

Elements are the AMR-type calls of AMRFinderPlus (chromosomal point variants and acquired genes), and a total of 637 elements were classified as 508 intact, 70 point variants and 59 truncated or disrupted. Identity and coverage are relative to the closest reference sequence. Functional status was assigned as follows. Intact, coverage of at least 90% with a complete-gene method. Truncated or disrupted, a frameshift, premature stop codon or partial-gene call (POINTX, PARTIALP, INTERNAL_STOP). Point variant, an amino acid substitution in a resistance-associated gene. The ResFinder column gives the call at the overlapping contig position with nucleotide identity and coverage (%). "Not reported" indicates that ResFinder (80% identity, 60% coverage) did not report the element, and "n/a" that ResFinder does not report point variants in this table. Location was derived from the assembly contig, which was rotated to dnaA (chromosome) or repA (plasmid contig), and "unclassified" denotes contigs without such a ResFinder annotation.

Supplementary Table S10 (continued). mgrB alignment metrics per isolate from the AMRFinderPlus mutation output and assembly-level alignment result.

Isolate	mgrB coverage (%)	mgrB identity (%)	Method	Non-synonymous change(s) listed	Assembly-level alignment result
S04	100.00	100.00	POINTX	None reported	–
S05	100.00	100.00	POINTX	None reported	–
S06	97.87	100.00	POINTX	None reported	Not available
S07	100.00	100.00	POINTX	None reported	–
S08	97.87	100.00	POINTX	None reported	Not available
S09	100.00	100.00	POINTX	None reported	–
S10	100.00	100.00	POINTX	None reported	–
S11	80.85	100.00	POINTX	None reported	1,066-bp IS insertion after codon 38
S12	100.00	100.00	POINTX	None reported	–
S13	100.00	100.00	POINTX	None reported	–
S14	93.62	100.00	POINTX	None reported	8,162-bp insertion at codon 45
S15	100.00	100.00	POINTX	None reported	–
S16	97.87	97.83	POINTX	mgrB_K2E	Single-adenine deletion in a poly-A tract (codons 2–3), frameshift, premature stop at codon 9
S17	100.00	100.00	POINTX	None reported	–
S18	93.62	100.00	POINTX	None reported	8,162-bp insertion at codon 45
S19	100.00	100.00	POINTX	None reported	–
S20	100.00	100.00	POINTX	None reported	–
S21	100.00	100.00	POINTX	None reported	–
S22	93.62	100.00	POINTX	None reported	8,162-bp insertion at codon 45
S23	100.00	100.00	POINTX	None reported	–
S24	100.00	100.00	POINTX	None reported	–
S25	100.00	100.00	POINTX	None reported	–
S26	100.00	100.00	POINTX	None reported	–
S27	100.00	97.87	POINTX	mgrB_C16R	–
S28	89.36	100.00	POINTX	None reported	p.Lys43fs

Coverage below 100% indicates that the mgrB reference was not covered across its full length. Changes listed as non-synonymous are those in which the reference and observed amino acids differ. Metrics from AMRFinderPlus come from the automated tool output. The last column gives the result of the alignment of the mgrB region extracted from the polished assemblies against the full-length mgrB sequence of isolate S09, which was applied to isolates with colistin-associated calls or incomplete mgrB coverage (S06, S08, S11, S14, S16, S18, S22 and S28). "Not available" indicates that no alignment result was recorded, and "–" that the isolate was not subjected to this alignment. These assembly-level findings were not verified at the read level.

Supplementary Table S11. Integrated isolate-level summary of clonal background, resistance phenotype by antimicrobial class, resistance determinants, carbapenemase-carrying plasmid elements, virulence traits and CRISPR-Cas systems of the 25 carbapenem-resistant Klebsiella pneumoniae isolates.

Isolate (ST, CL)	Resistance phenotype by antimicrobial class								Resistance genes			Chromosomal alterations	Carbapenemase-carrying element(s)	Virulence traits	CRISPR-Cas (arrays, subtype)
	CARB	CEPH	BL/BLI	CZA	AMK	CIP	SXT	CST (MIC)	β-Lactamases	Aminoglycoside-related	qnr and other selected determinants				
S04<br>ST17, CL-4	R	R	R	S	S	R	R	S (0.5)	CTX-M-15, SHV-11, TEM-1	aph(3")-Ib†, aph(6)-Id	qnrB1, sul2, dfrA14, tet(A)	*Colistin:* None<br>*Porin:* point mutations only<br>*QRDR:* none detected	No carbapenemase-carrying element	iuc –, ybt –, clb –<br>Kleborate score 0<br>rmpA/rmpA2: no match	Not detected
S05<br>ST17, CL-4	R	R	R	R	S	R	R	S (0.25)	CTX-M-15, NDM-7, SHV-11, TEM-1	aph(3")-Ib†, aph(6)-Id	qnrB1, sul2, dfrA14, tet(A)	*Colistin:* None<br>*Porin:* point mutations only<br>*QRDR:* none detected	NDM-7: IncX3 element (non-pAA452), 46,161 bp, circular, IncX3, not classified as a plasmid by MOB-suite	iuc –, ybt –, clb –<br>Kleborate score 0<br>rmpA/rmpA2: no match	Not detected
S06<br>ST147, CL-3	R*	R	R	R	R	R	R	R (4)	CTX-M-14, CTX-M-15, NDM-5, OXA-48, OXA-9†, SHV-11, TEM-1	aac(6')-Ib, aadA1, aph(3")-Ib, aph(3')-VI, aph(6)-Id, rmtF1	qnrB1, qnrS1, sul1, dfrA5, dfrA50, mph(A), catA1†, catB, arr-2	*Colistin:* pmrB T157P (AMRFinderPlus)<br>*Porin:* OmpK36 D135DGD ins, OmpK35 R60LfsTer31<br>*QRDR:* gyrA S83I, parC S80I	NDM-5: pAA405, 346,454 bp, circular, IncFIB+IncHI1B, conjugative<br>OXA-48: pAA002, 68,942 bp, circular, IncL/M, conjugative	iuc +, ybt +, clb –<br>Kleborate score 4<br>rmpA/rmpA2: partial, 47%	I-E (41 repeats, 40 spacers)<br>IV-A3 (14 repeats, 13 spacers)
S07<br>ST307, Singleton	R	R	R	R	S	R	R	S (0.25)	CTX-M-14, CTX-M-15, NDM-5, OXA-48, OXA-9†, SHV-28, TEM-1	aac(6')-Ib, aadA1, aph(3")-Ib, aph(3')-VI, aph(6)-Id	qnrB1, qnrS1, sul1, sul2, dfrA5, dfrA14, mph(A), catA1†	*Colistin:* None<br>*Porin:* point mutations only<br>*QRDR:* gyrA S83I, parC S80I	NDM-5 + OXA-48: megaplasmid (joined to chromosomal contig 2), 550,114 bp, circular, IncFIB+IncFIB+IncFII+IncHI1B, chromosomally integrated	iuc +, ybt –, clb –<br>Kleborate score 3<br>rmpA/rmpA2: partial, 55%	IV-A3 (14 repeats, 13 spacers)
S08<br>ST147, CL-3	R	R	R	R	S	R	R	S (0.25)	CTX-M-14, CTX-M-15, NDM-5, OXA-48, OXA-9†, SHV-11, TEM-1	aac(6')-Ib, aadA1, aph(3")-Ib, aph(3')-VI, aph(6)-Id	qnrS1, sul1, dfrA5, dfrA50, mph(A), catA1†	*Colistin:* None<br>*Porin:* OmpK36 D135DGD ins, OmpK35 R60LfsTer31<br>*QRDR:* gyrA S83I, parC S80I	NDM-5: pAA405, 348,579 bp, circular, IncFIB+IncHI1B, conjugative<br>OXA-48: pAA002, 68,942 bp, circular, IncL/M, conjugative	iuc +, ybt +, clb –<br>Kleborate score 4<br>rmpA/rmpA2: partial, 55%	I-E (41 repeats, 40 spacers)<br>IV-A3 (14 repeats, 13 spacers)
S09<br>ST147, CL-1	R	R	R	R	R	R	R	S (0.5)	CTX-M-14, CTX-M-15, NDM-5, OXA-48, OXA-9†, SHV-11, TEM-1	aac(6')-Ib, aadA1, aph(3")-Ib, aph(3')-Ia, aph(3')-VI, aph(6)-Id, rmtF1	qnrS1, sul1, dfrA5, mph(A), catA1†, catB, arr-2	*Colistin:* None<br>*Porin:* OmpK36 D135DGD ins, OmpK35 R60LfsTer31<br>*QRDR:* gyrA S83I, parC S80I	NDM-5: pAA405, 367,492 bp, circular, IncFIB+IncHI1B, conjugative<br>OXA-48: pAA002, 68,942 bp, circular, IncL/M, conjugative	iuc +, ybt +, clb –<br>Kleborate score 4<br>rmpA/rmpA2: partial, 47%	I-E (42 repeats, 41 spacers)<br>IV-A3 (14 repeats, 13 spacers)
S10<br>ST147, CL-1	R	R	R	R	R	R	R	S (0.25)	CTX-M-14, CTX-M-15, NDM-5, OXA-48, OXA-9†, SHV-11, TEM-1	aac(6')-Ib, aadA1, aph(3")-Ib, aph(3')-Ia, aph(3')-VI, aph(6)-Id, rmtF1	qnrS1, sul1, dfrA5, mph(A), catA1†, catB, arr-2	*Colistin:* None<br>*Porin:* OmpK36 D135DGD ins, OmpK35 R60LfsTer31<br>*QRDR:* gyrA S83I, parC S80I	NDM-5: pAA405, 367,492 bp, circular, IncFIB+IncHI1B, conjugative<br>OXA-48: pAA002, 68,942 bp, circular, IncL/M, conjugative	iuc +, ybt +, clb –<br>Kleborate score 4<br>rmpA/rmpA2: partial, 47%	I-E (42 repeats, 41 spacers)<br>IV-A3 (14 repeats, 13 spacers)
S11<br>ST437, Singleton	R	R	R	R	S	R	R	S (1)	CTX-M-14, CTX-M-15, LAP-2, NDM-5, OXA-48, OXA-9†, SHV-11, TEM-1	aac(6')-Ib, aadA1, aph(3')-Ia, aph(3')-VI	qnrS1, sul1, dfrA5, mph(A), catA1†	*Colistin:* None (mgrB 1,066-bp IS insertion after codon 38, colistin-susceptible)<br>*Porin:* OmpK36 D135DGD ins<br>*QRDR:* gyrA S83I, parC S80I	NDM-5: pAA405, 361,371 bp, circular, IncFIB+IncHI1B, conjugative<br>OXA-48: pAA002, 65,499 bp, circular, IncL/M, conjugative	iuc +, ybt +, clb –<br>Kleborate score 4<br>rmpA/rmpA2: partial, 55%	IV-A3 (6 repeats, 5 spacers)
S12<br>ST16, Singleton	R	R	R	S	S	R	R	S (0.25)	CTX-M-15, OXA-48, SHV-1	Not detected	Not detected	*Colistin:* None<br>*Porin:* OmpK36 D135DGD ins, OmpK35 W79Ter<br>*QRDR:* gyrA D87N+S83F, parC E84K	OXA-48: pAA002, 63,589 bp, circular, IncL/M, conjugative	iuc –, ybt –, clb –<br>Kleborate score 0<br>rmpA/rmpA2: no match	Not detected
S13<br>ST307, Singleton	R	R	R	S	R	R	NI	S (0.25)	KPC-3, OXA-1, SHV-28	aac(6')-Ib, aac(6')-Ib-cr	qnrB1, dfrA14, catB3†	*Colistin:* None<br>*Porin:* OmpK36 D135DGD ins, OmpK35 Y197Ter<br>*QRDR:* gyrA S83I, parC S80I	KPC-3: pAA018, 147,568 bp, circular, IncFIB+IncFII (rep2183), conjugative	iuc –, ybt +, clb –<br>Kleborate score 1<br>rmpA/rmpA2: no match	Not detected

Isolate (ST, CL)	Resistance phenotype by antimicrobial class								Resistance genes			Chromosomal alterations	Carbapenemase-carrying element(s)	Virulence traits	CRISPR-Cas (arrays, subtype)
	**CARB**	**CEPH**	**BL/BLI**	**CZA**	**AMK**	**CIP**	**SXT**	**CST (MIC)**	**β-Lactamases**	**Aminoglycoside-related**	**qnr and other selected determinants**				
S14<br>ST147, CL-2	R	R	R	R	R	R	R	R (4)	CTX-M-14, CTX-M-15, NDM-5, OXA-48, OXA-9†, SHV-11, TEM-1	aac(6')-Ib, aadA1, aph(3")-Ib, aph(3')-VI, aph(6)-Id, rmtF1	qnrB1, qnrS1, sul1, dfrA5, dfrA50, mph(A), catA1†, catB, arr-2	*Colistin:* mgrB 8,162-bp insertion at codon 45 (reported by Kleborate as c.T132del)<br>*Porin:* OmpK36 D135DGD ins, OmpK35 R60LfsTer31<br>*QRDR:* gyrA S83I, parC S80I	NDM-5: pAA405, 349,462 bp, circular, IncFIB+IncHI1B, conjugative<br>OXA-48: pAA002, 68,942 bp, circular, IncL/M, conjugative	iuc +, ybt +, clb –<br>Kleborate score 4<br>rmpA/rmpA2: partial, 47%	I-E (44 repeats, 43 spacers)<br>IV-A3 (14 repeats, 13 spacers)
S15<br>ST307, CL-5	R	R	R	R	S	R	R	S (0.25)	KPC-3, SHV-28	Not detected	dfrA14	*Colistin:* None<br>*Porin:* OmpK36 D135DGD ins, OmpK35 Y197Ter<br>*QRDR:* gyrA S83I, parC S80I	KPC-3: pAA018, 69,799 bp, circular, IncFIB, non-mobilizable	iuc –, ybt –, clb –<br>Kleborate score 0<br>rmpA/rmpA2: no match	Not detected
S16<br>ST101, Singleton	R	R	R	R	R	R	R	R (32)	CTX-M-15, NDM-1, OXA-1, OXA-48, SHV-1, TEM-1, TEM-1A	aac(6')-Ib-cr, aac(6')-Ib-cr5, rmtC	sul1, catB3†, arr-3	*Colistin:* mgrB Lys2fs (Kleborate), single-adenine deletion in a poly-A tract confirmed in the assembly. K2E (AMRFinderPlus, unclassified)<br>*Porin:* OmpK35 G62AfsTer1<br>*QRDR:* gyrA D87N+S83Y, parC S80I	NDM-1: pAA046, 109,897 bp, circular, IncFIB+IncFII+rep2272, conjugative<br>OXA-48: pAA002, 64,913 bp, circular, IncL/M, conjugative	iuc –, ybt +, clb –<br>Kleborate score 1<br>rmpA/rmpA2: no match	IV-A3 (17 repeats, 16 spacers)
S17<br>ST307, CL-5	R	R	R	S	S	R	NI	S (0.25)	KPC-3, SHV-28	Not detected	dfrA14	*Colistin:* None<br>*Porin:* OmpK36 D135DGD ins, OmpK35 Y197Ter<br>*QRDR:* gyrA S83I, parC S80I	KPC-3: pAA018, 69,799 bp, circular, IncFIB, non-mobilizable	iuc –, ybt –, clb –<br>Kleborate score 0<br>rmpA/rmpA2: no match	Not detected
S18<br>ST147, CL-2	R	R	R	R	R	R	R	R (4)	CTX-M-15, NDM-5, OXA-9†, SHV-11, TEM-1	aac(6')-Ib, aadA1, aph(3')-VI, rmtF1	qnrB1, qnrS1, sul1, dfrA5, dfrA50, mph(A), catA1†, catB, arr-2	*Colistin:* mgrB 8,162-bp insertion at codon 45 (reported by Kleborate as c.T132del)<br>*Porin:* OmpK36 D135DGD ins, OmpK35 R60LfsTer31<br>*QRDR:* gyrA S83I, parC S80I	NDM-5: pAA405, 351,373 bp, circular, IncFIB+IncHI1B, conjugative	iuc +, ybt +, clb –<br>Kleborate score 4<br>rmpA/rmpA2: partial, 55%	I-E (44 repeats, 43 spacers)<br>IV-A3 (14 repeats, 13 spacers)
S19<br>ST147, CL-2	R	R	R	R	R	R	R	R (8)	CTX-M-14, CTX-M-15, NDM-5, OXA-48, OXA-9†, SHV-11, TEM-1	aac(6')-Ib, aadA1, aph(3")-Ib, aph(3')-Ia, aph(3')-VI, aph(6)-Id, rmtF1	qnrB1, qnrS1, sul1, dfrA5, dfrA50, mph(A), catA1†, catB, arr-2	*Colistin:* pmrB T157P (AMRFinderPlus)<br>*Porin:* OmpK36 D135DGD ins, OmpK35 R60LfsTer31<br>*QRDR:* gyrA S83I, parC S80I	NDM-5: pAA405, 350,811 bp, circular, IncFIB+IncHI1B, conjugative<br>OXA-48: pAA002, 68,942 bp, circular, IncL/M, conjugative	iuc +, ybt +, clb –<br>Kleborate score 4<br>rmpA/rmpA2: partial, 55%	I-E (44 repeats, 43 spacers)<br>IV-A3 (14 repeats, 13 spacers)
S20<br>ST147, CL-2	R	R	R	R	R	R	R	R (8)	CTX-M-14, CTX-M-15, NDM-5, OXA-48, OXA-9†, SHV-11, TEM-1	aac(6')-Ib, aadA1, aph(3")-Ib, aph(3')-Ia, aph(3')-VI, aph(6)-Id, rmtF1	qnrB1, qnrS1, sul1, dfrA5, dfrA50, mph(A), catA1†, catB, arr-2	*Colistin:* pmrB T157P (AMRFinderPlus)<br>*Porin:* OmpK36 D135DGD ins, OmpK35 R60LfsTer31<br>*QRDR:* gyrA S83I, parC S80I	NDM-5: pAA405, 351,638 bp, circular, IncFIB+IncHI1B, conjugative<br>OXA-48: pAA002, 68,942 bp, circular, IncL/M, conjugative	iuc +, ybt +, clb –<br>Kleborate score 4<br>rmpA/rmpA2: partial, 47%	I-E (44 repeats, 43 spacers)<br>IV-A3 (14 repeats, 13 spacers)
S21<br>ST17, CL-4	R	R	R	R	S	R	R	S (0.25)	CTX-M-15, NDM-7, SHV-11, TEM-1	aph(3")-Ib†, aph(6)-Id	qnrB1, sul2, dfrA14, tet(A)	*Colistin:* None<br>*Porin:* point mutations only<br>*QRDR:* none detected	NDM-7: IncX3 element (non-pAA452), 46,161 bp, circular, IncX3, not classified as a plasmid by MOB-suite	iuc –, ybt –, clb –<br>Kleborate score 0<br>rmpA/rmpA2: no match	Not detected
S22<br>ST147, CL-2	R	R	R	R	R	R	R	R (4)	CTX-M-14, CTX-M-15, NDM-5, OXA-48, OXA-9†, SHV-11, TEM-1	aac(6')-Ib, aadA1, aph(3")-Ib, aph(3')-VI, aph(6)-Id, rmtF1	qnrB1, qnrS1, sul1, dfrA5, dfrA50, mph(A), catA1†, catB, arr-2	*Colistin:* mgrB 8,162-bp insertion at codon 45 (reported by Kleborate as c.T132del)<br>*Porin:* OmpK36 D135DGD ins, OmpK36 Q313Ter, OmpK35 R60LfsTer31<br>*QRDR:* gyrA S83I, parC S80I	NDM-5: pAA405, 351,996 bp, circular, IncFIB+IncHI1B, conjugative<br>OXA-48: pAA002, 68,942 bp, circular, IncL/M, conjugative	iuc +, ybt +, clb –<br>Kleborate score 4<br>rmpA/rmpA2: partial, 47%	I-E (44 repeats, 43 spacers)<br>IV-A3 (14 repeats, 13 spacers)

Isolate (ST, CL)	Resistance phenotype by antimicrobial class								Resistance genes			Chromosomal alterations	Carbapenemase-carrying element(s)	Virulence traits	CRISPR-Cas (arrays, subtype)
	CARB	CEPH	BL/BLI	CZA	AMK	CIP	SXT	CST (MIC)	β-Lactamases	Aminoglycoside-related	qnr and other selected determinants				
S23<br>ST147, Singleton	R	R	R	R	R	R	R	S (0.5)	CTX-M-14, CTX-M-15, KPC-3, NDM-5, OXA-1, OXA-48, OXA-9†, SHV-11, TEM-1	aac(6')-Ib, aac(6')-Ib-cr, aac(6')-Ib-cr5, aadA1, aph(3")-Ib, aph(3')-Ia, aph(3')-VI, aph(6)-Id, rmtF1	qnrB1, qnrS1, sul1, dfrA5, dfrA14, dfrA50, tet(A), mph(A), catA1†, catB, catB3†, arr-2	*Colistin:* None<br>*Porin:* OmpK36 D135DGD ins, OmpK35 R60LfsTer31<br>*QRDR:* gyrA S83I, parC S80I	NDM-5: pAA405, 367,492 bp, circular, IncFIB+IncHI1B, conjugative<br>KPC-3: pAA018, 89,256 bp, circular, IncFIB, non-mobilizable<br>OXA-48: pAA002, 68,942 bp, circular, IncL/M, conjugative	iuc +, ybt +, clb –<br>Kleborate score 4<br>rmpA/rmpA2: partial, 47%	I-E (42 repeats, 41 spacers)<br>IV-A3 (14 repeats, 13 spacers)
S24<br>ST147, CL-1	R	R	R	R	R	R	R	S (0.25)	CTX-M-14, CTX-M-15, NDM-5, OXA-48, OXA-9†, SHV-11, TEM-1	aac(6')-Ib, aadA1, aph(3")-Ib, aph(3')-Ia, aph(3')-VI, aph(6)-Id, rmtF1	qnrS1, sul1, dfrA5, dfrA50, mph(A), catA1†, catB, arr-2	*Colistin:* None<br>*Porin:* OmpK36 D135DGD ins, OmpK35 R60LfsTer31<br>*QRDR:* gyrA S83I, parC S80I	NDM-5: pAA405, 367,493 bp, circular, IncFIB+IncHI1B, conjugative<br>OXA-48: pAA002, 68,942 bp, circular, IncL/M, conjugative	iuc +, ybt +, clb –<br>Kleborate score 4<br>rmpA/rmpA2: partial, 55%	I-E (42 repeats, 41 spacers)<br>IV-A3 (14 repeats, 13 spacers)
S25<br>ST147, CL-1	R	R	R	R	R	R	R	S (1)	CTX-M-14, CTX-M-15, NDM-5, OXA-48, OXA-9†, SHV-11, TEM-1	aac(6')-Ib, aadA1, aph(3")-Ib, aph(3')-Ia, aph(3')-VI, aph(6)-Id, rmtF1	qnrS1, sul1, dfrA5, dfrA50, mph(A), catA1†, catB, arr-2	*Colistin:* None<br>*Porin:* OmpK36 D135DGD ins, OmpK35 R60LfsTer31<br>*QRDR:* gyrA S83I, parC S80I<br>*Other:* ramR K104RfsTer6	NDM-5: pAA405, 367,492 bp, circular, IncFIB+IncHI1B, conjugative<br>OXA-48: pAA002, 68,942 bp, circular, IncL/M, conjugative	iuc +, ybt +, clb –<br>Kleborate score 4<br>rmpA/rmpA2: partial, 47%	I-E (42 repeats, 41 spacers)<br>IV-A3 (14 repeats, 13 spacers)
S26<br>ST147, CL-1	R	R	R	R	R	R	R	S (0.25)	CTX-M-14, CTX-M-15, NDM-5, OXA-48, OXA-9†, SHV-11, TEM-1	aac(6')-Ib, aadA1, aph(3")-Ib, aph(3')-Ia, aph(3')-VI, aph(6)-Id, rmtF1	qnrS1, sul1, dfrA5, dfrA50, mph(A), catA1†, catB, arr-2	*Colistin:* None<br>*Porin:* OmpK36 D135DGD ins, OmpK35 R60LfsTer31<br>*QRDR:* gyrA S83I, parC S80I	NDM-5: chromosomally integrated pAA405-like element, 492,660 bp, circular, IncFIB+IncHI1B (chromosomal)<br>OXA-48: pAA002, 68,942 bp, circular, IncL/M, conjugative	iuc +, ybt +, clb –<br>Kleborate score 4<br>rmpA/rmpA2: partial, 60%	I-E (42 repeats, 41 spacers)<br>IV-A3 (14 repeats, 13 spacers)
S27<br>ST307, CL-5	R	R	R	S	S	R	NI	S (0.5)	KPC-3, OXA-1, SHV-28	aac(6')-Ib-cr, aac(6')-Ib-cr5	qnrB1, dfrA14, tet(A), catB3†	*Colistin:* None<br>*Porin:* OmpK36 D135DGD ins, OmpK35 Y197Ter<br>*QRDR:* gyrA S83I, parC S80I	KPC-3: pAA018, 89,256 bp, circular, IncFIB, non-mobilizable	iuc –, ybt –, clb –<br>Kleborate score 0<br>rmpA/rmpA2: no match	Not detected
S28<br>ST307, Singleton	R	R	R	S	S	R	NI	S (0.25)	KPC-3, SHV-28	Not detected	Not detected	*Colistin:* None (mgrB p.Lys43fs, colistin-susceptible)<br>*Porin:* OmpK36 D135DGD ins, OmpK35 Y197Ter<br>*QRDR:* gyrA S83I, parC S80I<br>*Other:* nfsB H128VfsTer6	KPC-3: pAA018, 105,372 bp, circular, IncFIB+IncFII (rep2183), conjugative	iuc –, ybt +, clb +<br>Kleborate score 2<br>rmpA/rmpA2: no match	IV-A3 (19 repeats, 18 spacers)

ST, sequence type. CL, genetic cluster defined at ≤20 allelic differences in the cgMLST analysis (Singleton, not assigned to a multi-isolate cluster). CARB, carbapenems (ertapenem, imipenem, meropenem). CEPH, cephalosporins (cefuroxime, cefoxitin, ceftriaxone, ceftazidime, cefepime). BL/BLI, β-lactam/β-lactamase inhibitor combinations (amoxicillin-clavulanate, piperacillin-tazobactam). CZA, ceftazidime-avibactam. AMK, amikacin. CIP, ciprofloxacin. SXT, trimethoprim-sulfamethoxazole. CST, colistin (broth microdilution MIC in mg/L in parentheses). R, resistant. S, susceptible. Categories follow EUCAST clinical breakpoints v16.1 as in Supplementary Table S2, and the agent-level MIC values are given there. For the class-level columns, R denotes resistance to every agent of the class. R*, S06 was resistant to ertapenem and imipenem and was categorised as susceptible, increased exposure, to meropenem (MIC 8 mg/L). NI, not interpretable, because the automated system reported a lower measurement limit of ≤20 mg/L that could not be categorised according to the EUCAST breakpoint (S13, S17, S27 and S28). Resistance genes were taken from Supplementary Table S3, and the prefix bla is omitted from β-lactamase gene names. † denotes a truncated hit. Chromosomal alterations were taken from Supplementary Table S7. QRDR, quinolone resistance-determining region. Point mutations only indicates that no OmpK35 or OmpK36 insertion or truncation was recorded. The mgrB alterations of S11 and S28 were not accompanied by colistin resistance. The Kleborate call c.T132del in S14, S18 and S22 corresponds to an 8,162-bp insertion within mgrB identified in the assemblies. These assembly-level findings were not validated with read-level data. Element details were taken from Table 3 of the main text, and all listed elements were closed as circular contigs. Plasmid contigs that did not carry a carbapenemase gene are not listed, and the contig-level assembly status of each genome is given in Supplementary Table S5. Virulence traits were taken from Supplementary Table S4. iuc, aerobactin locus (complete iucABCD-iutA operon). ybt, yersiniabactin locus. clb, colibactin locus. The Kleborate virulence score (0–5) was not used as a phenotypic hypervirulence classification. Partial rmpA/rmpA2 matches reported by Kleborate (percentage of the reference sequence covered) were not counted as presence. CRISPR-Cas systems were identified with CRISPRCasTyper v1.8.0, and the subtype, number of repeats and number of spacers (repeats minus one) are given for each trusted CRISPR array. In S28, an additional untrusted array without associated cas genes (III-B-like, 21 repeats) was not counted. Consensus repeat sequences are given in the Results and spacer-level data are given in Supplementary Table S12 and Data S1. Accession numbers (SRA, BioSample, GenBank) are under BioProject PRJNA1538236 and will be added after release.

Supplementary Table S12. CRISPR arrays and spacer content of the 30 trusted CRISPR arrays identified in 17 of the 25 carbapenem-resistant Klebsiella pneumoniae isolates.

Isolate	Subtype	Array	Position (bp)	Repeats	Spacers	Consensus repeat (length)	Mean spacer length (bp)	Spacer profile
S06	I-E	Contig1_1	Contig1: 1,053,506-1,055,972	41	40	GTGTTCCCCGCGCCAGCGGGGATAAACCG (29)	31.9	I-E-A
S06	IV-A3	Contig5_2	Contig5: 32,577-33,396	14	13	GTATTCCCCCCGCATGCGGGGGTTATCG (28)	32.9	IV-A3-ST147
S07	IV-A3	Contig2_1	Contig2: 371,621-372,440	14	13	GTATTCCCCCCGCATGCGGGGGTTATCG (28)	32.9	IV-A3-ST147
S08	I-E	Contig1_1	Contig1: 1,053,672-1,056,138	41	40	GTGTTCCCCGCGCCAGCGGGGATAAACCG (29)	31.9	I-E-A
S08	IV-A3	Contig2_2	Contig2: 33,643-34,462	14	13	GTATTCCCCCCGCATGCGGGGGTTATCG (28)	32.9	IV-A3-ST147
S09	I-E	Contig1_2	Contig1: 1,054,999-1,057,526	42	41	GTGTTCCCCGCGCCAGCGGGGATAAACCG (29)	32.0	I-E-B
S09	IV-A3	Contig2_1	Contig2: 31,422-32,241	14	13	GTATTCCCCCCGCATGCGGGGGTTATCG (28)	32.9	IV-A3-ST147
S10	I-E	Contig1_1	Contig1: 1,054,999-1,057,526	42	41	GTGTTCCCCGCGCCAGCGGGGATAAACCG (29)	32.0	I-E-B
S10	IV-A3	Contig2_2	Contig2: 31,422-32,241	14	13	GTATTCCCCCCGCATGCGGGGGTTATCG (28)	32.9	IV-A3-ST147
S11	IV-A3	Contig2-1_1	Contig2: 32,576-32,903	6	5	AGTATTCCCCCCGCATGCGGGGGTTATCGG (27)	33.0	IV-A3-S11
S14	I-E	Contig1_1	Contig1: 1,052,345-1,054,994	44	43	GTGTTCCCCGCGCCAGCGGGGATAAACCG (29)	32.0	I-E-C
S14	IV-A3	Contig2_2	Contig2: 33,732-34,551	14	13	GTATTCCCCCCGCATGCGGGGGTTATCG (28)	32.9	IV-A3-ST147
S16	IV-A3	Contig2_1	Contig2: 31,133-32,128	17	16	TATTCCCCCCGTGTGCGGGGGTT (23)	37.8	IV-A3-S16
S18	I-E	Contig1_1	Contig1: 1,052,345-1,054,994	44	43	GTGTTCCCCGCGCCAGCGGGGATAAACCG (29)	32.0	I-E-C
S18	IV-A3	Contig2_2	Contig2: 33,732-34,551	14	13	GTATTCCCCCCGCATGCGGGGGTTATCG (28)	32.9	IV-A3-ST147
S19	I-E	Contig1_1	Contig1: 1,053,682-1,056,331	44	43	GTGTTCCCCGCGCCAGCGGGGATAAACCG (29)	32.0	I-E-C
S19	IV-A3	Contig2_2	Contig2: 32,577-33,396	14	13	GTATTCCCCCCGCATGCGGGGGTTATCG (28)	32.9	IV-A3-ST147
S20	I-E	Contig1_1	Contig1: 1,053,682-1,056,331	44	43	GTGTTCCCCGCGCCAGCGGGGATAAACCG (29)	32.0	I-E-C
S20	IV-A3	Contig2_2	Contig2: 32,577-33,396	14	13	GTATTCCCCCCGCATGCGGGGGTTATCG (28)	32.9	IV-A3-ST147

S22	I-E	Contig1_2	Contig1: 1,053,671-1,056,320	44	43	GTGTTCCCCGCGCCAGCGGGGATAAACCG (29)	32.0	I-E-C
S22	IV-A3	Contig2_1	Contig2: 33,732-34,551	14	13	GTATTCCCCCCGCATGCGGGGGTTATCG (28)	32.9	IV-A3-ST147
S23	I-E	Contig1_2	Contig1: 1,055,003-1,057,530	42	41	GTGTTCCCCGCGCCAGCGGGGATAAACCG (29)	32.0	I-E-B
S23	IV-A3	Contig2_1	Contig2: 31,422-32,241	14	13	GTATTCCCCCCGCATGCGGGGGTTATCG (28)	32.9	IV-A3-ST147
S24	I-E	Contig1_2	Contig1: 1,054,999-1,057,526	42	41	GTGTTCCCCGCGCCAGCGGGGATAAACCG (29)	32.0	I-E-B
S24	IV-A3	Contig2_1	Contig2: 31,422-32,241	14	13	GTATTCCCCCCGCATGCGGGGGTTATCG (28)	32.9	IV-A3-ST147
S25	I-E	Contig1_1	Contig1: 1,054,999-1,057,526	42	41	GTGTTCCCCGCGCCAGCGGGGATAAACCG (29)	32.0	I-E-B
S25	IV-A3	Contig2_2	Contig2: 31,422-32,241	14	13	GTATTCCCCCCGCATGCGGGGGTTATCG (28)	32.9	IV-A3-ST147
S26	I-E	Contig1_2	Contig1: 1,054,999-1,057,526	42	41	GTGTTCCCCGCGCCAGCGGGGATAAACCG (29)	32.0	I-E-B
S26	IV-A3	Contig2_1	Contig2: 465,730-466,549	14	13	GTATTCCCCCCGCATGCGGGGGTTATCG (28)	32.9	IV-A3-ST147
S28	IV-A3	Contig2-1_2	Contig2: 29,206-30,322	19	18	GTATTCCCCCCGCATGCGGGGGTTATCGG (28)	32.2	IV-A3-S28

Arrays were identified with CRISPRCasTyper v1.8.0 and only arrays flagged as trusted are listed (n = 30). The number of spacers equals the number of repeats minus one, and spacer sequences are provided in Data S1 (Data_S1_CRISPR_spacers.fasta, 762 spacer sequences with headers in the format isolate|subtype|array|spacer number). Spacer profile denotes groups of arrays with identical spacer sets. I-E-A (40 spacers, S06 and S08), I-E-B (41 spacers, S09, S10, S23, S24, S25 and S26) and I-E-C (43 spacers, S14, S18, S19, S20 and S22) together comprise 43 distinct I-E spacers, and both I-E-A and I-E-B are contained in I-E-C. IV-A3-ST147 denotes the 13 identical spacers shared by the 13 ST147 isolates and S07. The IV-A3 arrays of S11 (5 spacers), S16 (16 spacers) and S28 (18 spacers, 17 distinct) shared no spacer with the IV-A3-ST147 profile, and S11 and S28 shared two spacers with each other. Spacer targets were not examined.
